# Te-Modulated Fe Single Atom with Synergistic Bidirectional Catalysis for High-Rate and Long–Cycling Lithium-Sulfur Battery

**DOI:** 10.1007/s40820-025-01873-3

**Published:** 2025-08-11

**Authors:** Jian Guo, Lu Chen, Lijun Wang, Kangfei Liu, Ting He, Jia Yu, Hongbin Zhao

**Affiliations:** 1 Department of Chemistry, College of Sciences, Shanghai University, Shanghai, 200444 People’s Republic of China; 2 School of Chemical Science and Engineering, Tongji University, Shanghai, 200092 People’s Republic of China; 3 School of Chemical Engineering, East China University of Science and Technology, Shanghai, 200237 People’s Republic of China; 4 Key Laboratory of Precision and Intelligent Chemistry, University of Science and Technology of China, Hefei, 230026 People’s Republic of China

**Keywords:** Single-atom catalyst, Coordination environment, Electronic structure, Bidirectional catalysis, Li-S batteries

## Abstract

Single-atom catalysts (SACs) have garnered significant attention in lithium-sulfur (Li-S) batteries for their potential to mitigate the severe polysulfide shuttle effect and sluggish redox kinetics. However, the development of highly efficient SACs and a comprehensive understanding of their structure–activity relationships remain enormously challenging. Herein, a novel kind of Fe-based SAC featuring an asymmetric FeN_5_-TeN_4_ coordination structure was precisely designed by introducing Te atom adjacent to the Fe active center to enhance the catalytic activity. Theoretical calculations reveal that the neighboring Te atom modulates the local coordination environment of the central Fe site, elevating the d-band center closer to the Fermi level and strengthening the d-p orbital hybridization between the catalyst and sulfur species, thereby immobilizing polysulfides and improving the bidirectional catalysis of Li-S redox. Consequently, the Fe-Te atom pair catalyst endows Li-S batteries with exceptional rate performance, achieving a high specific capacity of 735 mAh g^−1^ at 5 C, and remarkable cycling stability with a low decay rate of 0.038% per cycle over 1000 cycles at 1 C. This work provides fundamental insights into the electronic structure modulation of SACs and establishes a clear correlation between precisely engineered atomic configurations and their enhanced catalytic performance in Li-S electrochemistry.

**Supplementary Information:**

The online version contains supplementary material available at 10.1007/s40820-025-01873-3.

## Highlights

- The Te modulator induces a polarized charge distribution to optimize the electronic structure of the central Fe site, elevating the d-band center and enhancing the density of states near the Fermi level.
- Strengthened d-p orbital hybridization between the catalyst and sulfur species optimizes the adsorption behavior toward LiPSs and facilitates the bidirectional redox process of Li-S batteries.
- The Fe-Te atom pair catalyst endows Li-S batteries remarkable rate performance, extraordinary cycling stability and anticipated areal capacity.

**Supplementary Information:**

The online version contains supplementary material available at 10.1007/s40820-025-01873-3.

## Graphical abstract

**Figure:**
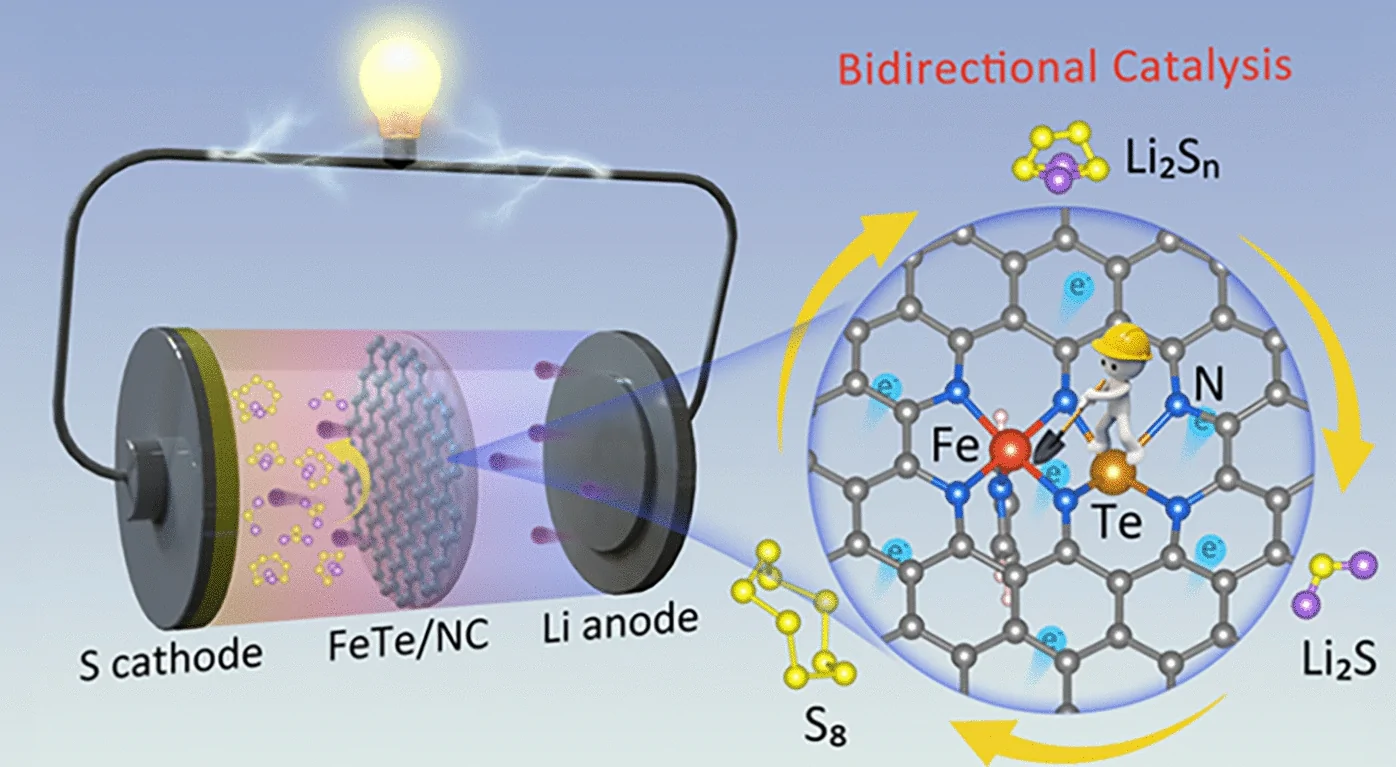

## Introduction

Lithium–sulfur (Li-S) batteries have emerged as a promising alternative to lithium-ion batteries (LIBs) due to their high energy density, cost efficiency, and environmental friendliness [1–5]. However, their practical application is hindered by several critical challenges, including limited sulfur utilization and rapid capacity decay. These issues primarily arise from the intrinsic electrical insulation of sulfur and its discharge products (Li_2_S_2_/Li_2_S), the severe shuttle effect, and the sluggish redox kinetics of the sulfur cathodes [6–10]. On the anode side, uneven lithium deposition aggravates dendrite formation, raising safety concerns [11, 12]. To address these obstacles, extensive efforts have been made to modify separators with carbon-based materials [13, 14]. Unfortunately, their insufficient physical adsorption capacity fails to adequately suppress the shuttle effect, and their limited active sites hinder efficient polysulfide conversion. In response, polar materials rich in chemically active sites, such as metal nanoparticles [15, 16] and metal compounds [17–21], as well as their heterostructures [22, 23], have been exploited to enhance interactions with lithium polysulfides (LiPSs). Nevertheless, the inherent heterogeneity and complexity of these catalysts, including non-uniform physical dimensions and diverse surface configurations, make it challenging to precisely identify the active moieties responsible for catalysis. Therefore, engineering catalysts with well-defined active sites is highly desirable, as it enables a deeper understanding of the correlation between surface structures and specific electrocatalytic activities, providing guidance for the rational structural design of catalysts for advanced Li-S chemistry.

Single-atom catalysts (SACs) with metal-nitrogen/carbon (M-N/C) have recently emerged as promising candidates in the Li-S realm, offering exceptional catalytic activity and maximized atom utilization. More importantly, unlike conventional heterogeneous catalysts, SACs are characterized by atomically dispersed metal centers anchored on a supporting matrix, providing an ideal platform to elucidate the intrinsic correlation between active sites and their catalytic behavior [24–26]. To date, most reported SACs applied in Li-S batteries have been based on a non-polar MN_4_ configuration, but the high electronegativity of the planar-symmetric N atoms surrounding the central metal is not conducive to the adsorption of sulfur species and often increases the reaction energy barriers for LiPSs conversion, adversely affecting the redox kinetics [27, 28]. Given that the catalytic performance of SACs is extremely dependent on the local coordination environment of the central metal atom, great endeavors have been devoted to manipulating the catalytic activity by optimizing the conformation and functionality of the coordinated N species. For example, Chen et al. [29] adjusted the number of coordinated nitrogen atoms around the Fe active site, revealing that the unsaturated-coordination FeN_2_ site exhibited enhanced sulfur immobilization and catalytic performance, as confirmed by detailed physicochemical and electrochemical analyses. Sun et al. [30] engineered the electronic structure of Fe-based SAC by substituting one nitrogen atom in the first coordination shell of FeN_4_ site with P (FeN_3_P_1_), which enhanced LiPSs trapping and conversion due to the increased charge density around the FeN_3_P_1_ configuration in comparison with the FeN_4_ counterpart. While these strategies have proven effective in boosting SAC activity and improving Li-S battery performance, precisely controlling the coordination environment of SACs, especially through heteroatom doping to modulate electronic properties, remains a substantial challenge. Thus, an urgent need exists for the atomic-level regulation of SAC coordination environment and a comprehensive understanding of how electronic structure modifications enhance polysulfide redox kinetics, yet rarely reported up to the present.

Herein, a novel atomically dispersed Fe-based catalyst, with an asymmetric FeN_5_-TeN_4_ coordination structure, was constructed by introducing an adjacent Te atom to the five-coordinated FeN_5_ active moiety on N-doped porous carbon (denoted as FeTe/NC) through a one-step pyrolysis strategy. As expected, the obtained Fe-Te atom pair catalyst exhibits exceptional synergistic effects, which not only enhance the adsorbability of the Fe active center for capturing polysulfides but also facilitate the bidirectional redox process of sulfur. Theoretical calculations illustrate that the Te modulator regulates the electronic structure and micro-environment of the central Fe site by triggering a polarized charge distribution, which enhances the density of states near the Fermi level and elevates the d-band center closer to it, thereby strengthening d-p orbital hybridization between the catalyst and sulfur species and promoting the overall electrochemical performance. Consequently, the assembled Li-S batteries with FeTe/NC-modified separators display satisfactory rate performance and excellent cycle stability, showing an impressive capacity of 735 mAh g^−1^ at 5 C rate and a low capacity fading rate of only 0.038% per cycle over 1000 cycles at 1 C. Beyond that, even with a low E/S ratio of 4.9 μL mg^−1^ and a high sulfur loading of 8.7 mg cm^−2^, the battery still delivers an impressive areal capacity of 5.6 mAh cm^−2^. This work provides profound insight for the design of advanced single-atom catalysts with optimized atomic configurations for high-performance Li-S batteries and deepens the comprehensive understanding of the correlation between structure and catalytic mechanisms at the atomic level.

## Experimental Methods

### Chemicals and Materials

All chemicals, including melamine (A.R. grade), L-alanine (A.R. grade), tellurium dioxide (TeO_2_, A.R. grade), hydrochloric acid (HCl, 37%), and iron trichloride hexahydrate (FeCl_3_·6H_2_O, A.R. grade), were purchased from Adamas-beta. Carbon nanotube (CNT, 99%) was purchased from Nanjing Xianfeng Nanomaterial Technology Co., LTD. Other organic solvents were purchased from Canrd New Energy Co., LTD. All chemicals were used as received without further purification.

### Preparation of FeTe/NC

In a typical synthesis of FeTe/NC, 3 g melamine, 0.75 g L-alanine, 1.5 g TeO_2_, and 7 mg iron trichloride hexahydrate were first ground into a homogeneous precursor for one hour. Following this, a 5 mL mixed solution of ethanol and hydrochloric acid was added. The slurry continued to grind until the ethanol was evaporated. Then, the as-formed yellow product was dried at 60 °C overnight and ground again for 15 min. Subsequently, the resulting product was further carbonized in the tube furnace through a two-stage gentle process with 600 °C for 2 h and then 900 °C for 1.5 h under a N_2_ protective atmosphere. The FeTe/NC-0.75 and FeTe/NC-3 samples were prepared using a similar approach to FeTe/NC, except that 0.75 or 3 g TeO_2_ was used.

### Preparation of Fe/NC and Te/NC

The Fe/NC and Te/NC samples were prepared using a similar approach to FeTe/NC, except that no TeO_2_ or metal salt was added.

More details of other syntheses and characterizations can be seen in Supporting Information.

## Results and Discussion

### Synthetic Strategy and Material Characterizations

**Fig. 1:**
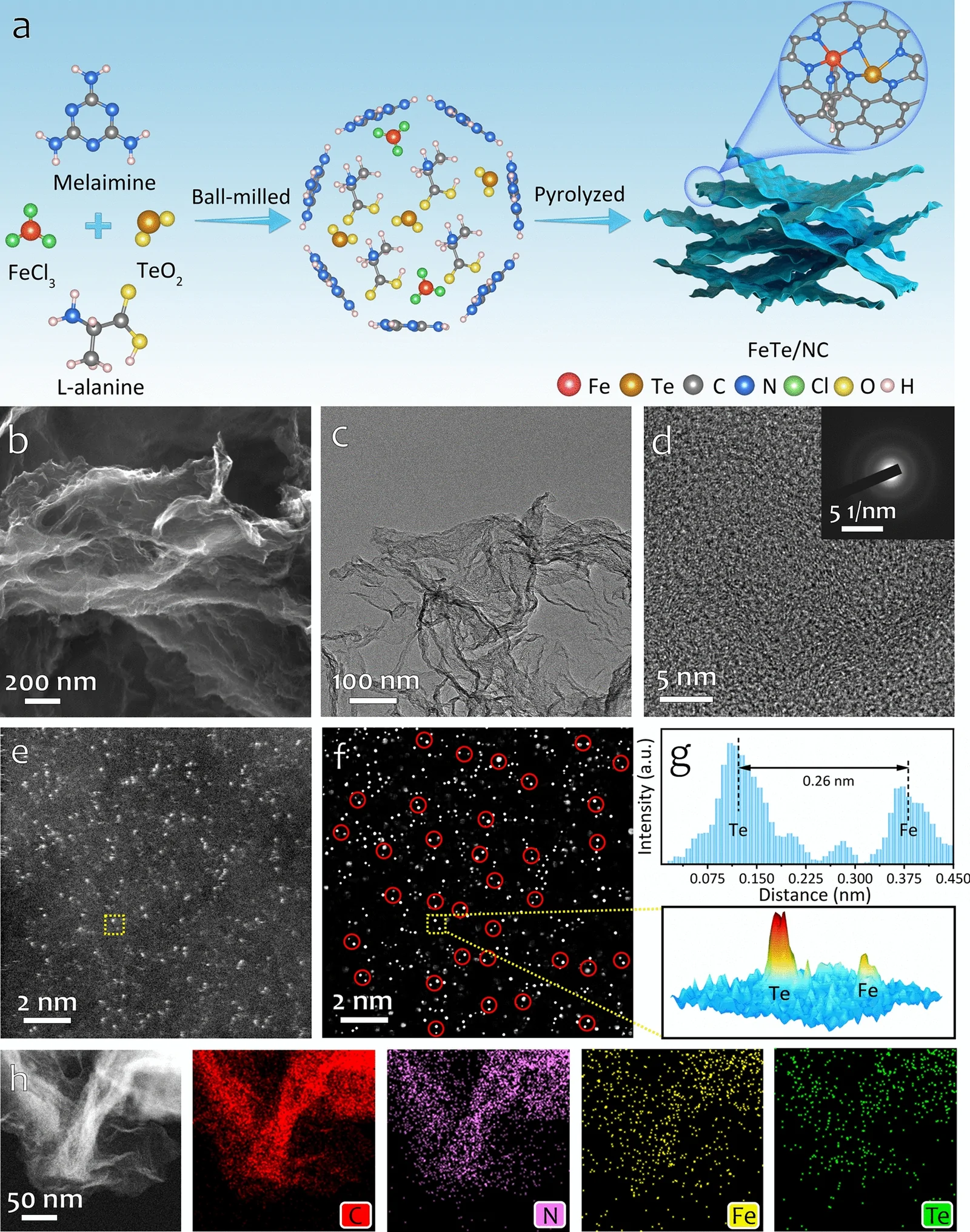
Synthesis scheme and microscopy characterizations of FeTe/NC. **a** Schematic diagram of the synthesis procedure. **b** SEM image, **c** TEM image, **d** HRTEM image (inset: SAED pattern) and **e** AC-HAADF-STEM image of FeTe/NC. **f** Depth learning arithmetic recognition image of (**e**). **g** The intensity distribution and a 3D model of Fe-Te bright spots aligned with the yellow dotted line. **h** HAADF-STEM image and corresponding elemental mappings of FeTe/NC

Figure 1a illustrates the typical procedure for synthesizing FeTe/NC. Briefly, melamine, L-alanine, FeCl_3,_ and TeO_2_ were ball-milled into a uniform precursor, which was successively pyrolyzed at 600 and 900 °C under a nitrogen atmosphere. This process yielded an atomic catalyst with a FeN_5_-TeN_4_ coordination structure anchored on a nitrogen-doped carbon matrix. For comparison, the Fe/NC with a FeN_5_ coordination structure and Te/NC with a TeN_3_ coordination structure were synthesized via a similar approach, with TeO_2_ or metal salt omitted as appropriate. The morphology and structure of FeTe/NC were characterized by scanning electron microscope (SEM) and transmission electron microscopy (TEM). As observed in Fig. 1b, c, the FeTe/NC maintains an ultrathin nanosheet morphology with a wrinkled surface. Similarly, Fe/NC and Te/NC exhibit a comparable morphology to that of FeTe/NC (Figs. S1 and S2). Brunauer–Emmett–Teller (BET) analysis indicates that FeTe/NC possesses a high specific surface area (217 m^2^ g^−1^) with a large number of mesopores, significantly exceeding the value for Fe/NC (177 m^2^ g^−1^) (Fig. S3), which promotes the mass transport and the exposure of more accessible active sites. The high-resolution transmission electron microscopy (HRTEM) image and selected area electron diffraction (SAED) pattern (Fig. 1d) confirm the amorphous nature of FeTe/NC, consistent with the X-ray diffraction (XRD) pattern, which shows the presence of a pure carbon matrix without any detectable metal nanoparticles (Fig. S4). Aberration-corrected high-angle annular dark-field scanning transmission electron microscopy (AC-HAADF-STEM) was performed to visualize the single atoms (Fig. 1e). It is noteworthy that a large number of isolated bright dots are randomly distributed on the carbon matrix, indicating the atomic dispersion of Fe and Te species. To confirm the existence of Fe and Te atoms, the AtomSegNet App [31] was employed for super-resolution processing and atomic feature tracking in the AC-HAADF-STEM image. The FeTe/NC displays two distinct types of bright spots, with the larger ones corresponding to Te atoms and the smaller ones to Fe atoms. Notably, most of these bright spots appear in pairs, as marked by red circles in Fig. 1f. The intensity profiles along the yellow dotted lines, combined with statistical analysis of site distributions based on the distances between adjacent spots from 60 pairs, reveal that most of the observed paired spots have an interatomic distance of ~ 0.26 nm (Figs. 1g and S5). This indicates that the neighboring single atoms combine to create dual-atom sites rather than discrete single-atom sites. HAADF-STEM image and corresponding energy-dispersive spectroscopy (EDS) elemental mappings confirm the uniform distribution of C, N, Fe, and Te elements throughout the entire architecture of FeTe/NC (Fig. 1h). Specifically, the contents of Fe and Te in FeTe/NC were measured to be 1.54 wt% and 2.92 wt% by inductively coupled plasma optical emission spectrometry (ICP-OES) (Table S1). Raman spectrum of FeTe/NC shows two typical D and G bands of carbon at 1355 and 1572 cm^−1^, respectively (Fig. S6). The I_D_/I_G_ ratio for FeTe/NC is 1.03, higher than that of Fe/NC (1.01), suggesting a high degree of structural disorder and defects, which is possibly due to the in-situ incorporation of Te atoms [32].

**Fig. 2:**
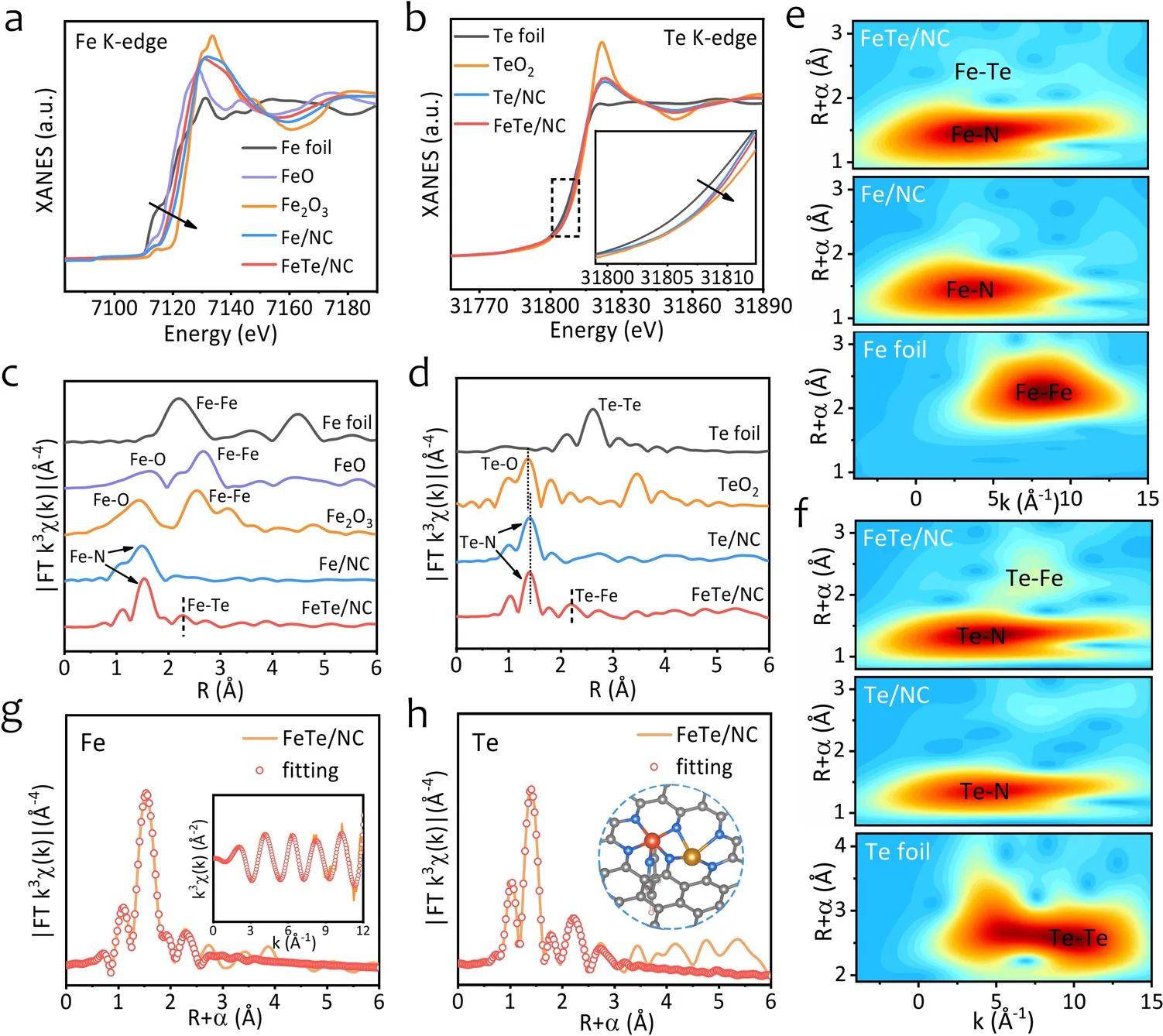
X-ray adsorption spectroscopy results for FeTe/NC and the comparison samples. **a** Fe K-edge XANES. **b** Te K-edge XANES. **c,** **d** k^3^-weighted FT-EXAFS spectra of Fe and Te at R spaces. **e,** **f** WT plots of FeTe/NC in comparison with the Fe/NC, Te/NC, Fe foil, and Te foil. **g** FT-EXAFS fitting curve in R and k spaces of FeTe/NC at the Fe K-edge. **h** FT-EXAFS fitting curve in R space of FeTe/NC at the Te K-edge (inset: the schematic model of FeTe/NC)

X-ray photoelectron spectroscopy (XPS) was conducted to analyze the elemental composition and valence of different samples. The survey further confirms the existence of Fe, Te, N, and C elements (Fig. S7). As presented in Fig. S8, the high-resolution XPS spectrum of FeTe/NC shows three peaks for Fe 2*p*, in which the peaks at 709.6, 712.8, and 722.6 eV could be ascribed to Fe^2+^ 2*p*_3/2_, Fe^3+^ 2*p*_3/2_, and Fe^2+^ 2*p*_1/2_, respectively [33]. In the Te 3*d* spectrum, the peaks located at 576.2 and 586.5 for the Te 3*d*_5/2_ and Te 3*d*_3/2_ correspond to Te^4+^, and the peaks centered at 574.0 and 584.4 of Te 3*d*_5/2_ and Te 3*d*_3/2_ can be considered as Te^δ+^ since the binding energy of metallic Te^0^ is 573.0 eV [34]. The high-resolution C 1*s* spectrum exhibits three main peaks at 284.6, 285.5, and 287.3 eV, corresponding to C–C/C=C, C–O/C=N and C=O/C–N, respectively [35]. The N 1*s* spectrum can be deconvoluted into five peaks, which are attributed to pyridinic N (398.2 eV), metal N (399.4 eV), pyrrolic N (400.7 eV), graphitic N (401.5 eV), and oxidized N (403.2 eV) [36]. It is noteworthy that compared with Fe/NC, after introducing Te, the peaks of Fe 2*p* show a negative shift with lower binding energy, suggesting a strong interaction and electron transfer between Te and Fe atoms. Additionally, X-ray absorption spectroscopy (XAS) was employed to probe into the electronic structure and local coordination information of the Fe-Te atom pair sites in FeTe/NC, and comparisons were made with Fe/NC, Te/NC and several standard samples. The Fe K-edge X-ray absorption near-edge structure (XANES) spectra (Fig. 2a) indicate that the absorption threshold of FeTe/NC and Fe/NC is located between FeO and Fe_2_O_3_, indicating the average valence state of Fe falls between +2 and +3. Notably, the FeTe/NC shows a lower absorption edge position compared to Fe/NC, which can be attributed to the polarized charge distribution around the Fe site induced by the incorporation of Te. For the Te K-edge, the adsorption edges of FeTe/NC and Te/NC are between those of Te foil (0) and TeO_2_ (+4), indicating the positively charged feature of Te (Fig. 2b). As shown in Fig. 2c, the Fourier-transformed k^3^-weighted extended X-ray absorption fine structure (FT-EXAFS) spectra of both Fe/NC and FeTe/NC show a primary peak at ≈1.54 Å, which can be attributed to Fe-N in the first coordination shell, indicating the atomic dispersion feature of Fe atoms. In addition, a minor peak at about 2.28 Å suggests the presence of Fe-Te coordination. Meanwhile, the Te spectrum displays a primary peak at 1.41 Å and a secondary peak at 2.21 Å, attributed to Te-N and Te-Fe coordination, respectively (Fig. 2d), further revealing the existence of a double-atomic structure. Wavelet transform (WT) contour plots of the EXAFS profiles were carried out to further distinguish the backscattering atoms (Figs. 2e, f and S9). The WT contour maxima for FeTe/NC at the Fe K-edge and Te K-edge occur near 3.8 and 4.1 Å^−1^, ascribed to Fe-N and Te-N coordination, respectively. Additionally, secondary maxima appear at 4.3 and 7.4 Å^−1^, attributed to Fe-Te and Te-Fe scattering paths. No Fe-Fe or Te-Te signals were observed compared to the contour plots of Fe foil and Te foil. To determine the structural parameters and precise chemical configuration of Fe and Te atoms, quantitative least-squares EXAFS curve-fitting analysis was conducted (Figs. 2g, h and S10-S12). The fitting results for FeTe/NC (Table S2) indicate that Fe is coordinated with five N atoms and one Te atom, and the Te-N coordination number is 4, forming a FeN_5_-TeN_4_ coordination structure, as illustrated in the inset of Fig. 2h [37]. These results not only confirm the co-existence of atomically dispersed Fe and Te but also validate the well-defined structure of FeTe/NC.

### Catalytic-Enhanced Adsorption and Sulfur-redox Kinetics

**Fig. 3:**
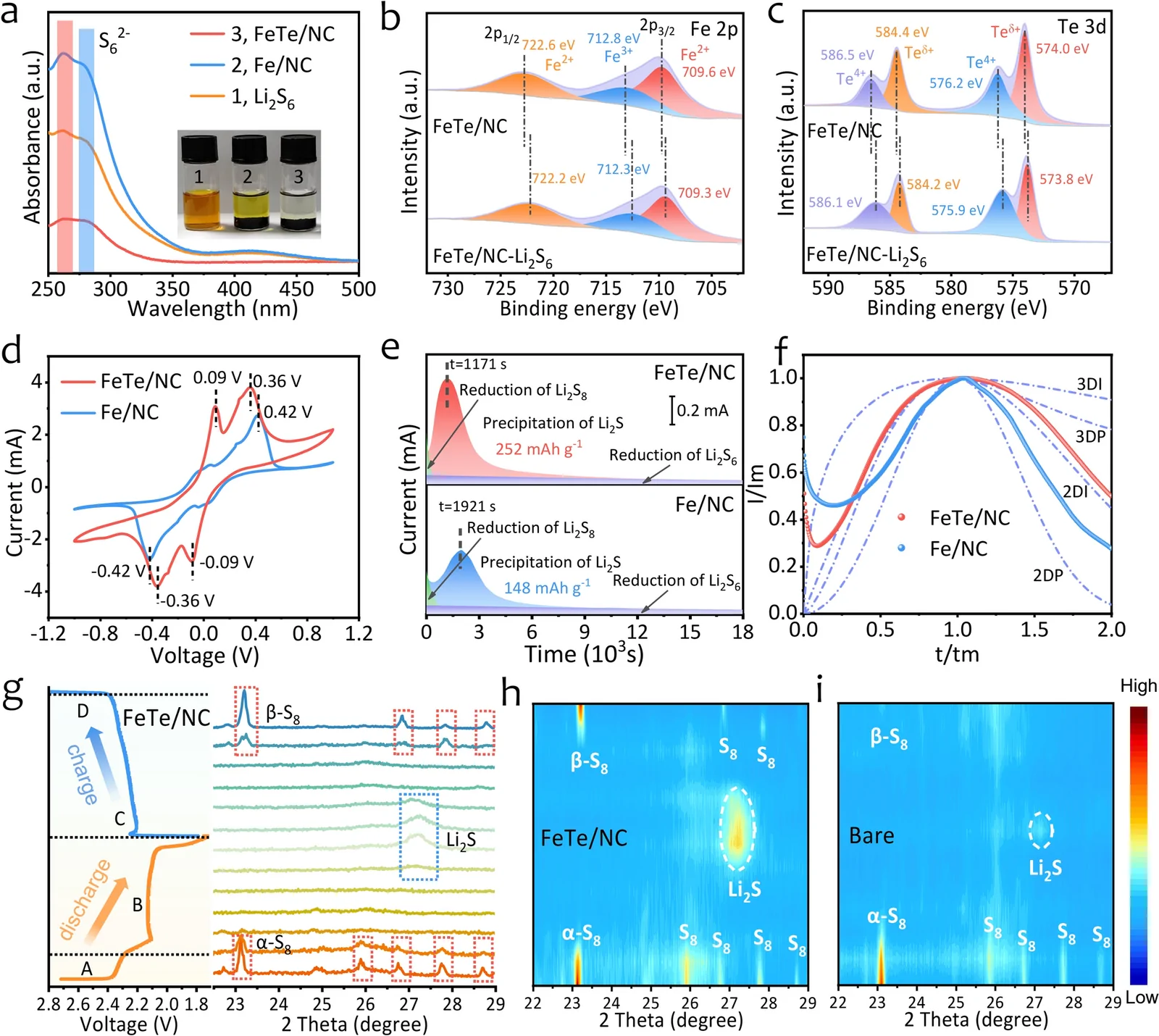
Catalytic-induced strong adsorption and fast polysulfide conversion. **a** UV-Vis spectra of Li_2_S_6_ solution containing different adsorbents (inset: photos of FeTe/NC and Fe/NC soaked in Li_2_S_6_ solution after 6 h). **b,** **c** Fe 2*p* and Te 3*d* peaks for the FeTe/NC before and after Li_2_S_6_ adsorption. **d** CV curves of symmetric cells. **e** Potentiostatic discharge profiles at 2.09 V and **f** corresponding dimensionless transient and theoretical models. **g** In-situ XRD patterns with the corresponding voltage profiles and **h,** **i** contour plots of the FeTe/NC and bare PP-based Li-S cells during the initial cycle

The adsorptive performance of the catalysts toward polysulfides is critical for suppressing the shuttle effect. Therefore, a visualized adsorption test and UV-Vis spectroscopy analysis were performed with a 5 mmol Li_2_S_6_ solution (Fig. 3a). After standing for 6 hours, the orange Li_2_S_6_ solution with the addition of FeTe/NC almost turned transparent, while the solutions with Fe/NC retained a yellow color, as also reflected by the UV-Vis spectra. The sharp contrast confirms the favorable immobilization of sulfur species on FeTe/NC. Furthermore, the underlying chemical interactions of the absorber with LiPSs were investigated *via* XPS analysis. The Fe 2*p* and Te 3*d* XPS spectra of FeTe/NC before and after Li_2_S_6_ adsorption are shown in Fig. 3b, c. After adsorbing Li_2_S_6_, all peaks of Fe 2*p* and Te 3*d* shift toward lower binding energies, indicating a strong chemical interaction between FeTe/NC and Li_2_S_6_ [38]. Besides, the N1s, Li 1*s* and S 2*p* spectra reveal the formation of Fe-S and Li-N bonds (Fig. S13), which further confirm the strong affinity of FeTe/NC toward LiPSs [35, 39]. To evaluate the catalytic conversion capabilities of different materials for polysulfides, cyclic voltammetry (CV) measurements were conducted on symmetrical cells with various electrodes in a Li_2_S_6_ electrolyte. The CV curves of Li_2_S_6_ symmetric cells exhibit two pairs of reversible redox peaks in a voltage range of  −1.0 ~ 1.0 V (Fig. 3d). Notably, FeTe/NC displays a higher redox current response and smaller polarization potential compared to Fe/NC. Even with increasing scan rates, the symmetrical cell with FeTe/NC electrode can also exhibit a higher peak current, indicating a superior catalytic activity for LiPSs conversion (Fig. S14). The results were further validated *via* the electrochemical impedance spectroscopy (EIS) analysis, in which the FeTe/NC possesses the lowest interfacial charge transfer resistance (Fig. S15 and Table S3).

The catalytic properties of FeTe/NC for LiPSs redox are also evident in the deposition and dissolution process of Li_2_S. As shown in Fig. 3e, the FeTe/NC delivers a faster current response toward Li_2_S nucleation compared to that of Fe/NC, and the calculated capacity of Li_2_S nucleation on FeTe/NC is 252 mAh g^−1^, significantly exceeding that of Fe/NC (148 mAh g^−1^), showcasing a notable lower nucleation overpotential and improved kinetics for Li_2_S deposition on FeTe/NC. To understand the different Li_2_S growth behaviors on different surfaces, a dimensionless analysis of the current*-*time curves from Li_2_S nucleation tests was conducted using the Scharifker-Hills model (Eqs. (S1-S4)) [40, 41]. For 2D progressive nucleation (2DP) and 2D instantaneous deposition (2DI), the nucleation rates are predominantly governed by the crystal phase, while the nucleation rates of 3D progressive nucleation (3DP) and 3D instantaneous deposition (3DI) are primarily influenced by ion diffusion, which could significantly accelerate the nucleation rate of Li_2_S [41]. As depicted in Fig. 3f, for Fe/NC, the Li_2_S growth follows a hybrid 2DI and 2DP model, while for FeTe/NC, the growth mode transforms to a hybrid 3DP and 2DI mode, indicating a higher nucleation rate of Li_2_S on the FeTe/NC surface compared to Fe/NC on account of the abundant and monodispersed Fe and Te atoms. In addition to the Li_2_S deposition process, the dissolution of Li_2_S is equally crucial, as rapid oxidation of Li_2_S during the charging process can prevent the accumulation of inactive Li_2_S and blockages at the active sites. As shown in Fig. S16, the FeTe/NC catalyst exhibits a higher dissolution current response and a larger dissolution capacity of 387 mAh g^−1^, suggesting a reduced energy barrier for Li_2_S oxidation and significant kinetic enhancement due to the electronic modulation by Te atoms.

Subsequently, the synthesized FeTe/NC and Fe/NC were successfully coated on the surface of bare polypropylene (PP) separators, as confirmed by digital photographs, SEM, and cross-sectional SEM images (Figs. S17-S20). The bare PP separator displays a porous, network-like structure that permits ion transport while blocking electron transfer, enabling soluble LiPSs to pass through the separator easily. In contrast, the FeTe/NC coating uniformly covers the submicrometer pores of the bare PP separator with a thickness of 7 μm, creating efficient channels for Li^+^ transport. Besides, the FeTe/NC-modified separator exhibits the highest tensile strength among the three samples (Fig. S21), which can be attributed to its dense nanostructure and strong interfacial interaction, enhancing the mechanical integrity of the separator. Contact angle (CA) measurements (Fig. S22) indicate superior electrolyte wettability of the FeTe/NC-modified separator, with a CA of 3.1°, markedly lower than those of Fe/NC (9.6°) and bare PP (30.1°) separators. The improved wettability promotes electrolyte absorption and facilitates Li^+^ permeation and transport. The lithium-ion transfer number of FeTe/NC-modified separator is 0.79, higher than that of Fe/NC (0.68) and bare PP (0.45) separators, which shows the excellent transport ability of lithium ions and lays a foundation for high-rate rechargeable Li-S battery (Fig. S23). Furthermore, as illustrated in Fig. S24, LiPSs readily diffuse across the bare PP separator within 24 h, while the FeTe/NC-modified separator effectively suppresses LiPSs diffusion during the same period, indicating unprecedented LiPSs blocking capability.

To evaluate the electrocatalytic effect of the as-prepared catalysts on overall electrochemical performance, Li-S coin cells were assembled using various catalysts as the separator modifiers, with the carbon nanotube/sulfur composite as the cathode (70 wt% sulfur content, as shown in Fig. S25). The SEM and elemental mapping images reveal a uniform distribution of sulfur across the CNT network, ensuring efficient electron conduction and effective contact with the separator interface (Fig. S26). In-situ XRD was then employed to investigate the electrocatalytic behavior of FeTe/NC throughout the whole discharge and charge process. Figure 3g-i reveals that the FeTe/NC-based Li-S cell achieved highly reversible S_8_ ↔ Li_2_S conversion. Notably, Li_2_S nucleation and dissociation occurred early in regions B and C, respectively. In contrast, the bare PP-based cell displayed prolonged S_8_ peaks throughout the initial discharge stage, with delayed Li_2_S nucleation and dissociation (later stages of B and C) and weaker Li_2_S peak intensity due to sluggish redox activity (Fig. S27). This highlights the synergistic catalytic effect of Fe-Te atom pair sites in FeTe/NC, which facilitates the rapid conversion of LiPSs and enhances sulfur utilization, thereby significantly improving the overall electrochemical performance.

### Kinetic Analysis of Polysulfide Conversion

**Fig. 4:**
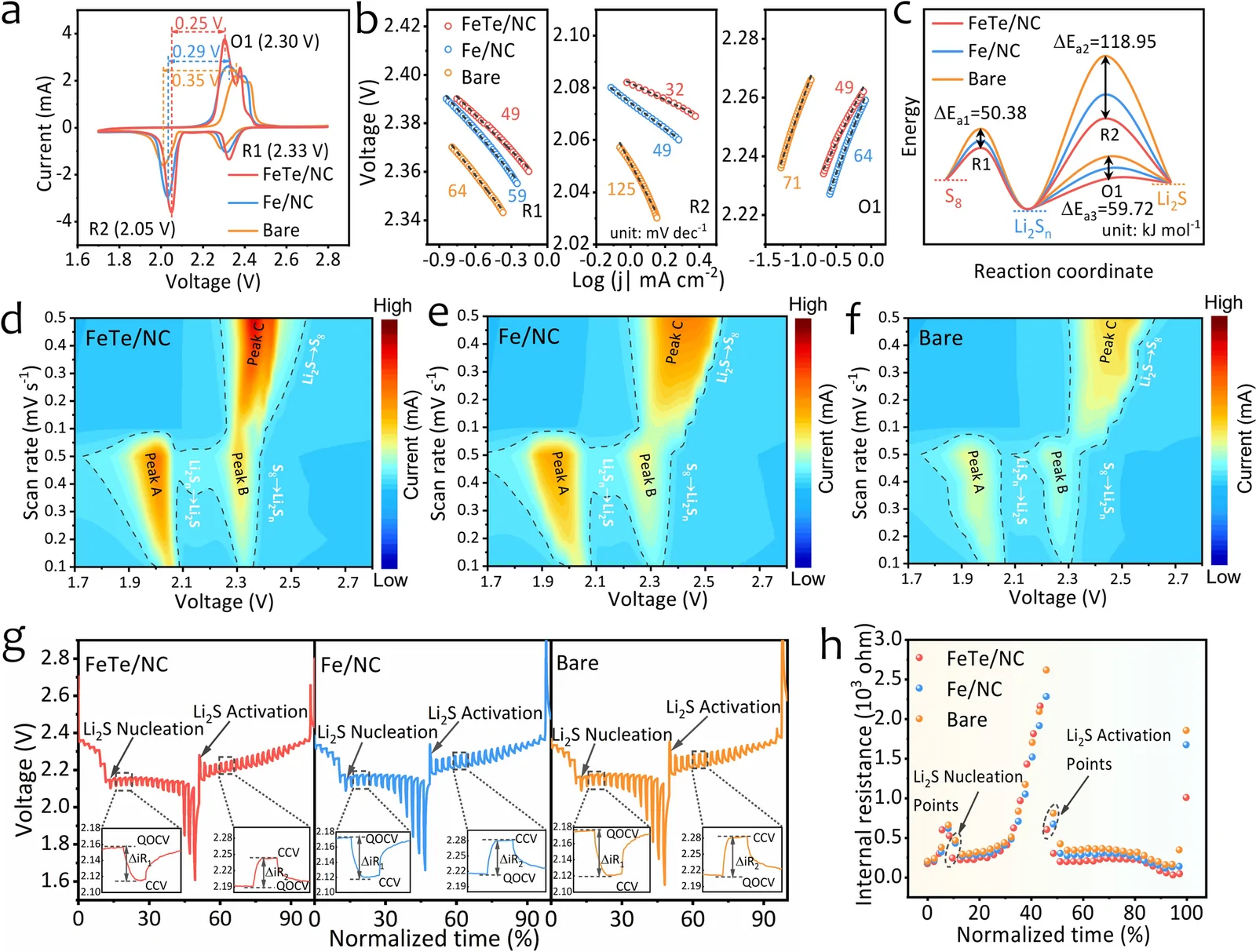
Kinetic analysis of enhanced polysulfide conversion. **a** CV curves at a scan rate of 0.1 mV s^−1^ and **b** the corresponding Tafel plot. **c** Activation energies in the reduction and oxidation process. **d-f** Counter maps of CV curves at different scan rates. **g** GITT curves and **h** corresponding internal resistances of different samples

Figure 4a displays the CV curves recorded at a scan rate of 0.1 mV s^−1^ within a potential window of 1.7–2.8 V. All curves exhibit two reduction peaks, corresponding to the conversion of sulfur to soluble LiPSs and further to Li_2_S and Li_2_S_2_. Additionally, two close oxidation peaks at ≈ 2.40 V are associated with the reverse process of Li_2_S/Li_2_S_2_ to sulfur. Notably, in comparison with Fe/NC and bare PP, the cell using FeTe/NC-modified separator demonstrates the highest peak current density and the lowest potential polarization, implying faster redox kinetics toward LiPSs conversion in the presence of FeTe/NC atomic mediator. Accordingly, the Tafel plots obtained from the CV curves are presented in Fig. 4b. The FeTe/NC-based cell possesses much smaller slope values during the whole redox process, further manifesting its remarkable bidirectional catalytic activity. According to Tafel slopes, the difference in activation energy (${E}_{a}$) is evaluated using Eq. (S5) [42]. As displayed in Fig. 4c, the FeTe/NC catalyst presents significantly lower activation energies, with the decreased ${E}_{a}$ values being 50.38 kJ mol^−1^ (ΔE_a1_) for the reduction of S_8_ to Li_2_S_n_ (4 ≤ n ≤ 8) (R1), 118.95 kJ mol^−1^ (ΔE_a2_) for the conversion of Li_2_S_n_ to Li_2_S (R2), and 59.72 kJ mol^−1^ (ΔE_a3_) for the oxidation of Li_2_S (O1). The decrease in activation energy demonstrates an accelerated redox reaction rate, highlighting the promising catalysis behavior of the Te-modulated Fe active sites in FeTe/NC. Besides, the CV measurements at multiple scan rates were employed to further evaluate the Li^+^ diffusion dynamics. As illustrated in Figs. 4d-f and S28, with an increase in the scan rate, the FeTe/NC-based cell exhibited a smaller peak shift and higher current response in contrast to the counterparts, especially in the region where Li_2_S_2_/Li_2_S transitions to LiPSs and S_8_. Meanwhile, the Li^+^ diffusion coefficient (D_Li_^+^) was calculated qualitatively using the Randles–Sevcik equation (Eq. (S6)) [43]. As shown in Fig. S29, Tables S4 and S5, the FeTe/NC-based cell exhibited a significantly larger quantity of D_Li_^+^ in each reaction step. This can be ascribed to the introduction of Te atoms and the unique FeN_5_-TeN_4_ coordination structure, which ensures better contact with the electrolyte and accelerates Li^+^ diffusion behavior. The EIS in Fig. S30 also shows that the cell with the FeTe/NC-modified separator exhibits lower R_ct_ than the counterparts, indicating accelerated electron and ion transport during the interfacial sulfur redox process (Table S6). Moreover, the elevated ion diffusion and sulfur redox kinetics of the FeTe/NC-based cell were further confirmed by galvanostatic intermittent titration technique (GITT) measurement. The internal resistance of Li_2_S nucleation and activation is reflected in the depth of the dips observed in the discharge and charge profiles (marked by the dotted rectangle in Fig. 4g). The polarization during the discharge–charge cycle can be evaluated using the relative values of ΔR_internal_ derived from the GITT tests (Eq. (S7)) [44]. As shown in Fig. 4h, the FeTe/NC-based cell demonstrated the lowest internal resistance at the points of Li_2_S nucleation and activation compared to other cells. This result suggests that the FeTe/NC facilitates rapid Li_2_S nucleation and activation, thereby greatly improving the redox reactions.

### Electrochemical Properties and Battery Performance

**Fig. 5:**
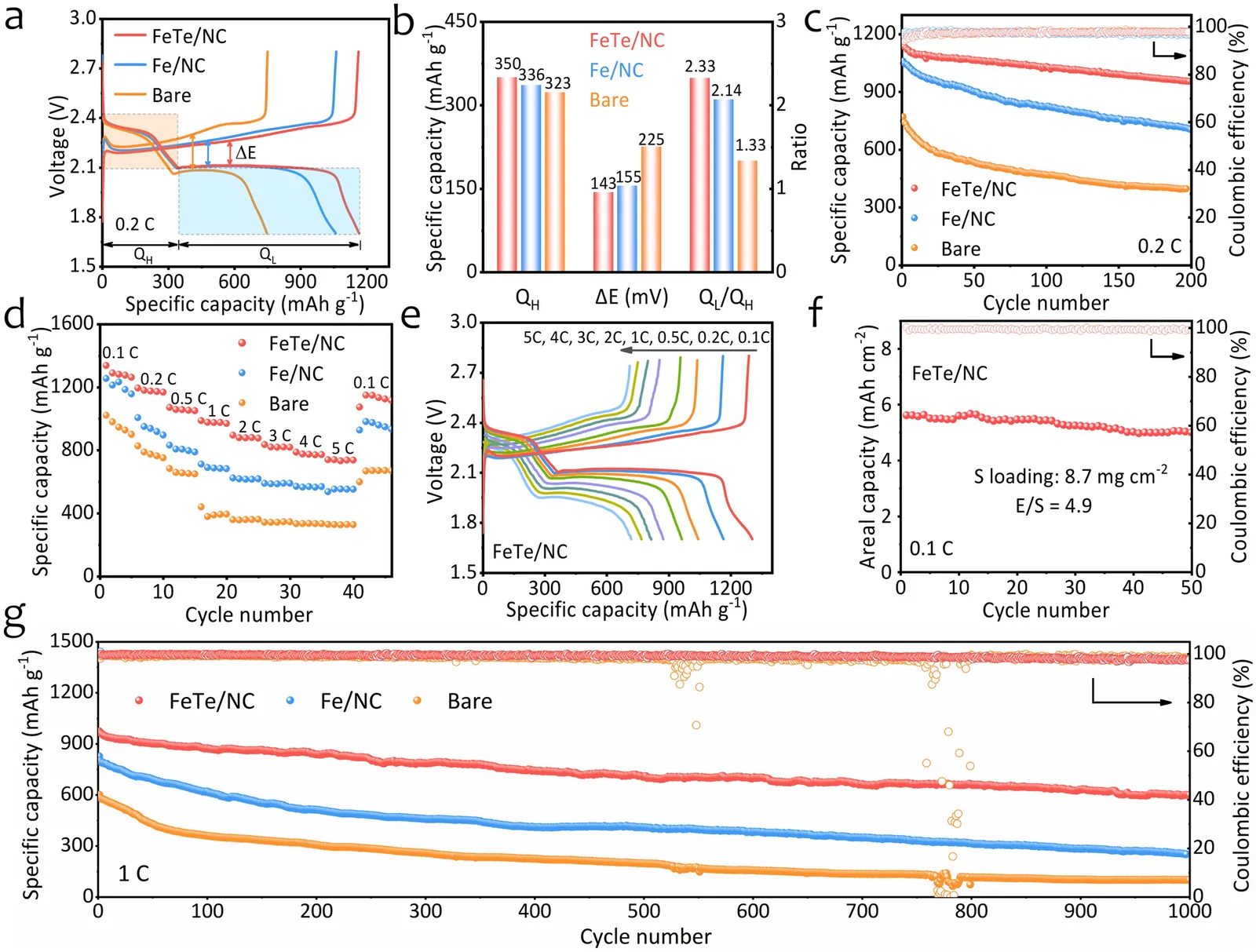
Electrochemical performance of Li-S cells. **a** GCD curves at 0.2 C and **b** corresponding capacity contributions of Q_H_, ΔE, and Q_L_/Q_H_ values. **c** Cycling performance at 0.2 C. **d** Rate performance and **e** the associated charge–discharge plots. **f** Cycling performance at 0.1 C under a high sulfur loading of 8.7 mg cm^−2^. **g** Long-term cycling performance of FeTe/NC, Fe/NC, and bare PP-based cell

To further evaluate the electrochemical performance, galvanostatic charge/discharge (GCD) profiles of the cells with different separators are shown in Fig. 5a. Notably, the cell with FeTe/NC-modified separator exhibits the smallest voltage hysteresis (ΔE = 143 mV) and the highest initial discharge capacity (1149 mAh g^−1^) compared to those with Fe/NC (155 mV, 1060 mAh g^−1^) and bare PP (225 mV, 770 mAh g^−1^). The ratio of the capacity in the low plateau (Q_L_) to that in the high plateau (Q_H_) (Q_L_/Q_H_) of the cells with different separators was further evaluated, as it is a key reference for assessing the catalytic performance in the conversion from soluble polysulfides to Li_2_S (Fig. 5b). It is found that the FeTe/NC-based cell displays a much higher Q_L_/Q_H_ value compared to the Fe/NC and bare PP-based cells. A higher Q_L_/Q_H_ value indicates better catalytic activity, confirming the exceptional electrocatalytic capability of FeTe/NC in enhancing the effective sulfur redox processes. The cycling performance of Li-S cells employing different separators at 0.2 C is presented in Fig. 5c. The cell with FeTe/NC-modified separator shows an initial specific capacity of 1149 mAh g^−1^ and retained a substantial specific capacity of 954 mAh g^−1^ after 200 cycles, representing a commendable retention of 83.0%. In contrast, the Fe/NC and bare PP-based cells show lower initial capacities of 1060 and 770 mAh g^−1^, respectively, with capacity retentions of only 66.4% and 51.4% after 200 cycles. As expected, the FeTe/NC-based cell exhibits excellent rate performance. As shown in Fig. 5d, the FeTe/NC-based cell delivers impressive reversible capacities of 1281, 1171, 1061, 971, 883, 822, 773, and 735 mAh g^−1^ at 0.1, 0.2, 0.5, 1, 2, 3, 4, and 5 C, respectively, outperforming the Fe/NC and bare PP counterparts. When the current density is switched back to 0.1 C, a reversible capacity up to 1131 mAh g^−1^ is still retained, implying the excellent reversibility of the FeTe/NC-based cell. Meanwhile, the corresponding GCD curves at various rates are shown in Figs. 5e and S31, in which the flat and stable charge–discharge voltage plateaus are well maintained for the FeTe/NC-based cell upon the increase of current rate. Moreover, the performance under high sulfur loading and low electrolyte usage was evaluated to assess the potential application of the FeTe/NC catalyst in high-energy-density Li-S batteries. As shown in Fig. 5f, the areal capacity of the FeTe/NC-based cell achieves 5.6 mAh cm^−2^ with a high sulfur loading of 8.7 mg cm^−2^ and low electrolyte to a sulfur ratio (E/S) of 4.9 μL mg^−1^, exceeding the state-of-the-art LIBs (4 mAh cm^−2^). A long-term cycling stability test further validates the durability of the FeTe/NC-based cell (Fig. 5g). Upon cycling at 1 C, the cell with the FeTe/NC-modified separator still maintained a high reversible capacity of 601 mAh g^-1^ after 1000 cycles, with a low capacity fading rate of 0.038 % per cycle and a capacity retention of 62%, in contrast to the fast capacity degradation of the Fe/NC and bare PP-based cell. The remarkable long-term cycling performance of the FeTe/NC can be ascribed to the efficient restriction of LiPSs toward the anode side (Fig. S32), thereby protecting the lithium metal from passivation (Fig. S33). The electrochemical performance of the FeTe/NC-modified separator is comparable to that of previously reported single-atom-based catalysts and even surpasses them in some cases (Fig. S34 and Table S7).

Furthermore, the effect of different separators on lithium plating/stripping behavior was evaluated using Li-Li symmetric cells. As shown in Fig. S35, under a current density of 1 mA cm^−2^ and a capacity of 1 mAh cm^−2^, the initial polarization voltages for cells with FeTe/NC, Fe/NC, and PP-based separators are 38, 52, and 54 mV, respectively. Notably, the PP-based cell exhibits an increase in overpotential to 85 mV after 500 h, indicating unstable Li deposition. In contrast, the FeTe/NC-based cell maintains a low overpotential (17 mV) for 500 h, evidencing stable solid electrolyte interphase (SEI) layer formation and uniform Li⁺ deposition. The FeTe/NC catalysts with different Te atom contents, namely FeTe/NC-0.75 and FeTe/NC-3, were also synthesized (Fig. S36), and their influence on battery performance was investigated. The optimized FeTe/NC catalyst demonstrates superior capacity and cycling stability (Fig. S37), while both FeTe/NC-0.75 and FeTe/NC-3 show diminished performance due to non-optimal Te loading - insufficient active sites in the former case and Te aggregation in the latter. XRD analyses of the FeTe/NC-modified separator before and after 100 cycles at 0.5 C confirm the stability of the FeTe/NC catalyst, as no new crystalline phases were detected, indicating the maintenance of its atomically dispersed feature. (Fig. S38) All these results demonstrate the superiority of FeTe/NC as an effective catalyst in Li-S chemistry. The remarkable rate and cycling performance could be attributed to the precise control over the coordination environment and unique electronic property of the central Fe active site, which could not only capture LiPSs in a more effective manner to mitigate the shuttling effect but also serve as a robust electrocatalytic mediator to promote the bidirectional LiPSs conversion.

### Density Functional Theory Calculations

**Fig. 6:**
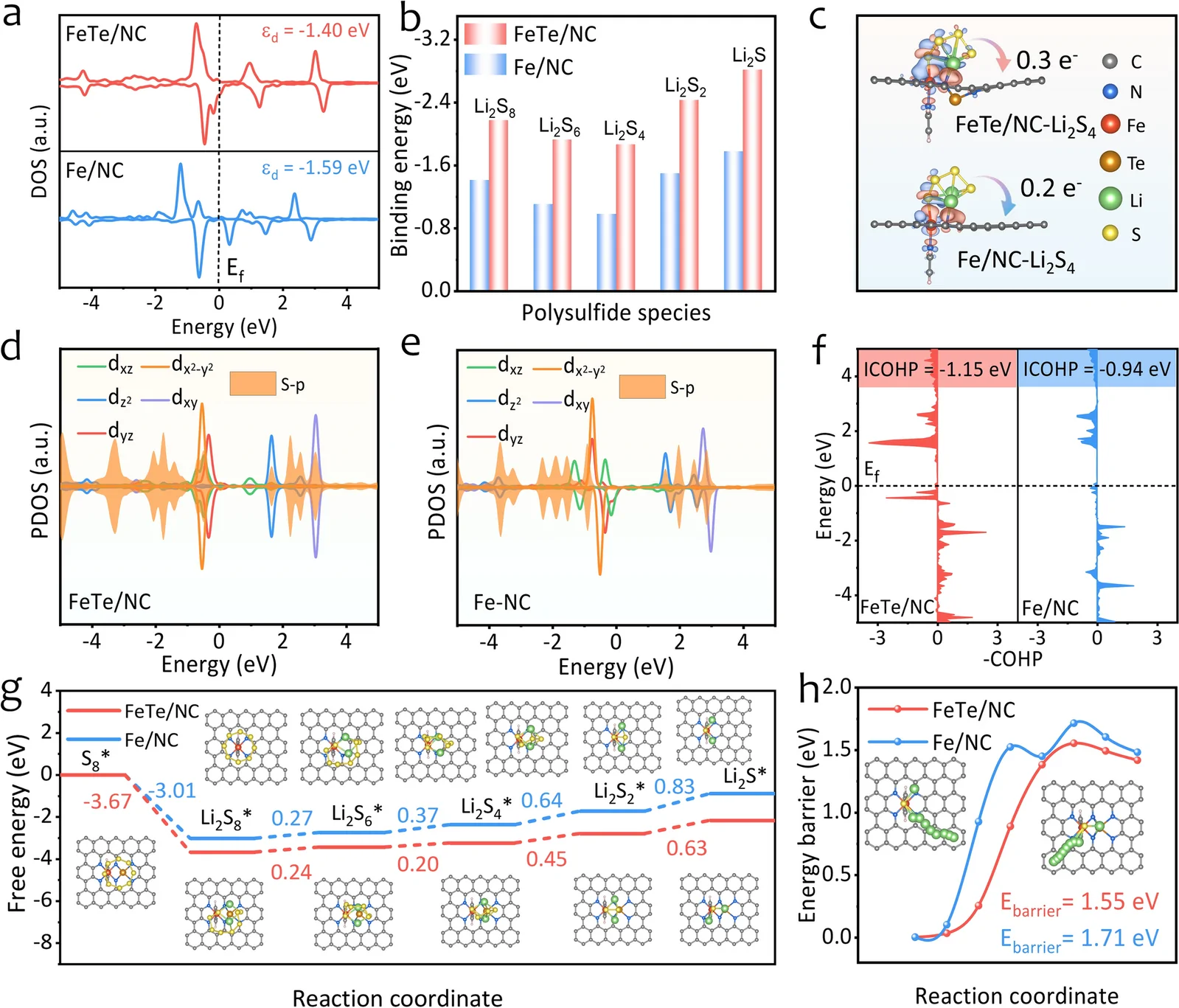
DFT calculations. **a** DOS of Fe 3d orbitals of FeTe/NC and Fe/NC. **b** Binding energies of polysulfide species on FeTe/NC and Fe/NC substrate. **c** Charge density difference of FeTe/NC and Fe/NC after Li_2_S_4_ adsorption, in which red and blue areas indicate electron accumulation and depletion, respectively. PDOS for the Li_2_S_4_ adsorbed on **d** FeTe/NC and **e** Fe/NC. **f** COHP of Fe-S for FeTe/NC and Fe/NC. **g** Free energy profiles for the conversion of sulfur species with optimized structures of the intermediates. **h** Energy profiles for the decomposition of Li_2_S on FeTe/NC and Fe/NC

The exceptional ability of FeTe/NC to capture sulfur species and accelerate redox reactions could be attributed to its unique electronic structure and strong interactions with sulfur species [45]. To elucidate the underlying mechanism, density functional theory (DFT) calculations were further performed at the atomic-level. The optimized configurations of FeTe/NC with the coordinated FeN_5_-TeN_4_ moiety and Fe/NC with the FeN_5_ moiety are illustrated in Fig. S39. The charge density difference and Bader charge analysis indicate notable electronic redistribution around the Fe central atom, where the electrons transferred from Te to Fe atom in the FeTe/NC catalyst (Fig. S40 and Table S8). Density of states (DOS) calculations are performed to further elucidate the orbital regulation of the central Fe atom. As shown in Fig. 6a, the DOS of FeTe/NC displays a higher occupation near the Fermi level compared to that of Fe/NC, indicating improved conductivity and faster electron transfer [46]. Notably, FeTe/NC shows a higher *d*-band center (*ε*_d_) of  −1.40 eV and energy levels of the d_xz/yz_ orbitals than that of Fe/NC ( −1.59 eV) (Fig. S41), the lifted ε_d_ and antibonding orbital energy level would reduce electronic occupancy in the antibonding orbital, thereby strengthening the adsorption toward LiPSs and enhancing the catalytic activity [26].

Given this, the adsorption of a series of sulfur species on FeTe/NC and Fe/NC was studied (Fig. 6b) and the corresponding adsorption configurations are depicted in Figs. S42 and S43, demonstrating Lewis acid–base interaction of Fe and S, as well as electrostatic adsorption of Li and N [47]. Evidently, the FeTe/NC delivers stronger adsorption capability toward sulfur species, which accords with its higher ε_d_ and sulfur affinity. The charge transfer upon the interaction was studied by the differential charge density in Figs. 6c and S44, where Li_2_S_4_ was employed as a representative LiPSs. Strong charge accumulation can be noticed between metal and sulfur and much more charge transfer can be observed in the FeTe/NC scenario compared with the Fe/NC counterpart (0.3 versus 0.2 e), elucidating the significantly intensified host-guest interactions by the Te modulating. The projected density of states (PDOS) of Li_2_S_4_ adsorbed on FeTe/NC also confirms that FeTe/NC with reduced electron occupation in antibonding states strengthens the *d-p* hybridization between Fe 3*d* orbital and S 2*p* orbital, in which there are more overlaps between the p orbitals of S atoms in sulfur species and the d orbitals of Fe atoms in FeTe/NC compared with Fe/NC (Fig. 6d, e), leading to strong sulfur affinity and facilitate the sulfur redox kinetics. Crystal orbital Hamiltonian population (COHP) of Fe-S bonds further quantifies the strength and bonding–antibonding properties upon the host–guest interaction, where the values of -COHP signify the bonding/antibonding character, as well as the bonding strength between Fe and S. As shown in Fig. 6f, Li_2_S_4_ adsorption on FeTe/NC shows more bonding states (positive range) and less antibonding states compared to that of Fe/NC, revealing a more negative integrated COHP (ICOHP) value of  −1.15 eV compared to Fe/NC ( −0.94 eV), certifying the stronger Fe-S bonding and enhanced p-d orbital interaction in the Te-tuned scenario.

On this basis, the Gibbs free energy profiles for sulfur reduction on different catalytic surfaces are presented in Fig. 6g. The conversion step from Li_2_S_2_ to Li_2_S presents the highest energy barrier on both FeTe/NC and Fe/NC surfaces, identifying it as the rate-limiting step during the discharge process. By comparison, the ∆*G* value at the decisive step for the FeTe/NC (0.63 eV) is lower than that for the Fe/NC (0.83 eV) catalyst, indicating enhanced thermodynamic favorability for sulfur reduction on FeTe/NC. The reverse Li_2_S decomposition behavior was also investigated (Fig. 6h). A relatively low energy barrier for Li_2_S decomposition on FeTe/NC (1.55 eV) highlights its key role in speeding up delithiation reaction kinetics. Moreover, the lithium-ion diffusion behaviors on the two substrates were investigated. Figure S45 reveals that FeTe/NC possesses a lower lithium-ion diffusion barrier, enabling faster lithium-ion transport and thereby promoting the conversion of sulfur species during the charge/discharge process. Collectively, these results verify the feasibility of employing Te species to modulate the electronic structure of Fe-based SAC, leading to enhanced adsorption of sulfur species and improved conversion kinetics.

## Conclusions

In summary, the Te-modulated Fe single-atom catalyst with FeN_5_-TeN_4_ moieties has been delicately designed and successfully fabricated in the pursuit of boosting bidirectional polysulfide electrocatalysis. The introduction of Te atoms regulates the local coordination configuration and the electronic structure of the central Fe site by triggering a polarized charge distribution. This modulation brings the d-band center closer to the Fermi level, reducing the electronic occupation in the antibonding states and strengthening the d-p orbital hybridization between the catalyst and sulfur species, thereby synergistically optimizing the adsorption behavior towards LiPSs and facilitating the bidirectional redox process of Li-S batteries. Benefiting from the synergistic interactions among Fe, Te, N, and C, the FeTe/NC mediator exhibits favorable electrocatalytic properties that significantly enhance its electrochemical performance. The batteries with FeTe/NC-modified separators manifest remarkable rate performance with 735 mAh g^−1^ at 5 C, extraordinary cycling stability featuring a capacity fading rate of only 0.038% per cycle after 1000 cycles at 1 C, and anticipated areal capacity of up to 5.6 mAh cm^−2^ with a high sulfur loading of 8.7 mg cm^−2^, effectively confirming the synergistic catalytic effect for the entire sulfur conversion process. This work offers an insightful and effective strategy for designing and tuning the electronic structure of SACs in the development of advanced Li-S batteries.

## Supplementary Information

Below is the link to the electronic supplementary material.

Supplementary file1 (DOCX 14558 KB)
