# Supplementary material for: Te-Modulated Fe Single Atom with Synergistic Bidirectional Catalysis for High-Rate and Long–Cycling Lithium-Sulfur Battery
Source: Nanomicro Lett. 2025 Aug 11;18:31. doi: 10.1007/s40820-025-01873-3 (PMC12339806; doi:10.1007/s40820-025-01873-3)
Supplement: Supplementary file 1 — Supplementary file1 (DOCX 14558 KB) [file 40820_2025_1873_MOESM1_ESM.docx]

Supporting Information for

**Te-Modulated Fe Single-Atom with Synergistic Bidirectional Catalysis for High-Rate and Long-Cycling Lithium-Sulfur Battery**

Jian Guo^1, #^, Lu Chen^2, #^, Lijun Wang^1^, Kangfei Liu^3^, Ting He^2,^ *, Jia Yu^4,^ *, and Hongbin Zhao^1,^ *

^1^Department of Chemistry, College of Sciences, Shanghai University, Shanghai 200444, P. R. China

^2^School of Chemical Science and Engineering, Tongji University, Shanghai 200092, P. R. China

^3^School of Chemical Engineering, East China University of Science and Technology, Shanghai 200237, P. R. China

^4^Key Laboratory of Precision and Intelligent Chemistry, University of Science and Technology of China, Hefei 230026, P. R. China

^#^Jian Guo and Lu Chen contributed equally to this work.

*Corresponding authors. E-mail: heting@tongji.edu.cn (Ting He); jiayu@ustc.edu.cn (Jia Yu); hongbinzhao@shu.edu.cn (Hongbin Zhao)

**S1 Supplementary Experimental Section**

**S1.1 Preparation of Modified Separators**

The FeTe/NC and Fe/NC modified separators were prepared using a simple surface coating method. In short, FeTe/NC or Fe/NC, Ketjen black (KB), and polyvinylidene fluoride (PVDF) binder were mixed using N-methyl pyrrolidone (NMP) as a solvent to form a slurry in a weight ratio of 8:1:1. Then, the mixture was coated on commercial Celgard 2400 polypropylene (PP) separator and further dried at 60 °C under vacuum to evaporate the solvent. Finally, the functionalized separator was cut into discs with a diameter of 19 mm and the areal mass loading weight of coating materials was about 0.5 mg cm^-2^.

**S1.2 Preparation of CNT/S Composite**

CNT/S composite was synthesized by a typical melt-diffusion method. In brief, the CNT powder was continuously ground with sublimed sulfur (3:7 in W/W) and then heated in a sealed vial at 155 °C for 12 h.

**S1.3 Electrochemical Measurements**

The CNT/S, KB, and PVDF were mixed with a mass ratio of 8:1:1 and put into an NMP solution to prepare a slurry. The slurry was cast on the aluminum foil and then vacuum dried at 60 °C for 12 h to serve as a cathode. The sulfur loading on each electrode was weighed to be ∼1.2 mg cm^-2^. The aqueous PTFE solution was used to prepare the cathodes of high-sulfur-loading (8.7 mg cm^-2^). The assembled CR2016-type coin cells employed Li foil as the anode, FeTe/NC, Fe/NC modified separators and bare PP as the separator. 1 M LiTFSI in 1,3-dioxolane (DOL)/1,2-dimethoxyethane (DME) (1:1 by volume) with 2.0 wt.% LiNO_3_ was used as the electrolyte. The ratio of the electrolyte and sulfur (E/S) was demanded at 15 μL mg^-1^ for regular batteries. Given high-loading cathodes, the E/S ratio was 4.9 μL mg^-1^. The electrochemical impedance spectroscopy (EIS) and cyclic voltammetry (CV) experiments were recorded by the CHI760E workstation. Galvanostatic charge-discharge (GCD) tests were conducted on a Land battery test system (Land CT2001, Wuhan) at 25 °C in a potential range between 1.7 and 2.8 V.

***S1.3.1 Symmetrical Cell Assembly and Measurements***

A 9:1 weight ratio of active materials and PVDF was mixed in NMP solution and the paste was coated on aluminum foil to prepare the electrodes. 0.2 M Li_2_S_6_ solution as electrolyte and PP as separators. To estimate the electrochemical properties, the CHI760E workstation was employed to test the CV and EIS measurements.

***S1.3.2 Li_2_S Nucleation and*** ***Dissolution Tests***

For the liquid-solid conversion kinetics, S and Li_2_S (7:1 molar ratio) were mixed in a DOL/DME (v/v=1:1) solution with 1.0 M LiTFSI and 2.0 wt.% LiNO_3_, and a 0.2 M Li_2_S_8_ solution was formed after intense stirring. The active materials were coated on aluminum foil as the working electrode. Lithium metal was used as the counter electrode and PP as the separator. The Li_2_S_8_ solution was dripped on the working electrode, and the 1 M LiTFSI electrolyte was added on the other side. The cells were discharged to 2.11 V at 0.114 mA and then kept at 2.09 V for Li_2_S to nucleate until the current dropped below 10^-5^ A. To analyze the Li_2_S dissolution, fresh cells were galvanostatically discharged under a current of 0.114 mA until the voltage was reduced to 1.7 V; then, the cells were potentiostatically charged at 2.40 V.

***S1.3.3 Theoretical Equations for Current-time Transients of Four Classical Electrochemical Deposition Models*** [S1]

$2DI:\frac{j}{j_{m}}=\left( \frac{t}{t_{m}} \right)\left\{ \exp\left[ \frac{t^{2}-{t_{m}}^{2}}{2{t_{m}}^{2}} \right] \right\}$ (S1)

$2DP:\frac{j}{j_{m}}={(\frac{t}{t_{m}})}^{2}\{exp[\frac{-2(t^{3}-{t_{m}}^{3})}{3{t_{m}}^{3}}]\}$ (S2)

$3DI:\frac{j}{j_{m}}={(\frac{1.9542}{t/{t_{m}}})}^{1/2}\{1-exp[1.2564(\frac{t}{t_{m}})]\}$ (S3)

$3DP:\frac{j}{j_{m}}={(\frac{1.2254}{t/{t_{m}}})}^{1/2}\{1-exp[2.3367{(\frac{t}{t_{m}})}^{2}]\}$ (S4)

***S1.3.4 Calculation of Difference in Activation Energy*** [S2]

$E_{a}=E_{a}^{0}-RT/b{}_{cathode}$ (S5)

where $E_{a}^{0}$ is the intrinsic activation energy, $E_{a}$ is the activation energy of discharge and charge process, ${}_{cathode}$ is the irreversible potential during CV test, *b* is the slope of the Tafel plot.

***S1.3.5 Calculation of Li-ion Diffusion Constant***

The Li-ion diffusion constant can be estimated using the Randles-Sevcik equation [S3]:

$I_{p}=2.69\times{10}^{5}n^{3/2}AD_{Li}^{1/2}v^{1/2}C_{Li}$ (S6)

where $I_{p}$ is the peak current, n represents electron number, A represents electrode area, $D_{Li}$ is the Li-ion diffusion coefficient, $C_{Li}$ is the Li-ion concentration in the electrochemical reaction and v is the scanning rate.

***S1.3.6 Calculation of Internal Resistance***

The internal resistance (∆R_internal_) was derived from the following equation [S4]:

$\Delta R_{internal}\left( \Omega\right)={\Delta_{QOCV-OCV}}/{I_{appilied}}$ (S7)

where ∆V denotes the voltage difference between QOCV and OCV, while $I_{applied}$ refers to the applied current, and $\Delta R_{internal}$ represents the internal resistance of the batteries, normalized overtime during the lithiation/delithiation process.

**S1.4 Computational Methods**

All calculations were performed in the framework of the spin-polarized density functional theory (DFT) within the Projected Augmented Wave (PAW) method implemented in the Vienna ab initio simulation package (VASP) [S5-S7]. The Perdew-Burke-Ernzerhof (PBE) function was adopted to describe electron exchange and correlation energy as it has been proven to provide a useful trend in calculating the adsorption energy [S8, S9]. The cut-off energy for plane-wave basis was set as 450 eV to ensure the precision of calculations and the total energy convergence criterion for energy is 10^-5^ eV, with the force convergence thresholds set at 0.03 eV/Å for geometric optimization. 3×3×1 Monkhorst-Pack grids were used for geometric optimization on all the models. A vacuum layer of at least 15 Å was applied in the z-direction of the slab model to prevent vertical interaction between the slabs. The D3 method of Grimme was employed to describe the van der Waals (vdW) dispersion for calculation [S10]. The binding energy for polysulfide was calculated using the equation:

$\Delta E=E_{surf+ads}-(E_{surf}+E_{ads})$ (S8)

where $E_{sur+ads}$ is the energy of the absorbed system, $E_{ads}$ is the energy of isolated polysulfides and $E_{surf}$ is the energy of the corresponding surface. The CI-NEB was applied for the calculations of Li_2_S decomposition and lithium-ion migration [S11].

**S1.5 Materials Characterization**

The morphologies of the materials were recorded by using a scanning electron microscope (SEM, Pharos G2, accelerating voltage = 15 kV), high-resolution transmission electron microscopy (HRTEM, JEOL JEM-2100, accelerating voltage = 200 kV) and aberration-corrected high-angle annular darkfield scanning transmission electron microscope (AC-HAADF-STEM, JEOL JEM-ARM200F, operated at 200 kV). The crystalline structures of all the samples were identified by using Bruker D8 advance (Cu Kα source, λ = 1.54056 Å). X-ray photoelectron spectroscopy (XPS) was carried out on Thermo Scientific K-ALPHA. The X-ray absorption fine structure (XAFS) analysis was carried out on beamline 1W1B at the Beijing Synchrotron Radiation Facility (BSRF, Beijing, China).

**S2 Supplementary Figures and Tables**


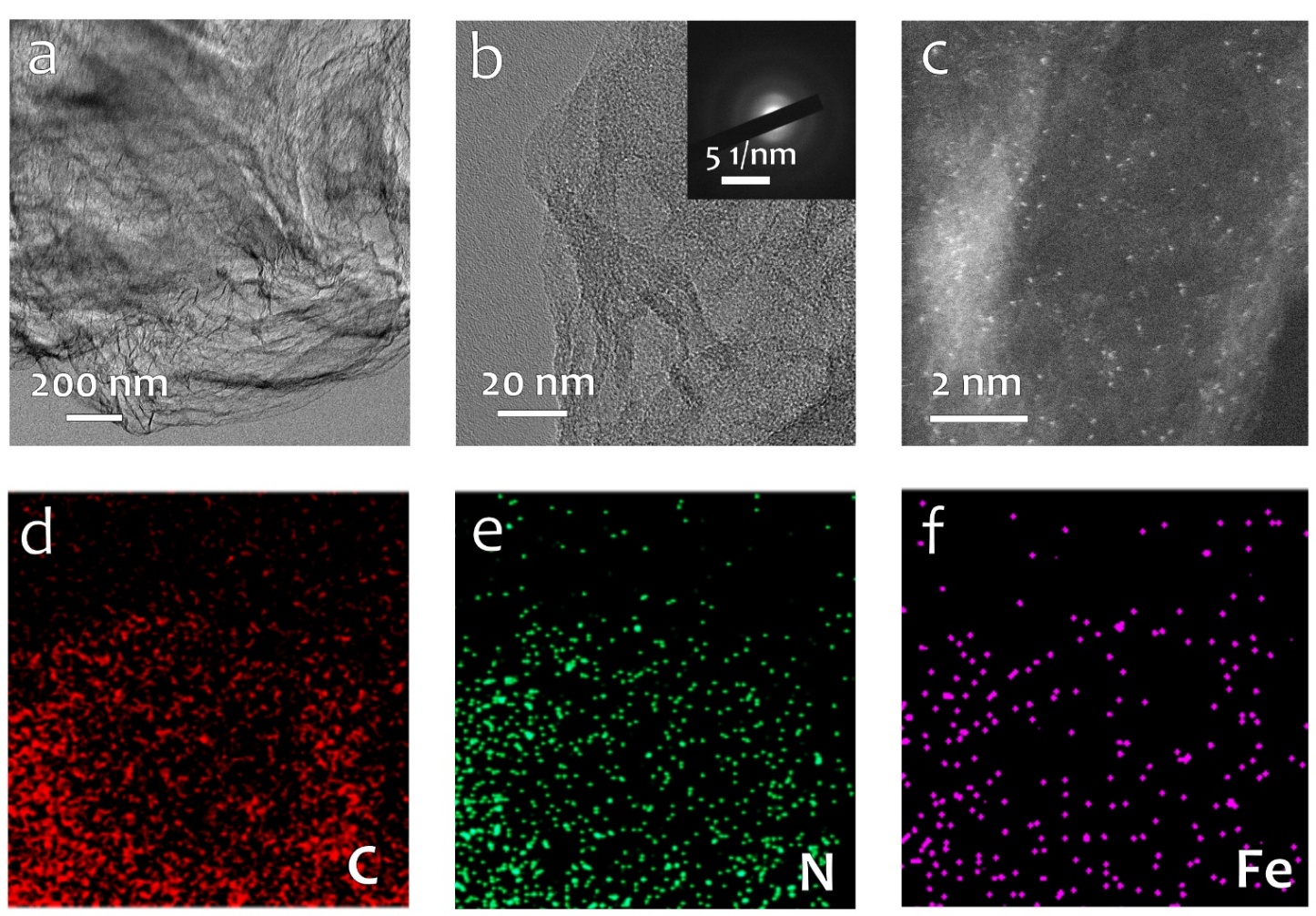


**Fig. S1** (**a**) TEM, (**b**) HRTEM (inset: SAED pattern), and (**c**) AC-HAADF-STEM image and (d-f) elemental mappings of Fe/NC


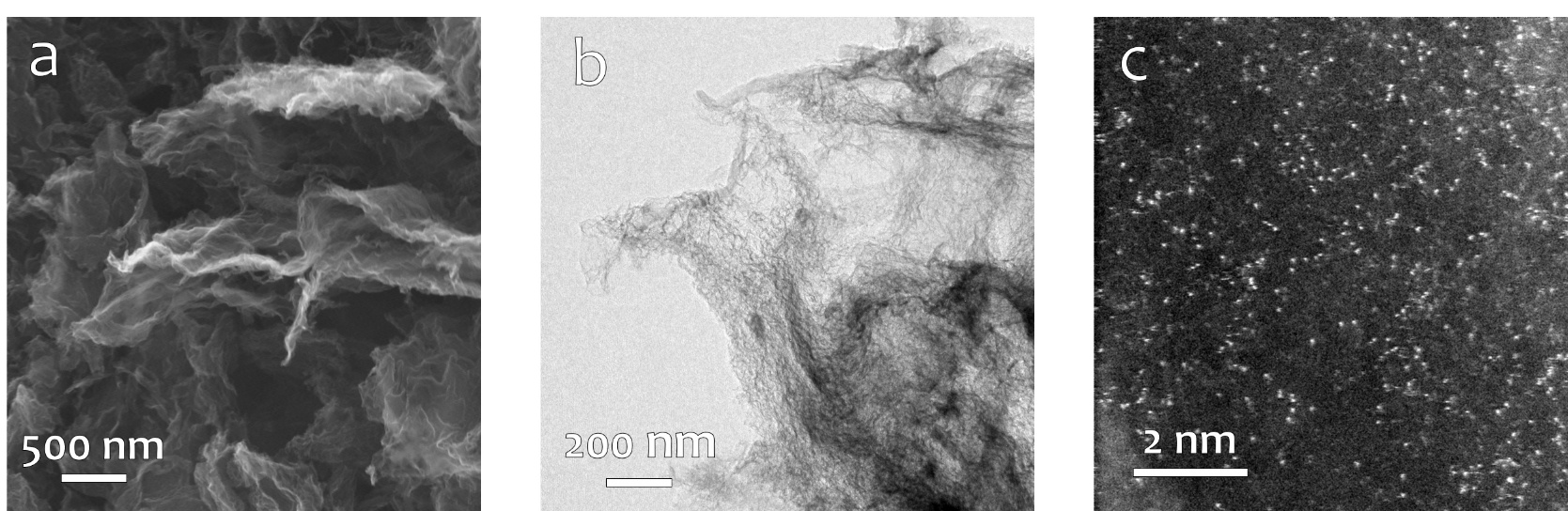


**Fig. S2** (**a**) SEM, (**b**) TEM, and (**c**) aberration-corrected STEM image of Te/NC


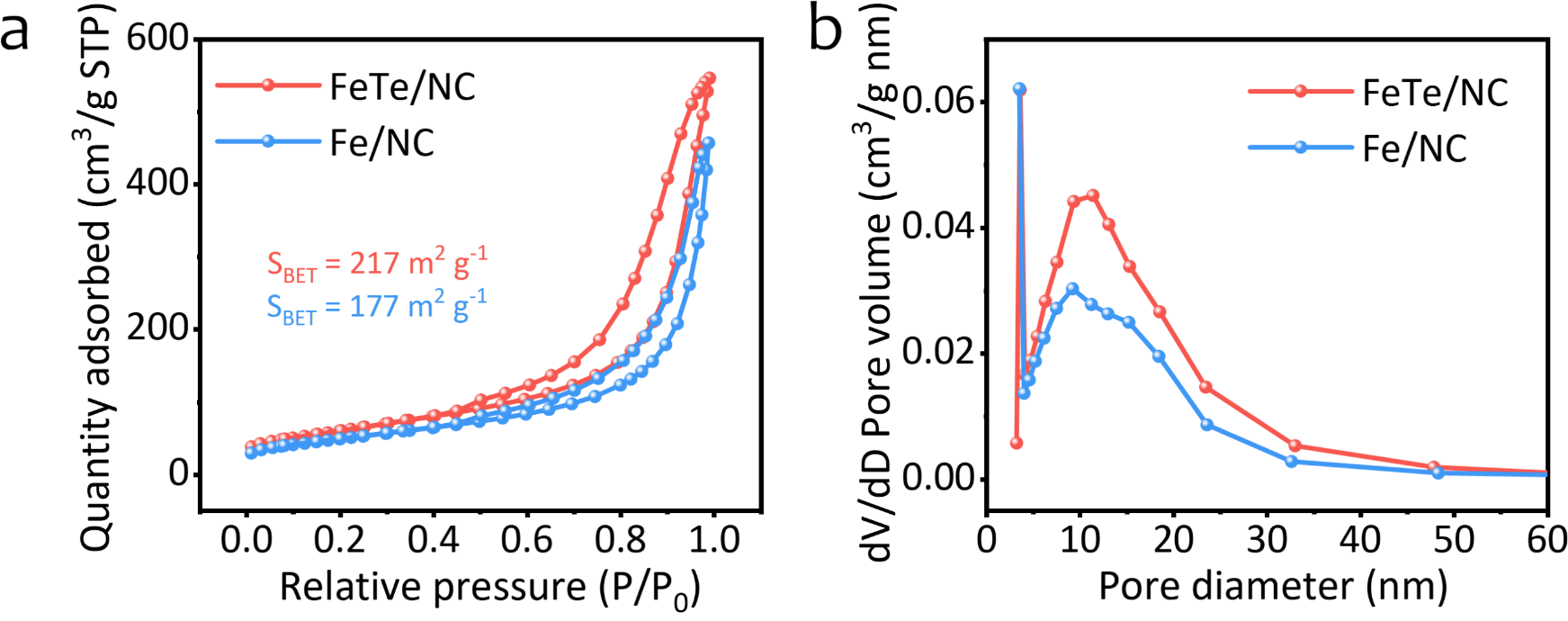


**Fig. S3** (a) N_2_ adsorption/desorption isotherms and (b) the corresponding pore size distributions of FeTe/NC and Fe/NC


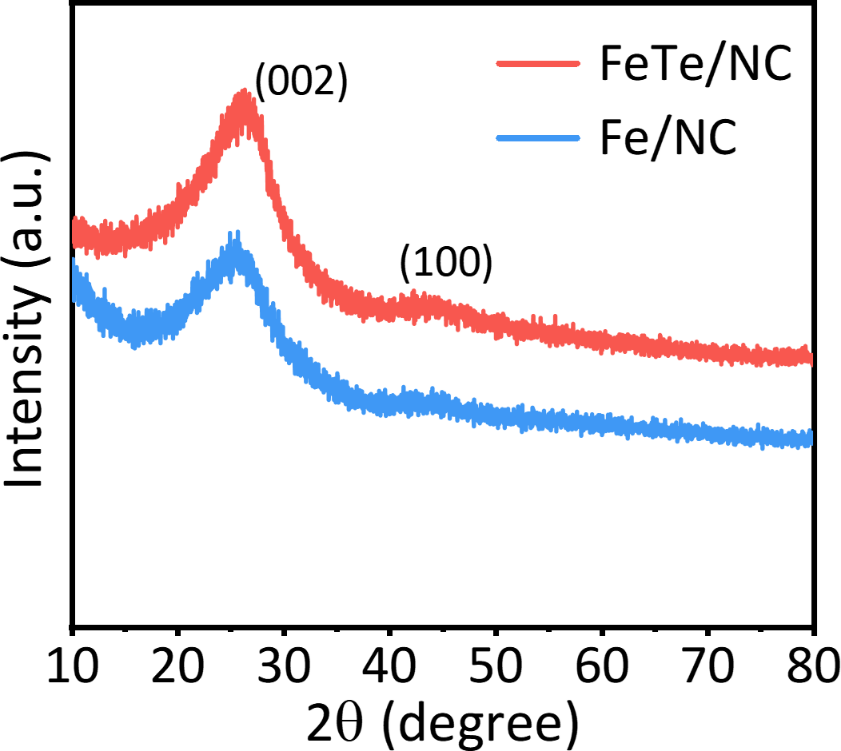


**Fig. S4** XRD patterns of FeTe/NC and Fe/NC


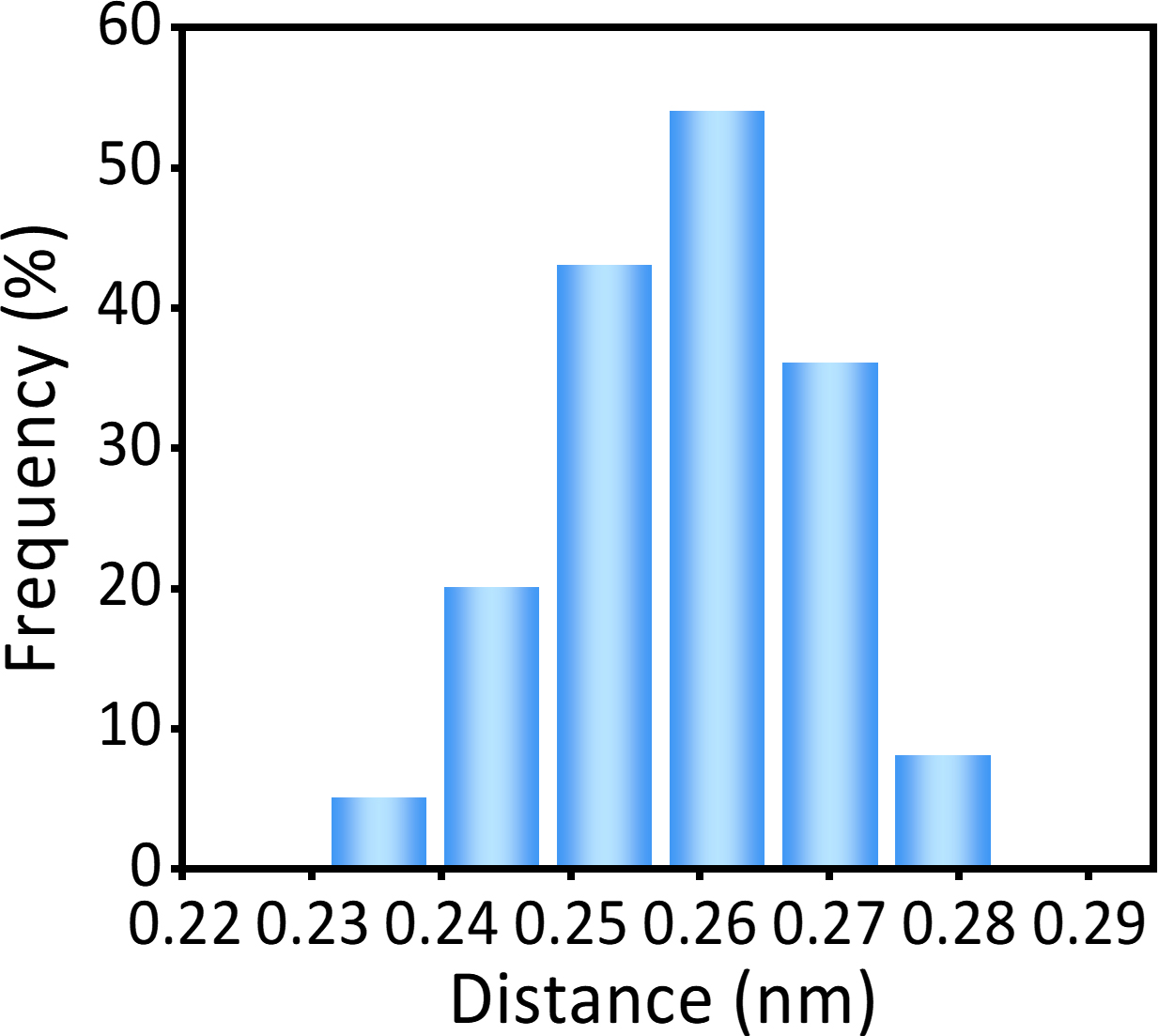


**Fig. S5** Statistics on the interatomic distances of the Fe-Te diatomic pairs in the AC-STEM image


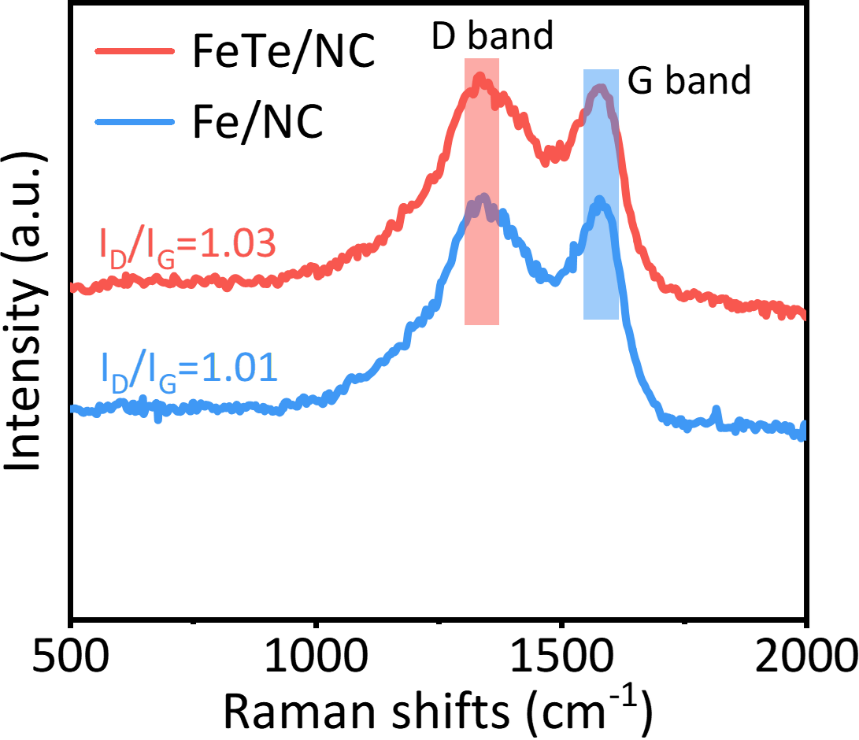


**Fig. S6** Raman spectra of FeTe/NC and Fe/NC


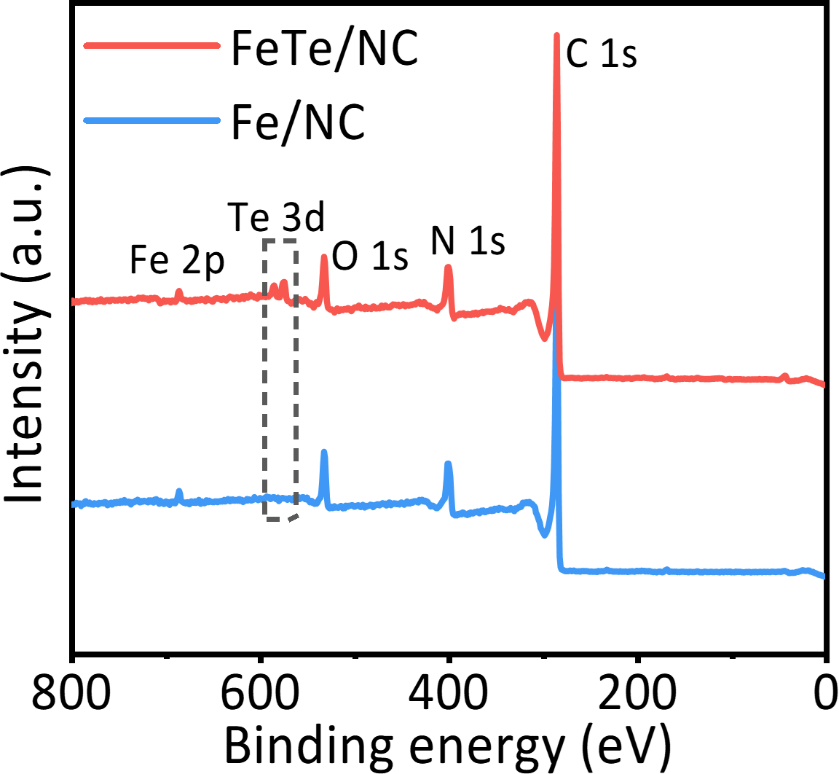


**Fig. S7** XPS surveys of FeTe/NC and Fe/NC


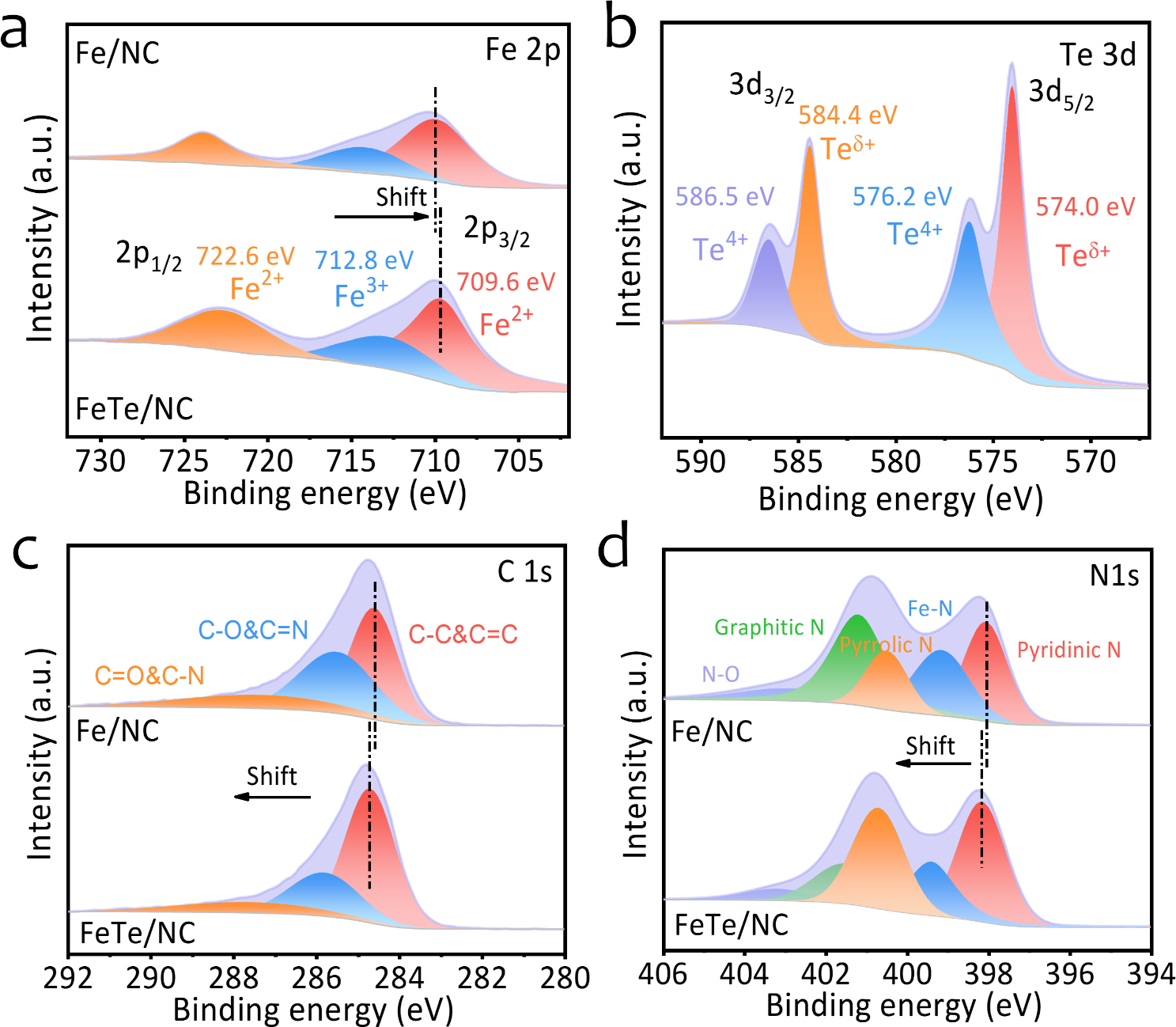


**Fig. S8** XPS spectra of (**a**) Fe 2p, (**b**) Te 3d, (**c**) C 1s and (**d**) N 1s

**Note:** After the incorporation of Te atoms, the binding energy of C 1s and N 1s shifts to higher energy, which is probably attributed to the Te atom promoting electron transfer from C or N to Te [S12].


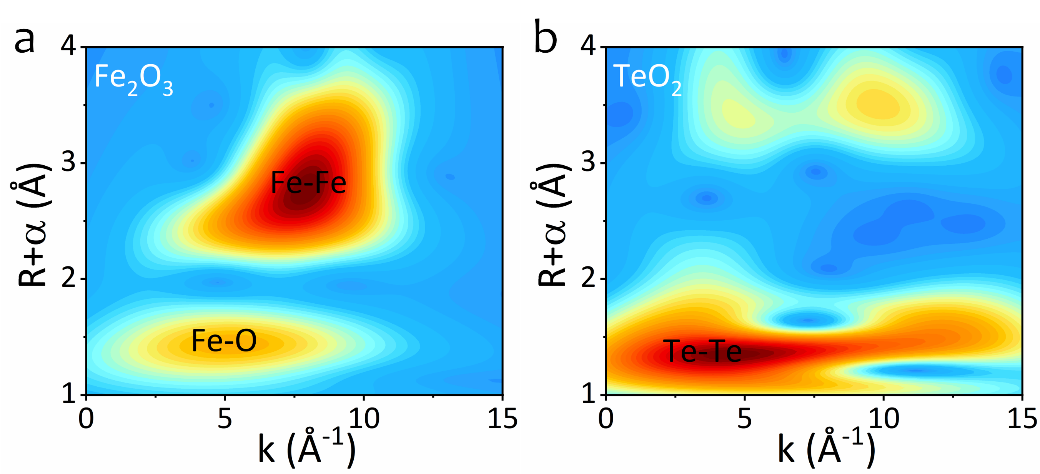


**Fig. S9** WT k^3^-weighted EXAFS spectra of (**a**) Fe_2_O_3_ and (**b**) TeO_2_


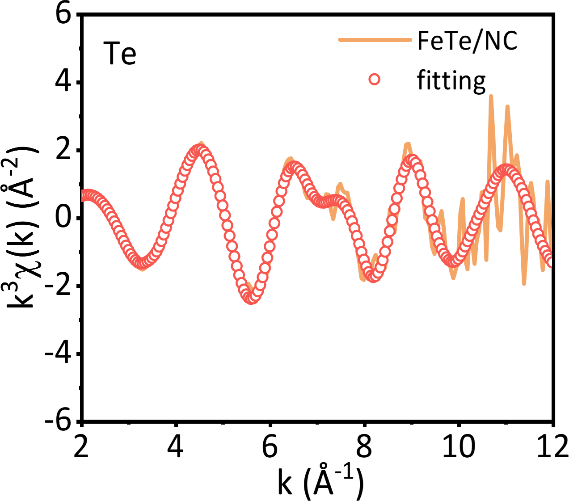


**Fig. S10** FT-EXAFS fitting curve in k space of FeTe/NC at the Te K-edge


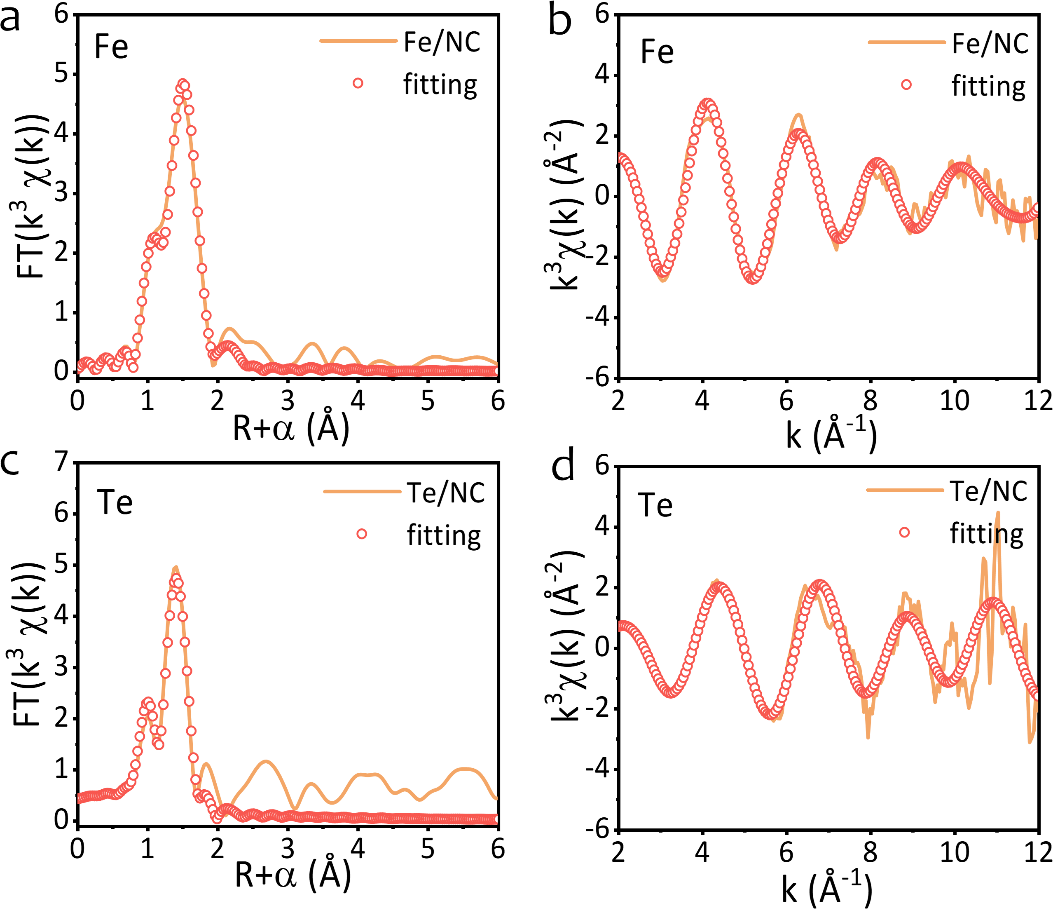


**Fig. S11** Fe K-edge FT-EXAFS analysis of Fe/NC (**a**) in R space and (**b**) in k space. Te K-edge FT-EXAFS analysis of Te/NC (**c**) in R space and (d) in k space

**Note:** The fitting outcomes revealed that, in Fe/NC, the coordination pattern between the Fe and N atoms was Fe-N_5_. The fitting results for Te/NC indicate that the Te-N coordination number is 3, forming a Te-N_3_ coordination structure.


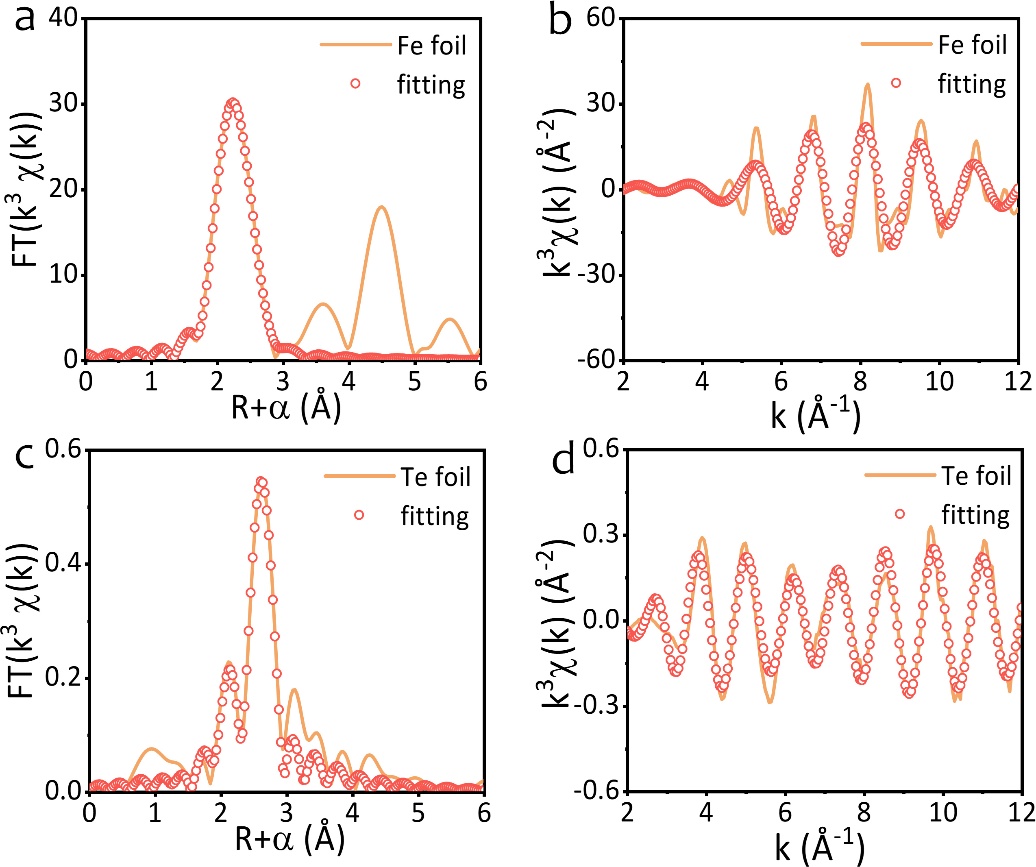


**Fig. S12** Fe K-edge FT-EXAFS analysis in R space (**a**) and (**b**) k space. Te K-edge FT-EXAFS analysis in R space (**c**) and (**d**) k space


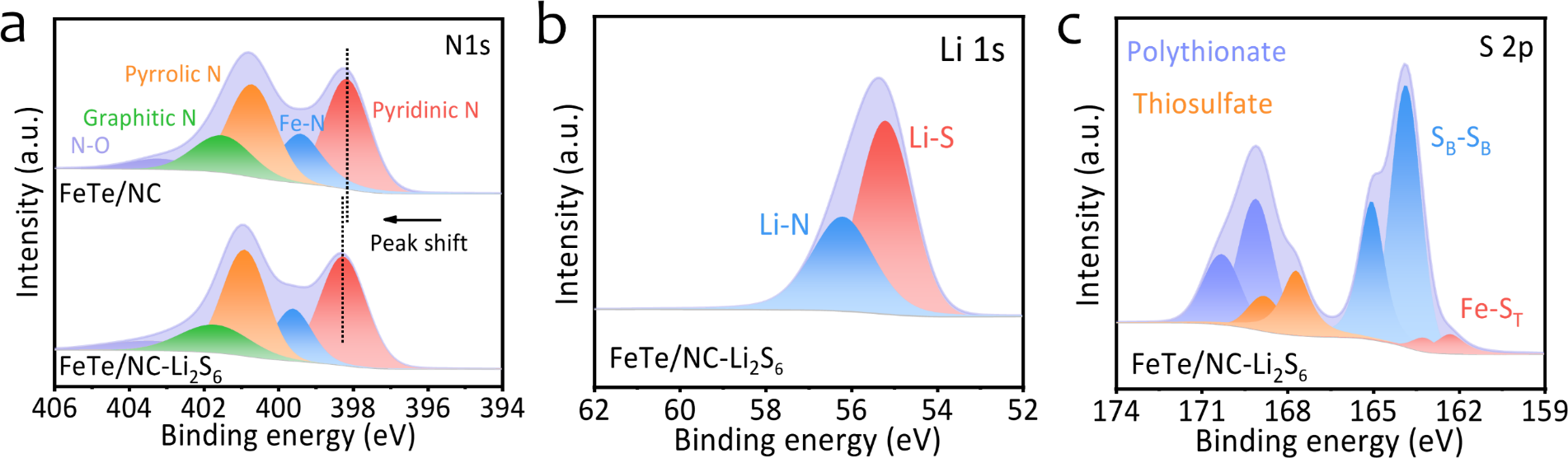


**Fig. S13** XPS spectra of (**a**) N 1s, (**b**) Li 1s, and (**c**) S 2p

**Note:** In the N 1s spectrum of FeTe/NC-Li_2_S_6_ (Fig. S13a), the peak shifts to a higher binding energy. A distinct peak at 56.2 eV can be observed in the Li 1s spectrum (Fig. S13b), ascribed to Li-N bonding between polysulfides and nitrogen in FeTe/NC, indicating strong electrostatic dipole interactions between Li^+^ and electronegative N atoms. As shown in Fig. S13c, The S 2p spectrum following the adsorption test displays four distinct contributions at 162.3/163.2, 163.9/165.1, 167.7/168.8, and 169.1/170.3 eV, corresponding to the terminal sulfur (S_T_^-1^), bridged sulfur (S_B_^0^), and oxidized sulfur species (such as polythionate and thiosulfate), respectively. The observation of an additional peak associated with the Fe-S bond suggests that the active Fe sites facilitate the capture and anchoring of LiPSs [S13].


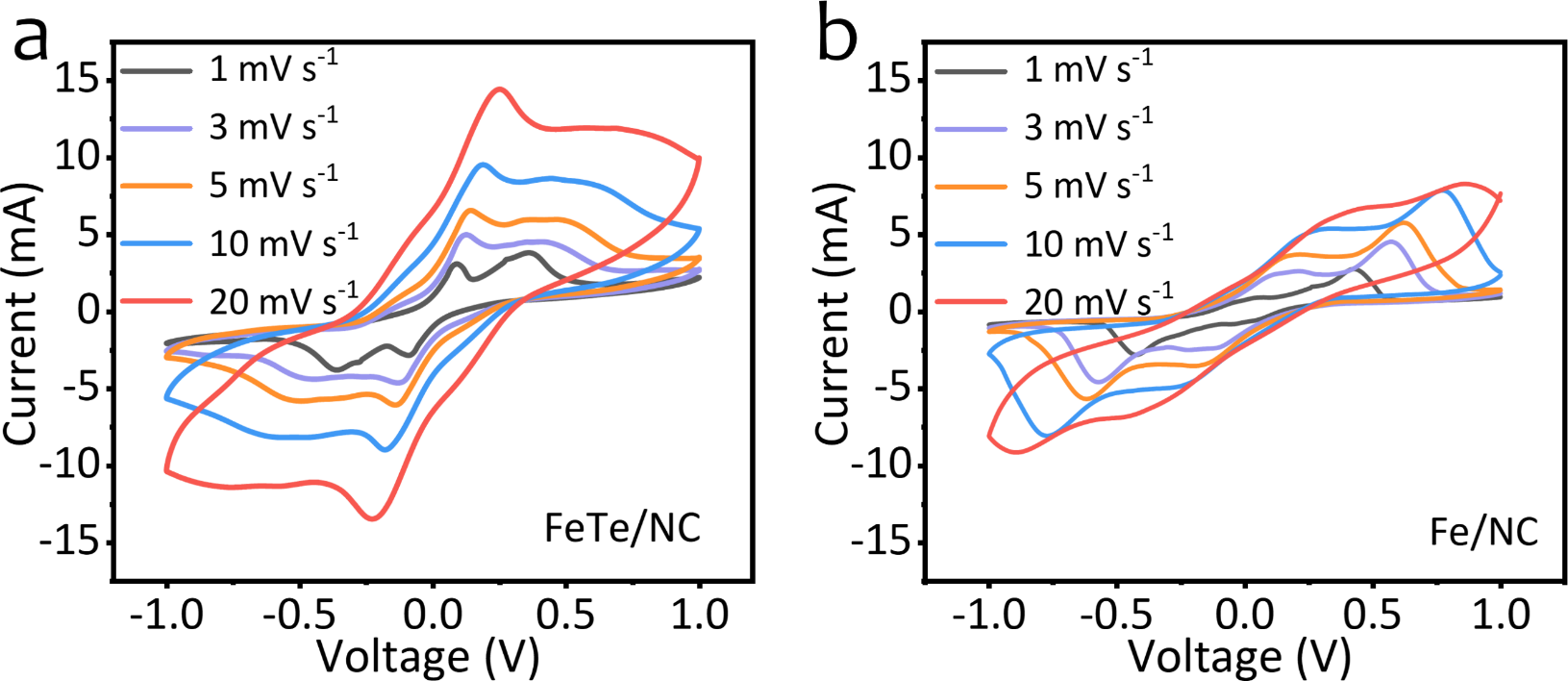


**Fig. S14** CV curves of the Li_2_S_6_ symmetric cells with FeTe/NC and Fe/NC electrodes


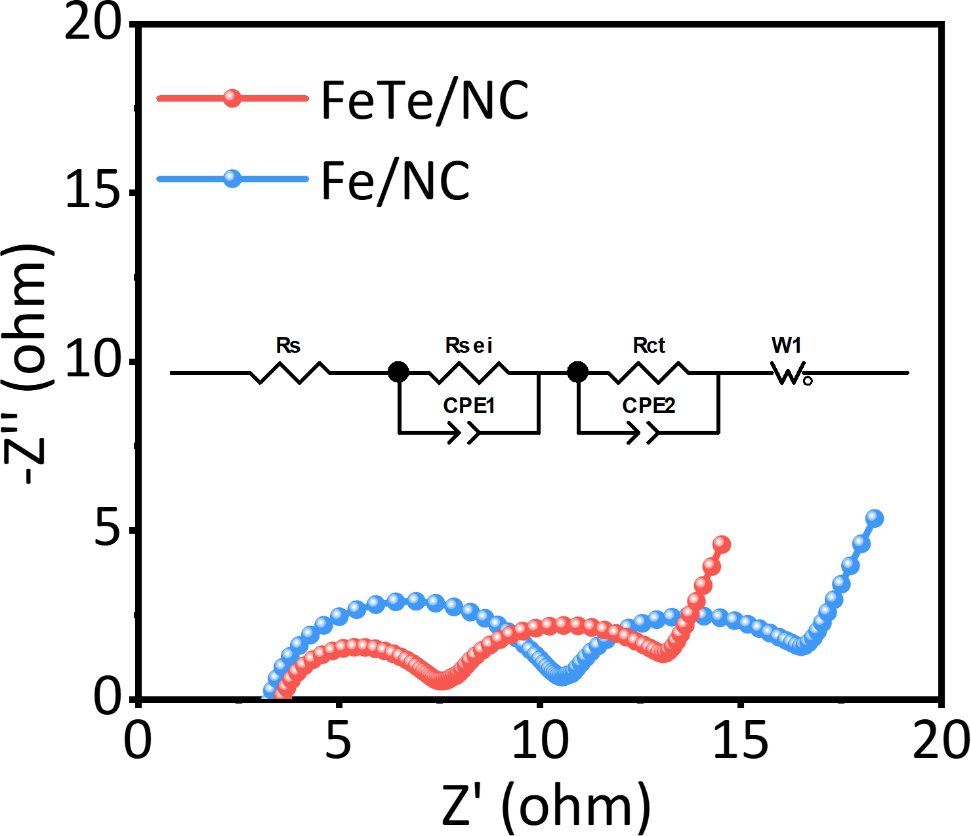


**Fig. S15** EIS of the symmetric cells with FeTe/NC and Fe/NC electrodes

**Note:** Rs: The internal resistance of the electrolyte; Rsei: The internal resistance of the solid electrolyte interface (SEI) film correlated with insoluble Li_2_S_2_/Li_2_S; Rct: The charge-transfer resistance related to the electrode reaction kinetics; CPE1: Capacitance of the electrode bulk in the high-frequency region; CPE2: Capacitance of the charge transfer process at the interface between sulfur and electrolyte; W1: The semi-infinite Warburg diffusion impedance of long-chain LiPSs.


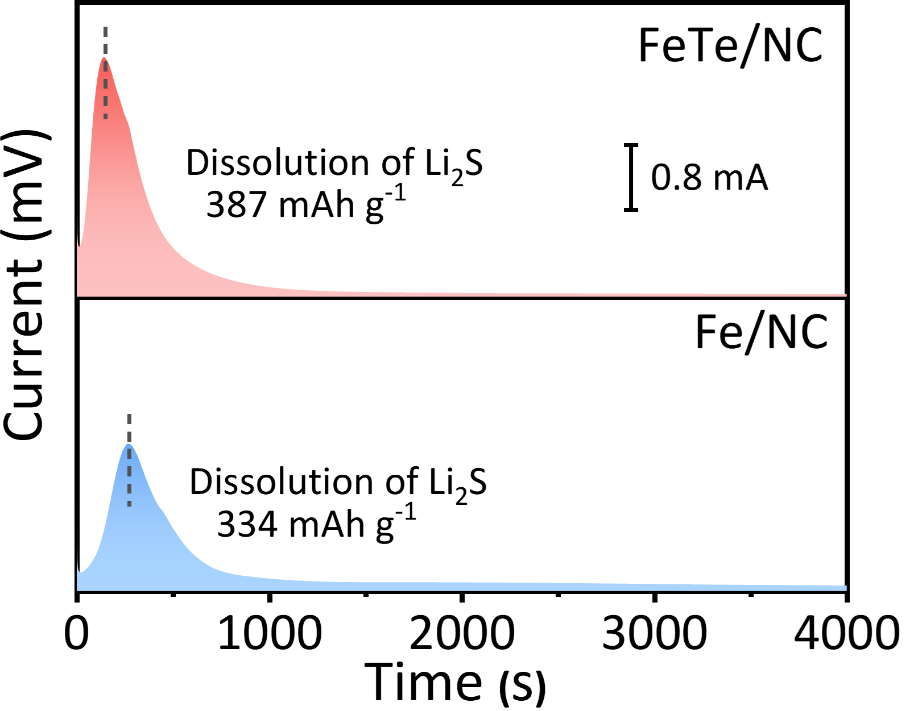


**Fig. S16** Potentiostatic charge profiles at 2.4 V for FeTe/NC and Fe/NC electrodes


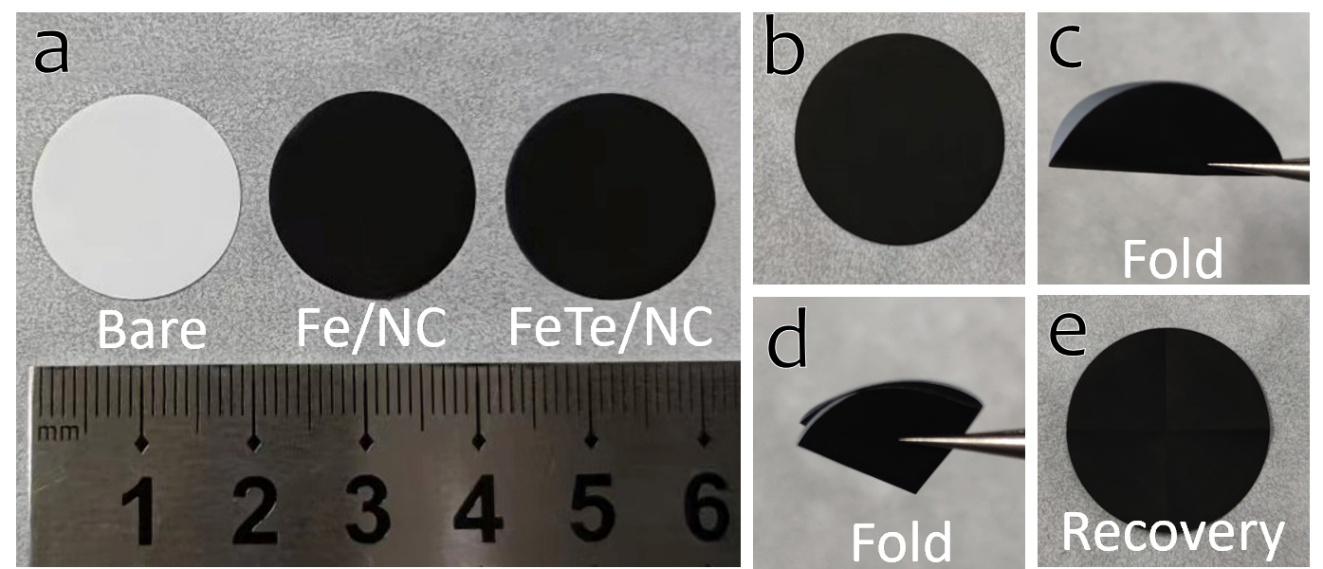


**Fig. S17** (**a**) Photographs of the bare PP, Fe/NC, and FeTe/NC modified separators. (**b-e**) FeTe/NC modified separator at bending state and after recovery

**Note:** The prepared separators show robustness; no appreciable delamination and deformation can be detected after the folding or bending test.


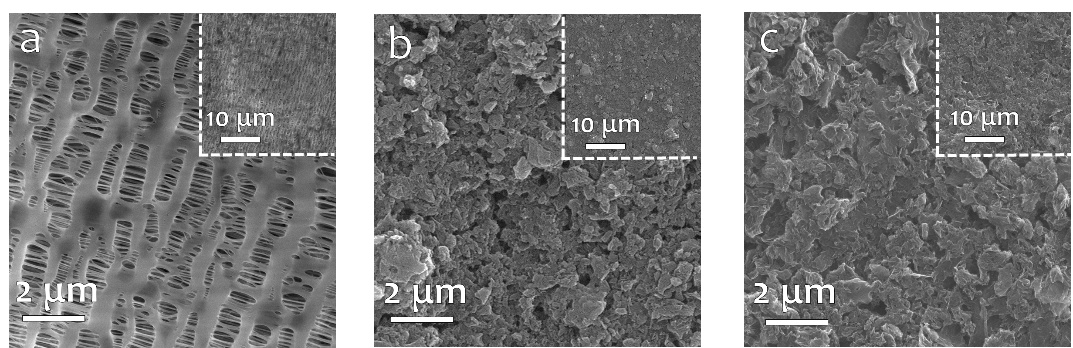


**Fig. S18** SEM images of one side of the (a) bare PP, (b) Fe/NC and (c) FeTe/NC modified separators


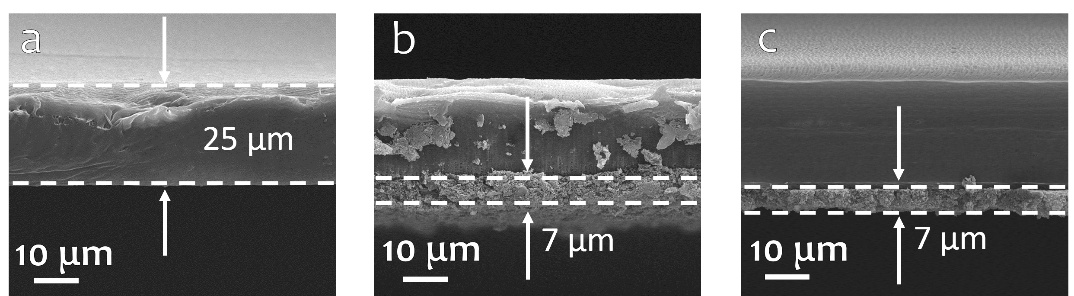


**Fig. S19** Cross-sectional SEM images of the (a) bare PP, (b) Fe/NC, and (c) FeTe/NC modified separators


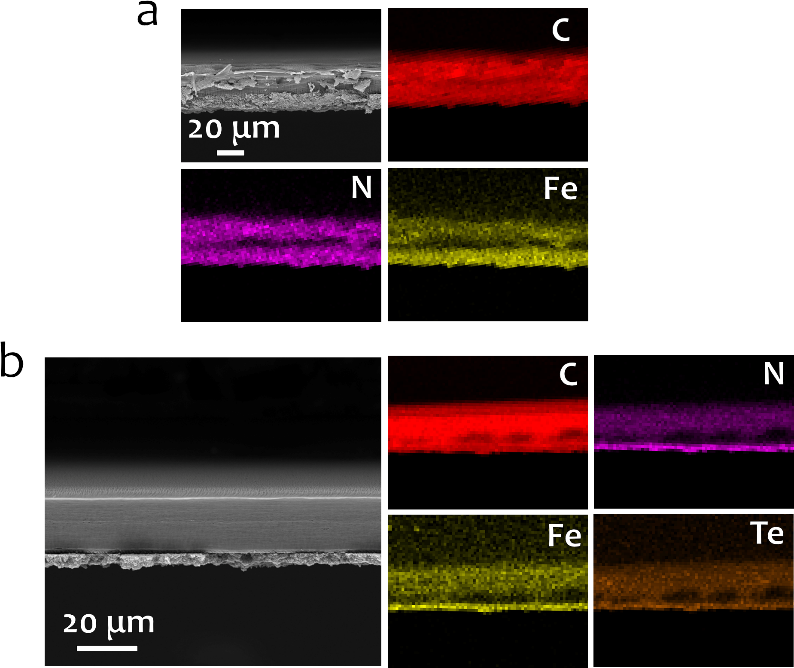


**Fig. S20** Cross-sectional SEM image and the corresponding elemental mapping images of (**a**) Fe/NC and (**b**) FeTe/NC modified separator

**Note:** The corresponding EDS mapping shows the uniform distribution of the components in the coating layer.


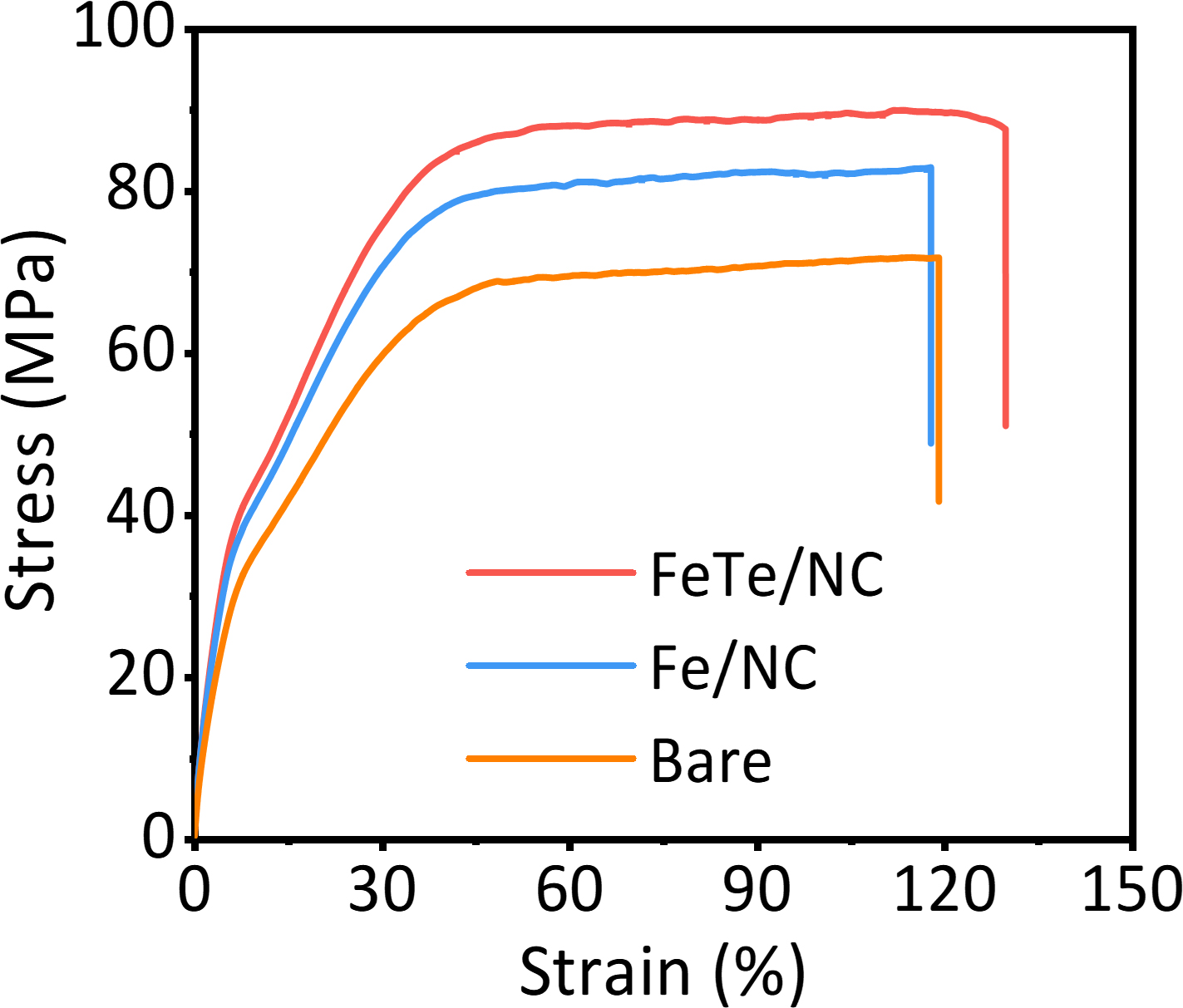


**Fig. S21** Stress-strain curves for different separators


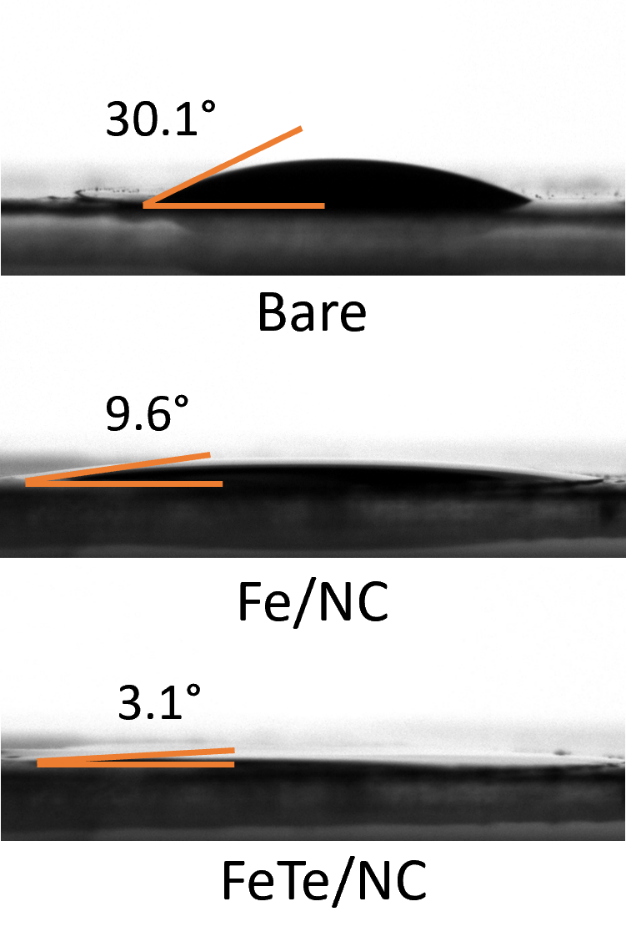


**Fig. S22** Contact angle measurement of Li-S electrolyte on the surface of the bare PP, Fe/NC, and FeTe/NC modified separators


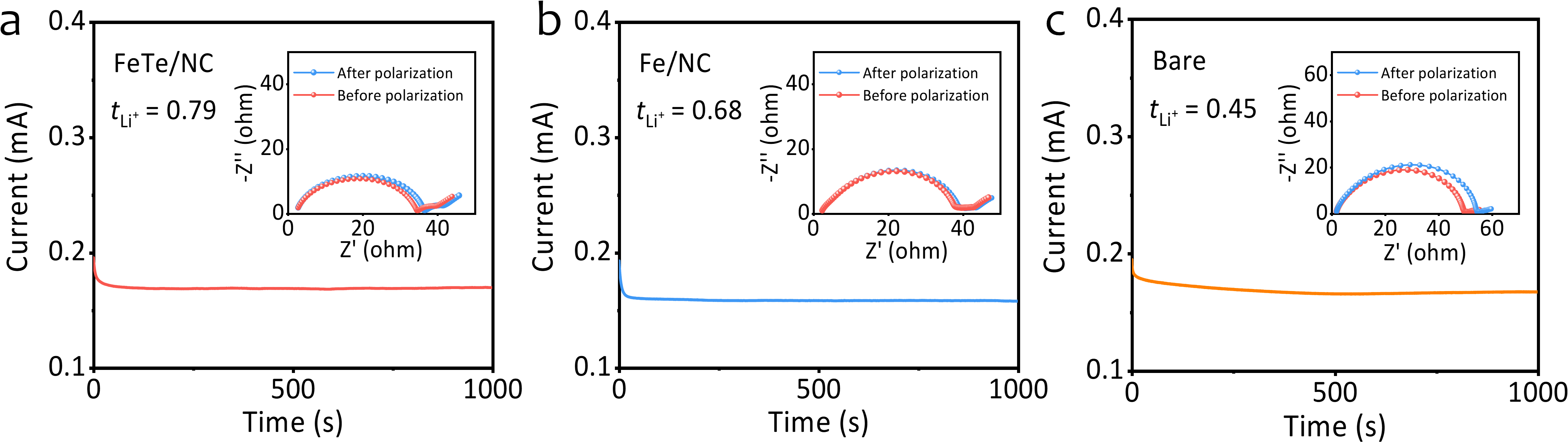


**Fig. S23** *I*-*t* and EIS curves of the FeTe/NC, Fe/NC and bare PP-based separators


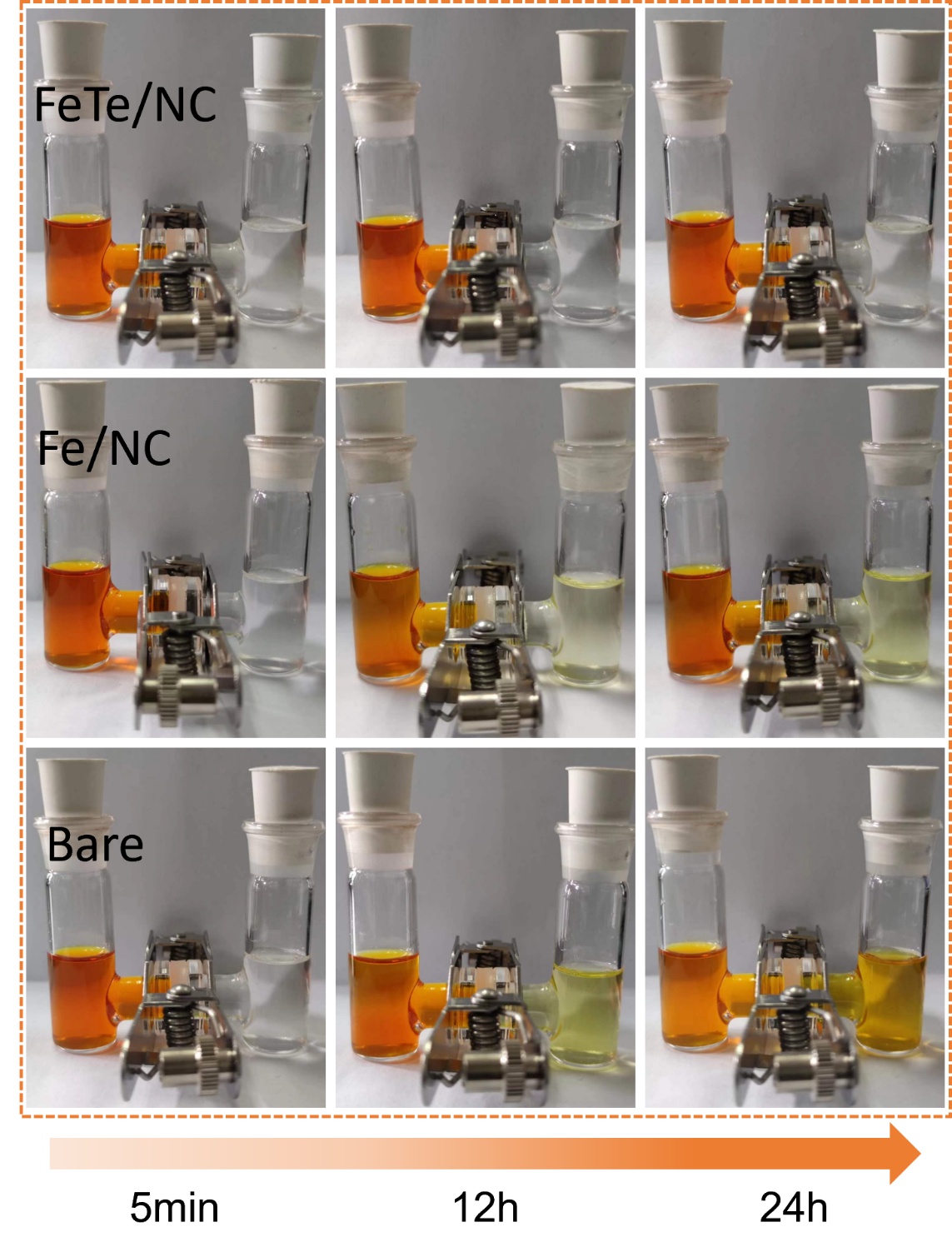


**Fig. S24** Diffusion tests of LiPSs with the bare PP, Fe/NC and FeTe/NC modified separators


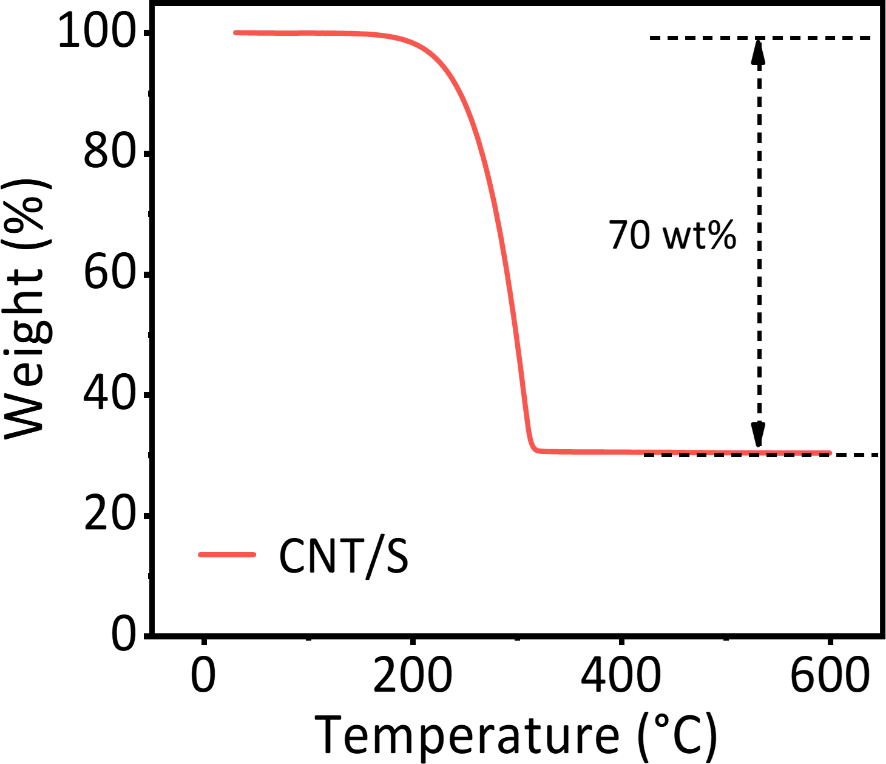


**Fig. S25** TG curve of CNT/S composite under nitrogen atmosphere


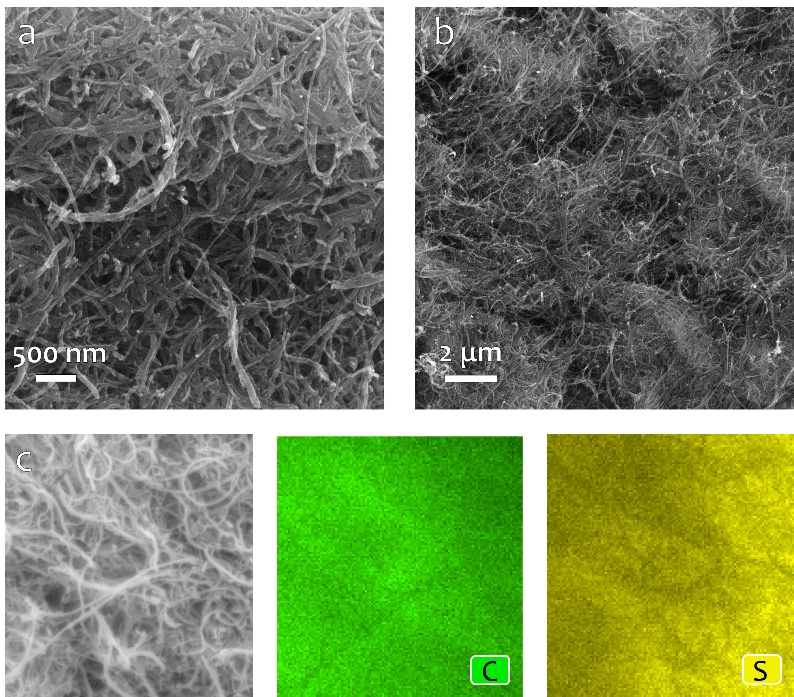


**Fig. S26** (**a-c**) SEM images and elemental mappings of CNT/S


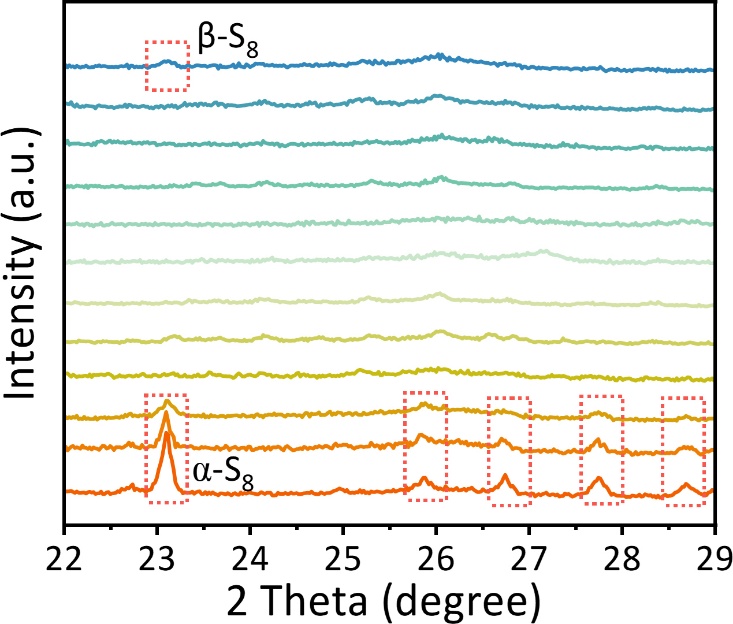


**Fig. S27** In-situ XRD patterns of the bare PP-based Li-S cells during the initial cycle


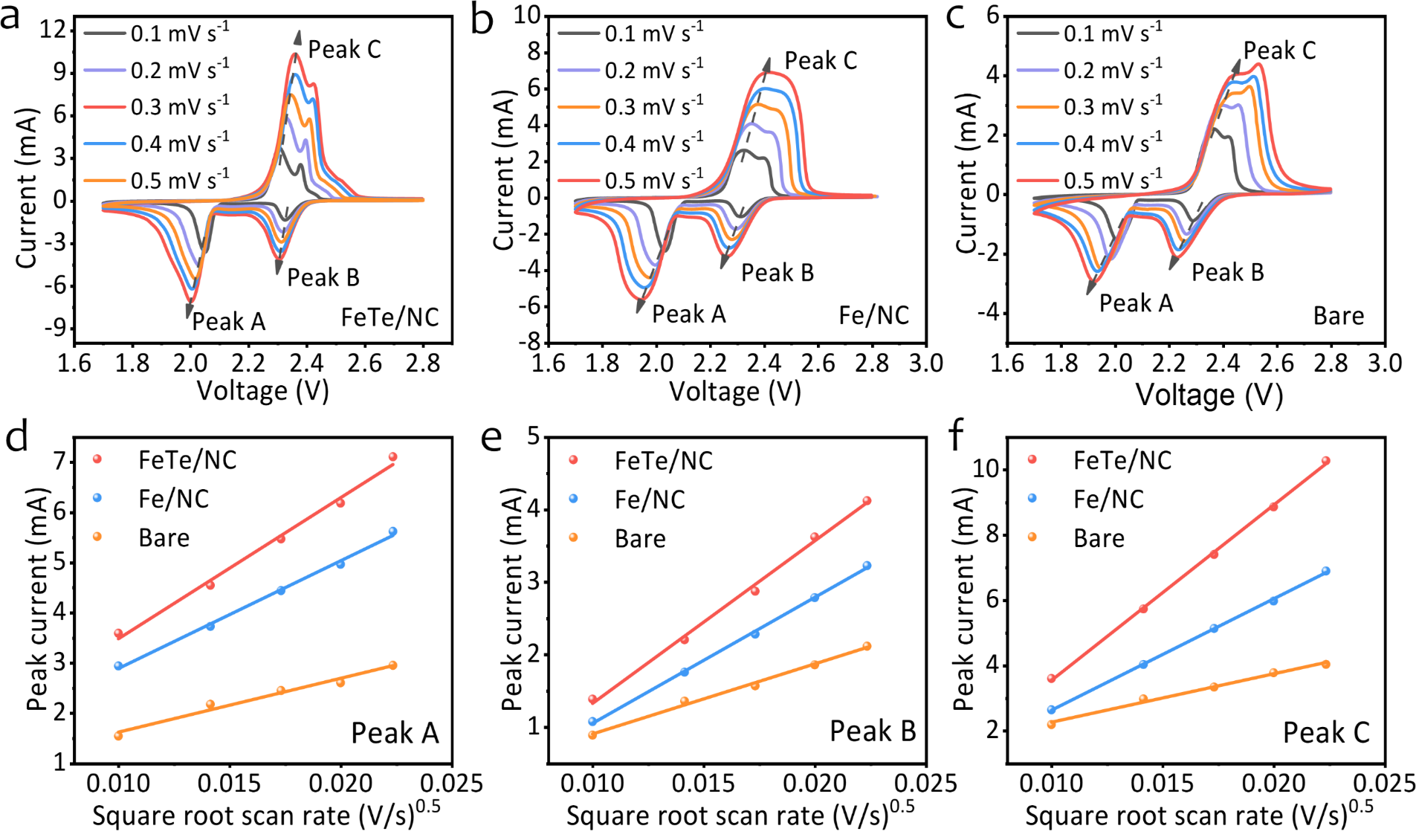


**Fig. S28** CV curves of the (**a**) FeTe/NC, (**b**) Fe/NC and (**c**) bare PP-based Li-S cells. The linear fitting results of the peak current as a function of scan rate with different separators at (**d**) reductive peak A, (**e**) reductive peak B and (**f**) oxidative peak C


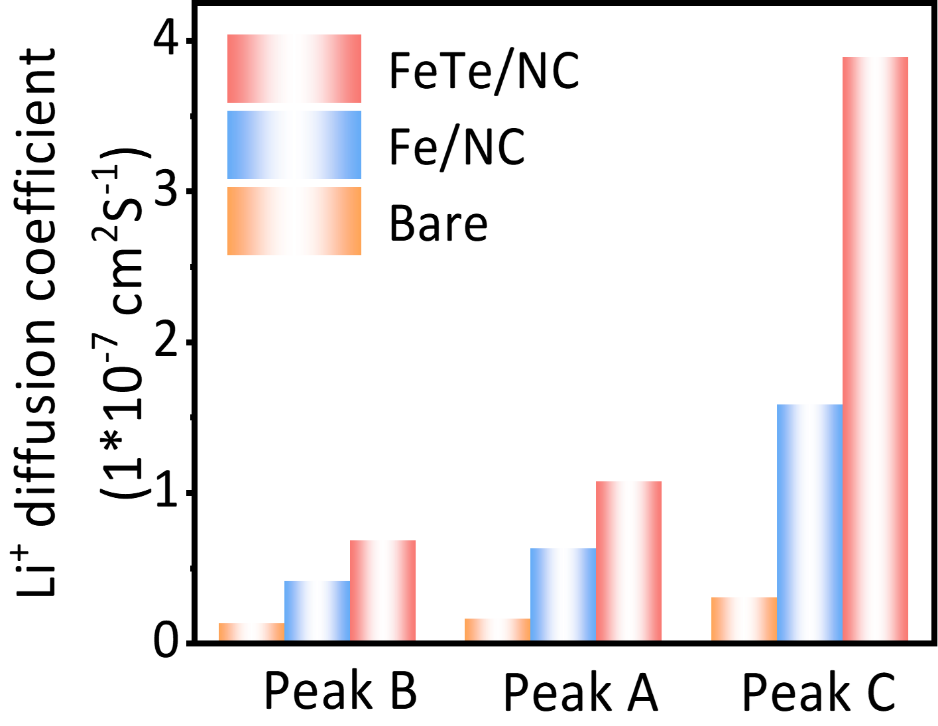


**Fig. S29** Li-ion diffusion coefficient value at peaks A, B, and C


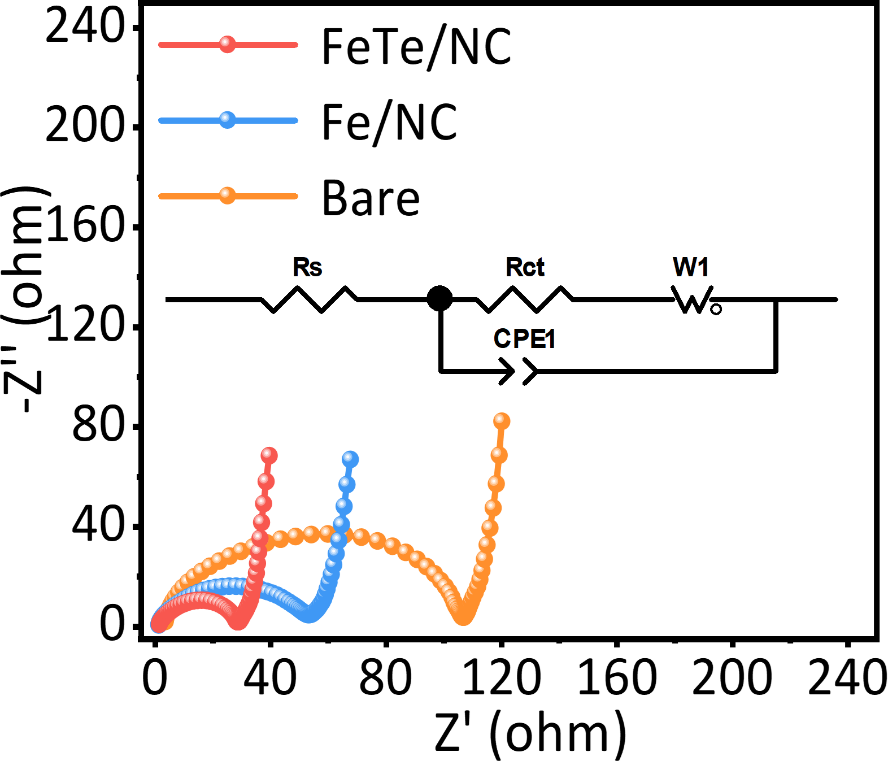


**Fig. S30** EIS plots of Li-S cells with different separators


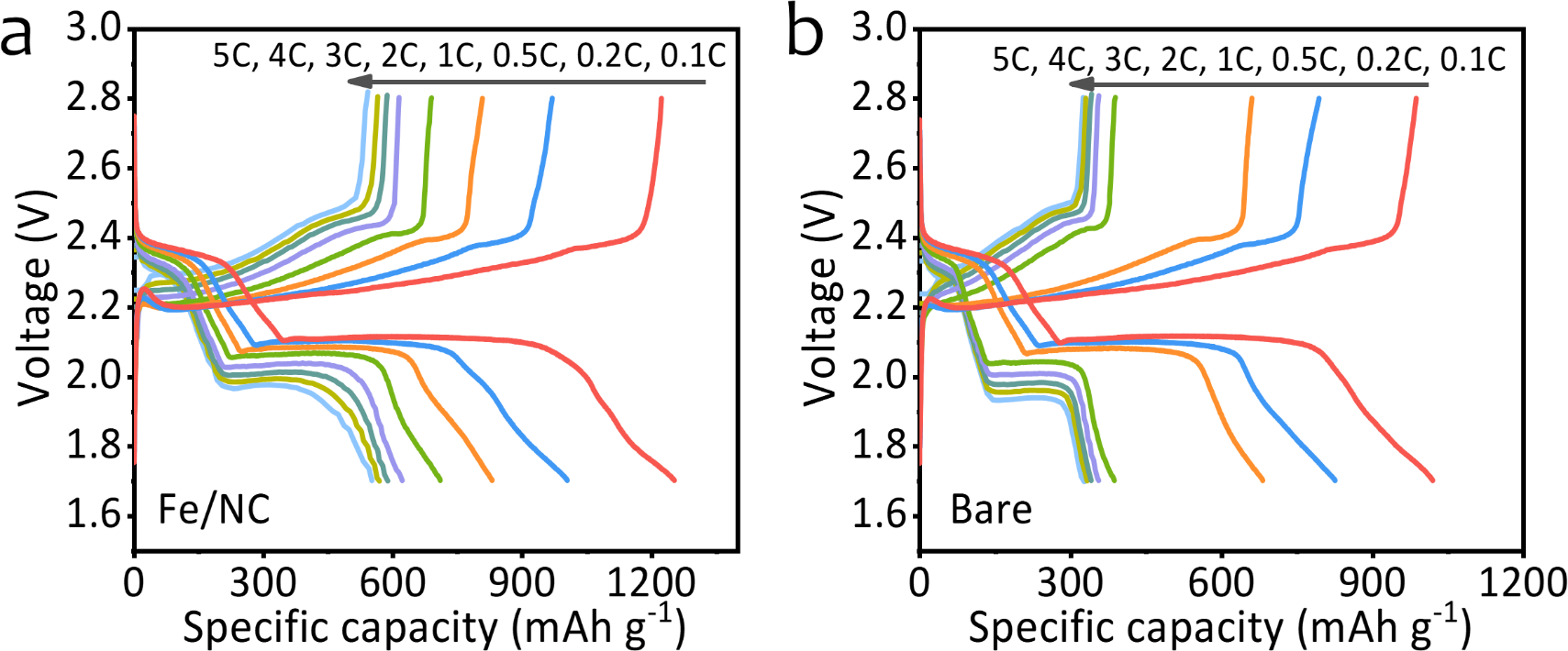


**Fig. S31** Discharge/charge profiles of the Li-S cells with (a) Fe/NC and (b) bare PP separators at different rates


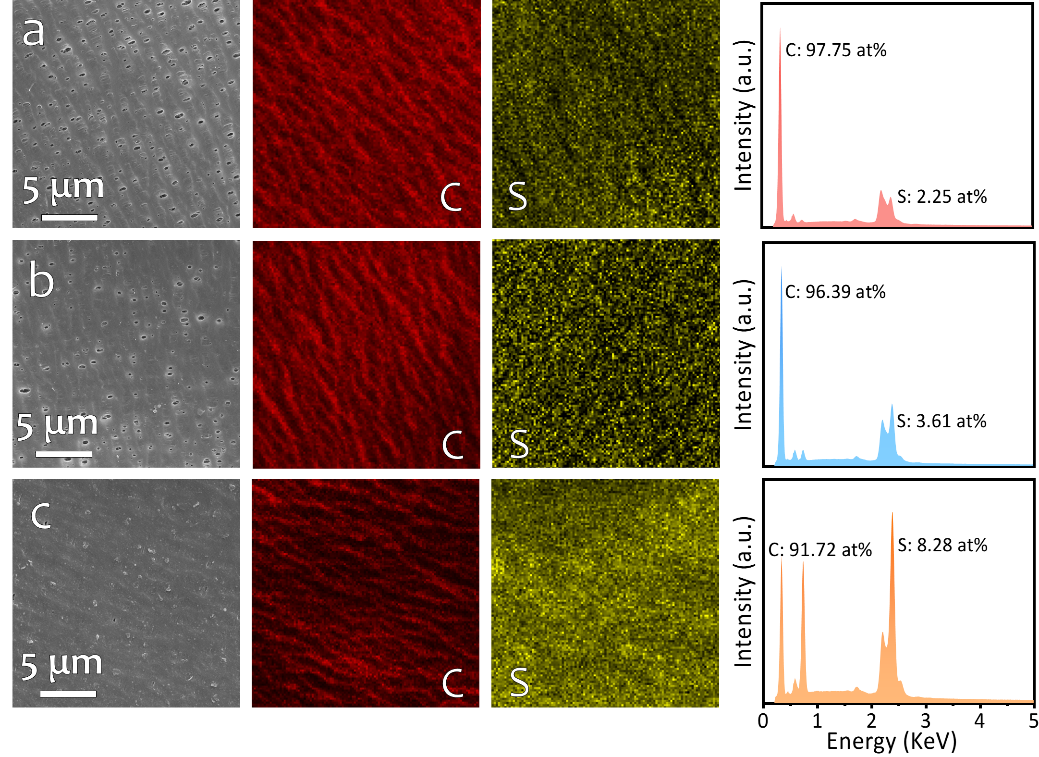


**Fig. S32** SEM images and the corresponding EDS spectra and elemental mapping images of the anode side of the (**a**) FeTe/NC, (**b**) Fe/NC, and (**c**) bare PP modified separators after 200 cycles at 1 C.

**Note:** The sulfur concentration of the bare PP separator on the anode side reached 8.28 at%, whereas it decreased to 3.61 at% for the Fe/NC modified separator and further to 2.25 at% for the FeTe/NC modified separator, highlighting the superior capability of the FeTe/NC coating to restrict LiPSs migration.


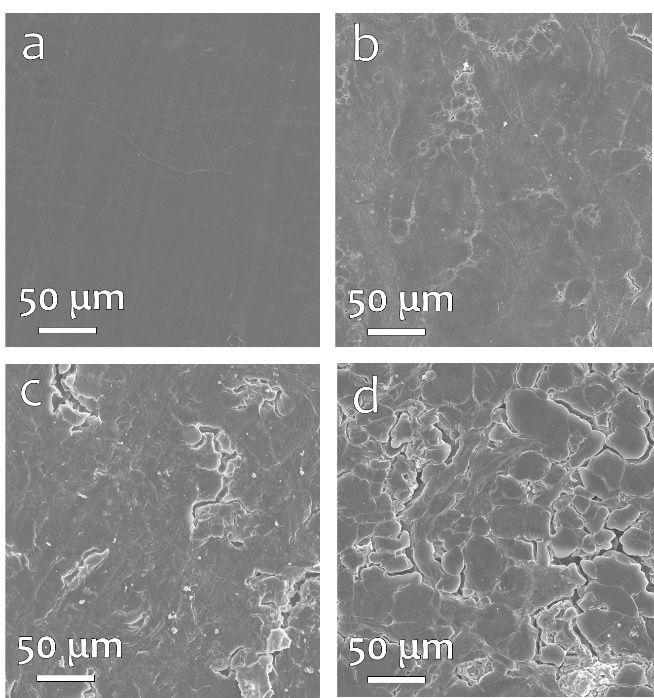


**Fig. S33** (**a**) Top-view morphology of fresh Li anode. Top-view morphology change of dissembled Li anodes after 200 cycles at 1 C in the (**b**) FeTe/NC, (**c**) Fe/NC, and (**d**) bare PP-based Li-S cells.

**Note:** The lithium anode with the bare PP separator exhibits a rough surface and a considerable amount of “dead” lithium after 200 cycles. In contrast, the anode paired with the FeTe/NC modified separator has a smoother morphology, likely resulting from residue and the decomposition of LiTFSI salt in the electrolyte. This suggests that the FeTe/NC catalyst effectively mitigates the dissolution and shuttling of LiPSs to the lithium metal side.


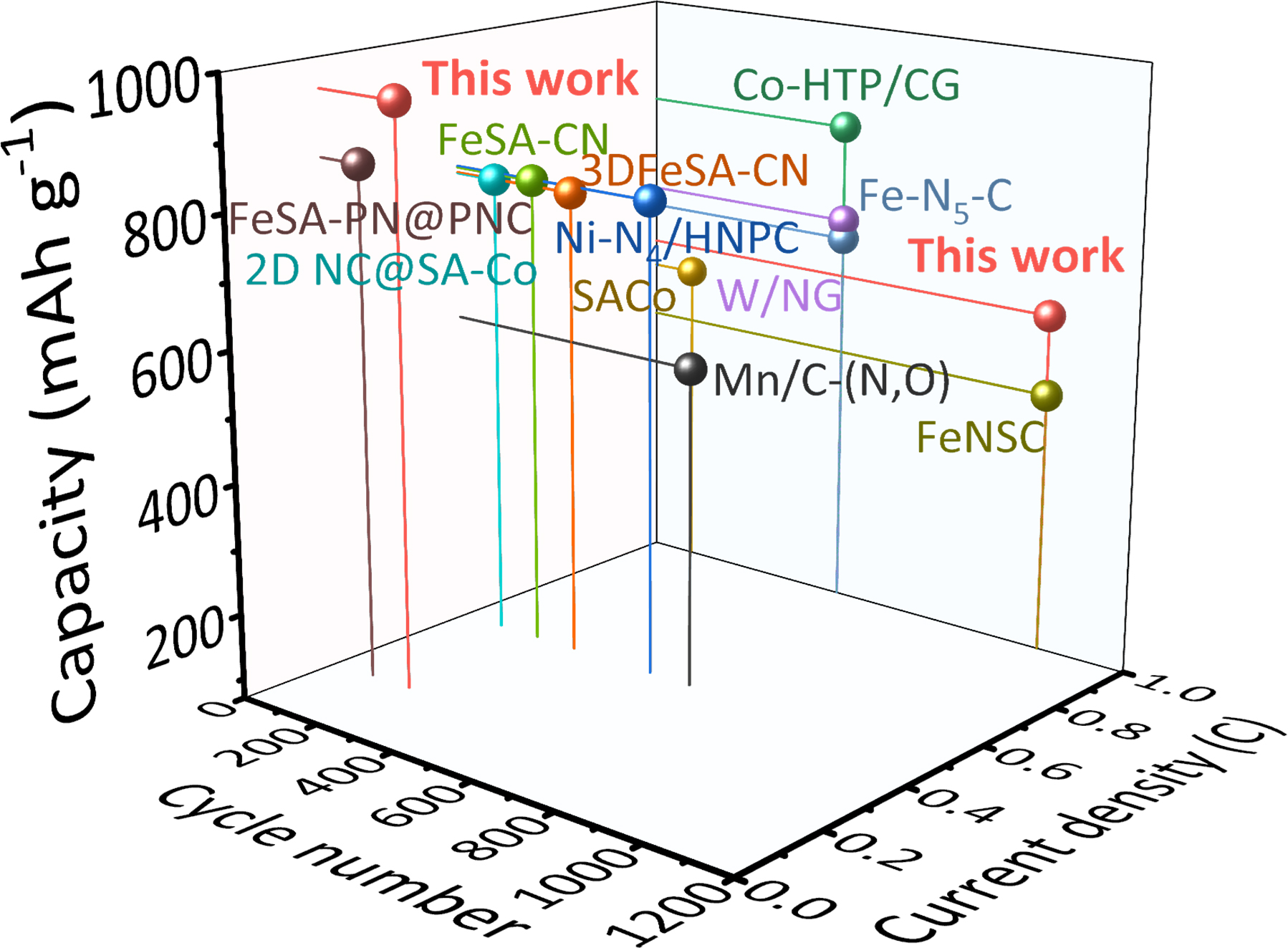


**Fig. S34** Performance comparison with reported metal atom-based catalysts in the Li-S battery


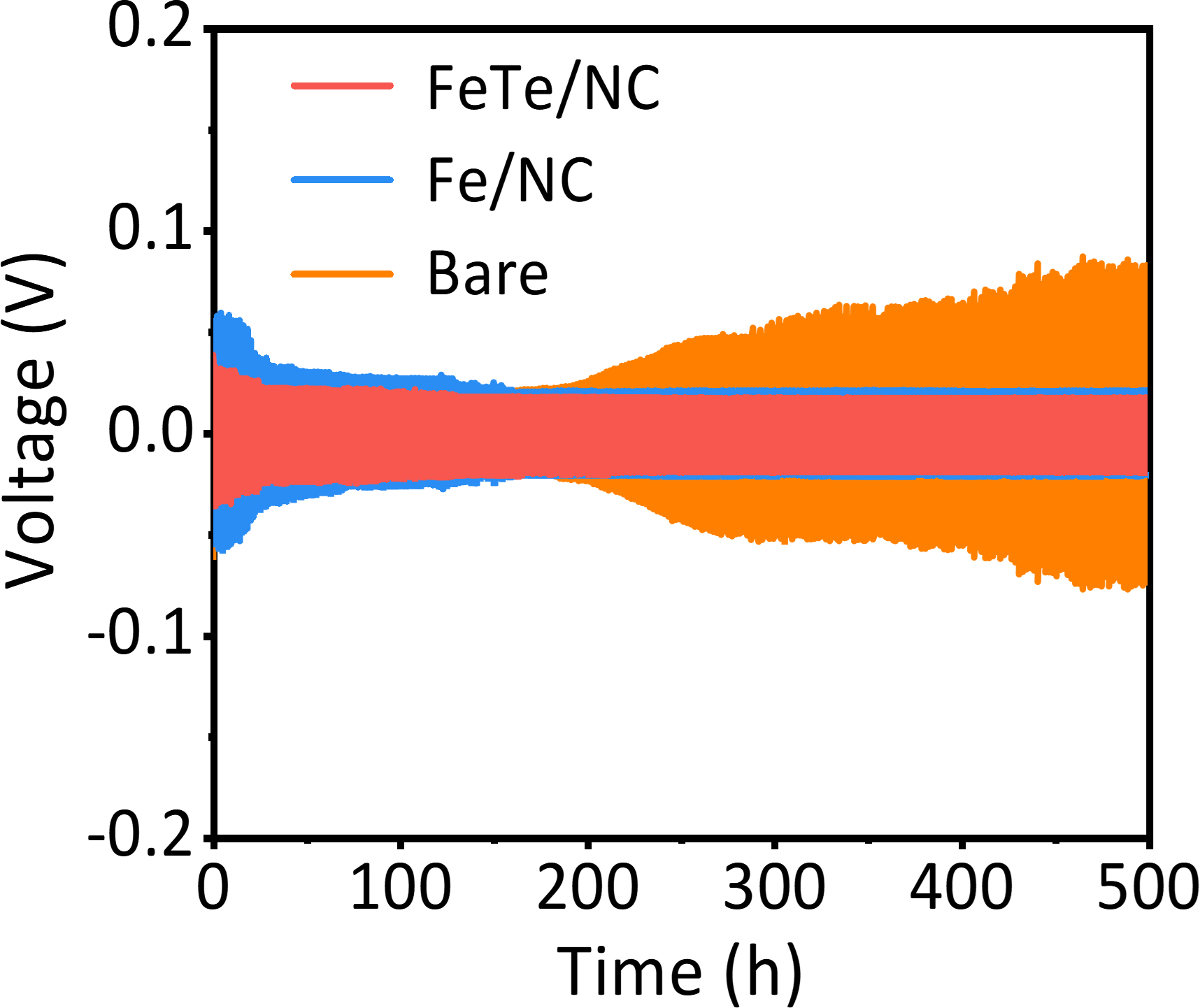


**Fig. S35** Cycling performance of Li-Li symmetric cells including FeTe/NC, Fe/NC, or bare PP-based separators at 1 mA cm^-2^ current for 1 mAh cm^-2^ capacity


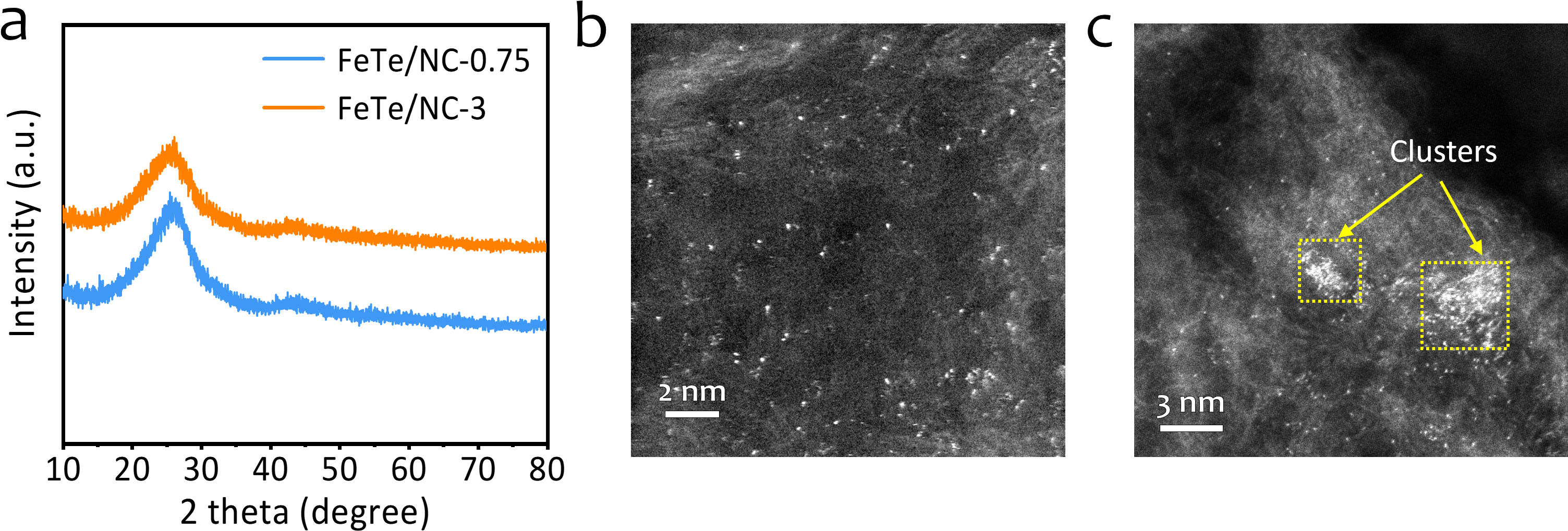


**Fig. S36** (**a**) XRD patterns, AC-HAADF-STEM images of (**b**) FeTe/NC-0.75 and (**c**) FeTe/NC-3


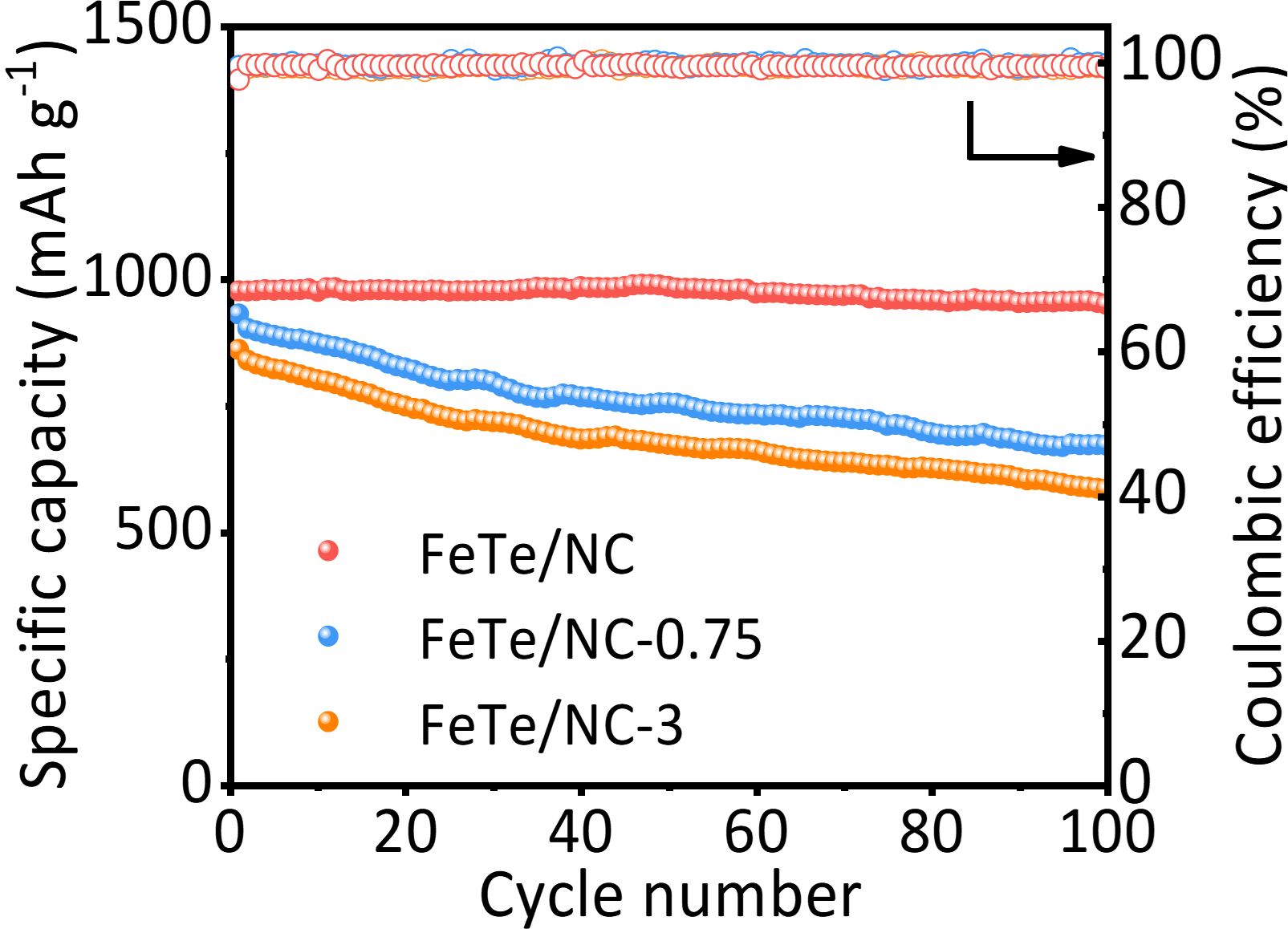


**Fig. S37** Cycling performance of FeTe/NC-0.75, FeTe/NC and FeTe/NC-3 based cells at 0.5 C


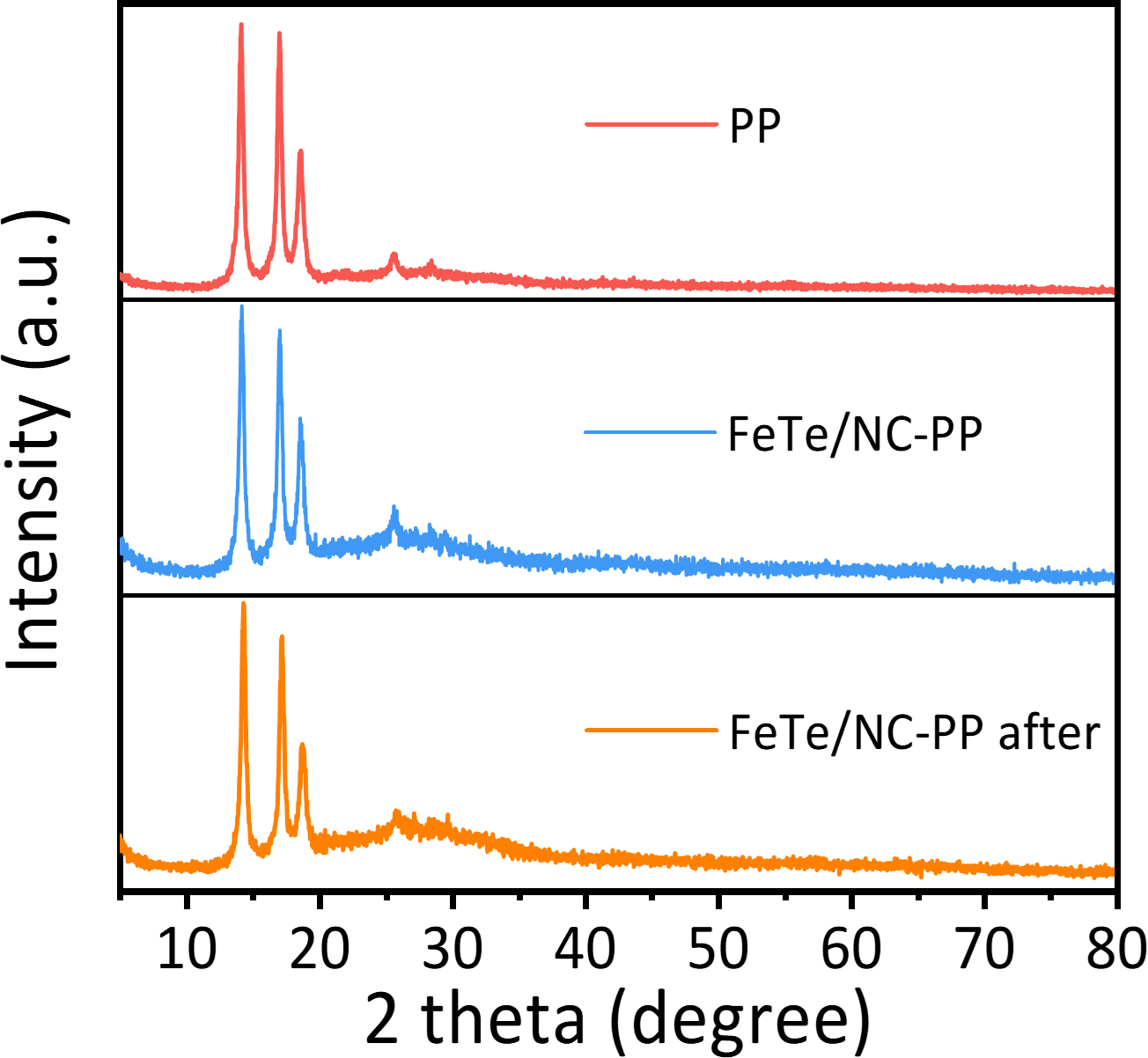


**Fig. S38** XRD patterns of pristine PP, FeTe/NC-PP and FeTe/NC-PP after 100 cycles at 0.5 C


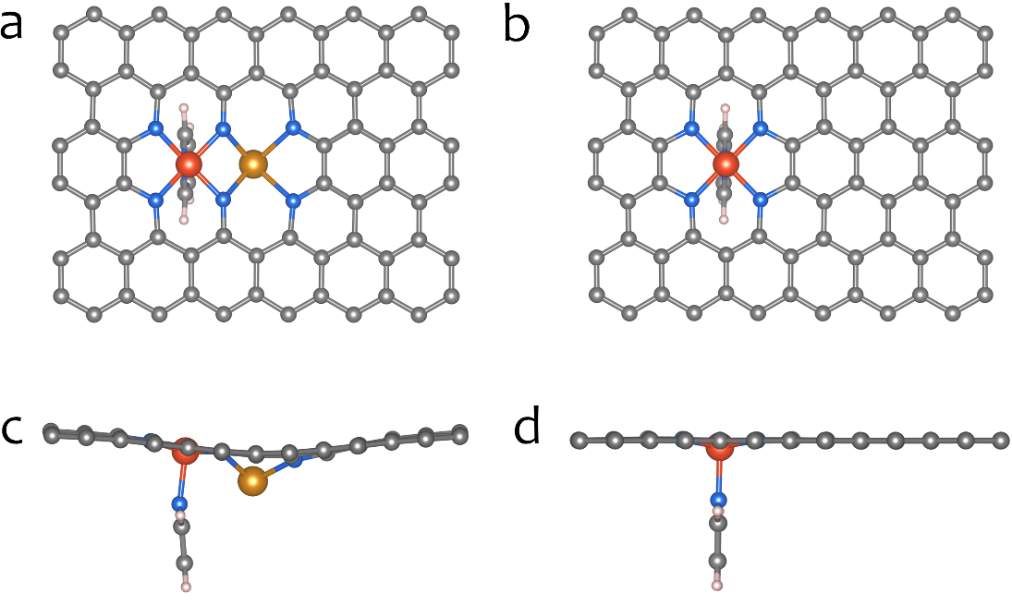


**Fig. S39** Optimized structures of (**a, c**) FeTe/NC, (**b, d**) Fe/NC


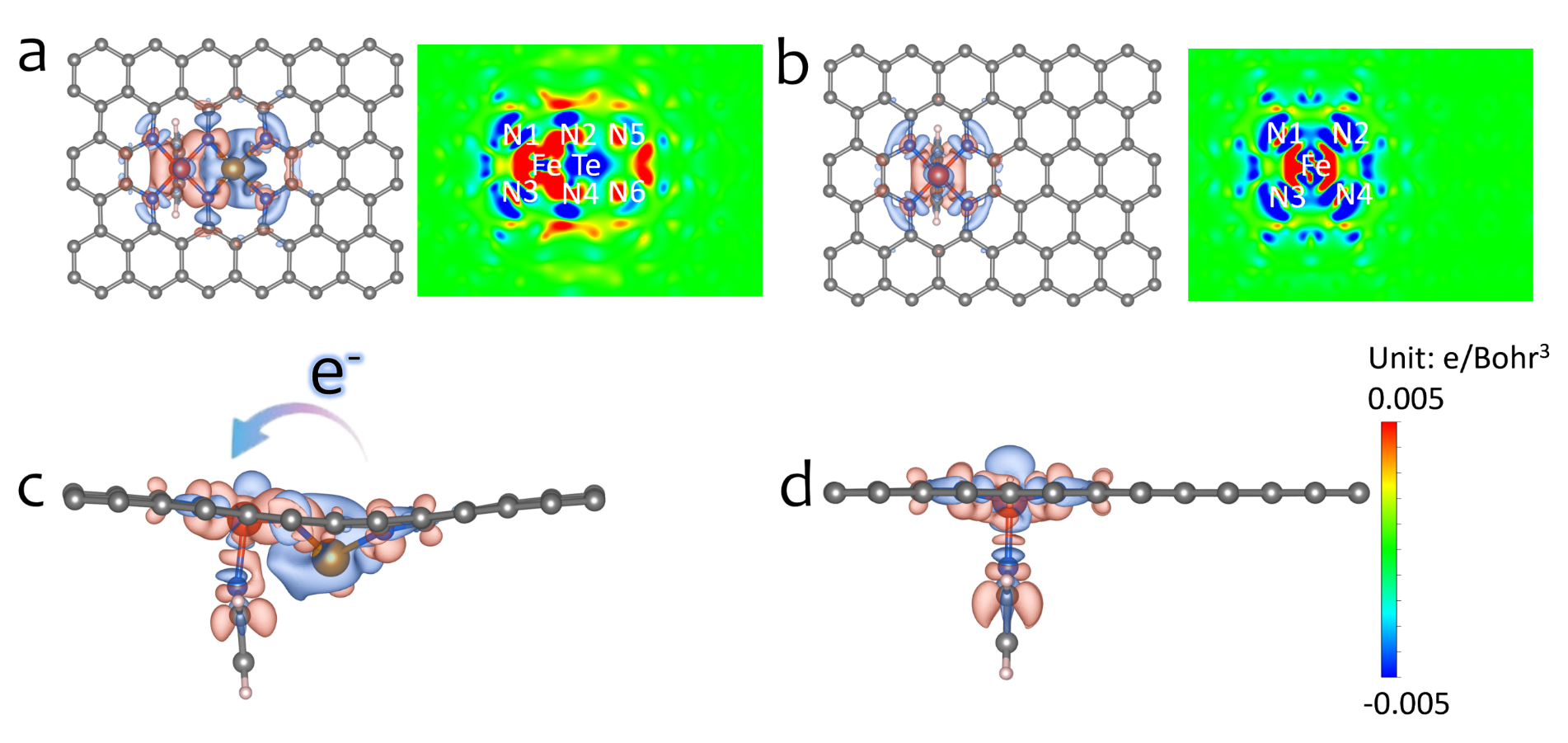


**Fig. S40** Three-dimensional (3D) charge density difference diagram of (a, c) FeTe/NC, (**b, d**) Fe/NC and corresponding 2D projection of charge density contour. (red: electron accumulation, blue: electron depletion, isovalue = 0.005 eV/Å^3^)


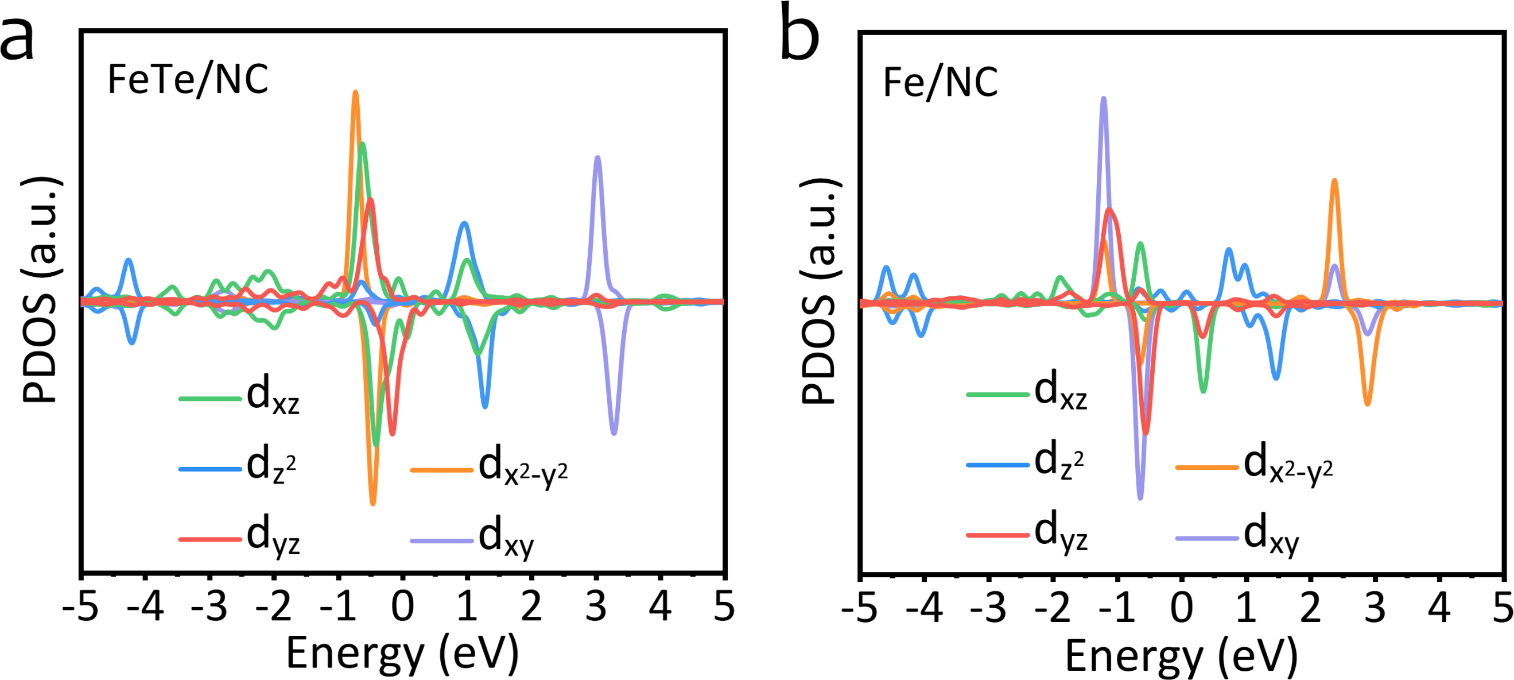


**Fig. S41** PDOS of Fe-d orbitals for the (**a**) FeTe/NC and (**b**) Fe/NC

**Note:** The d orbitals are divided into four categories: d_z_^2^, d_xy_, d_xz/yz_, and d_x_^2^_-y_^2^. Typically, the d_z_^2^ and d_xz/yz_ orbitals engage in hybridization with the p orbitals of sulfur atoms in LiPSs, leading to the formation of both bonding (𝜎 and 𝜋) and antibonding (𝜎* and 𝜋*) states. In contrast, the horizontal d orbitals (d_x_^2^_-y_^2^ and d_xy_) remain inactive and are considered nonbinding.[14] The geometric distortion in FeTe/NC raises the energy levels of the d_xz/yz_ orbitals compared to Fe/NC, which reduces the occupancy of the antibonding states, leading to stronger adsorption of LiPSs.


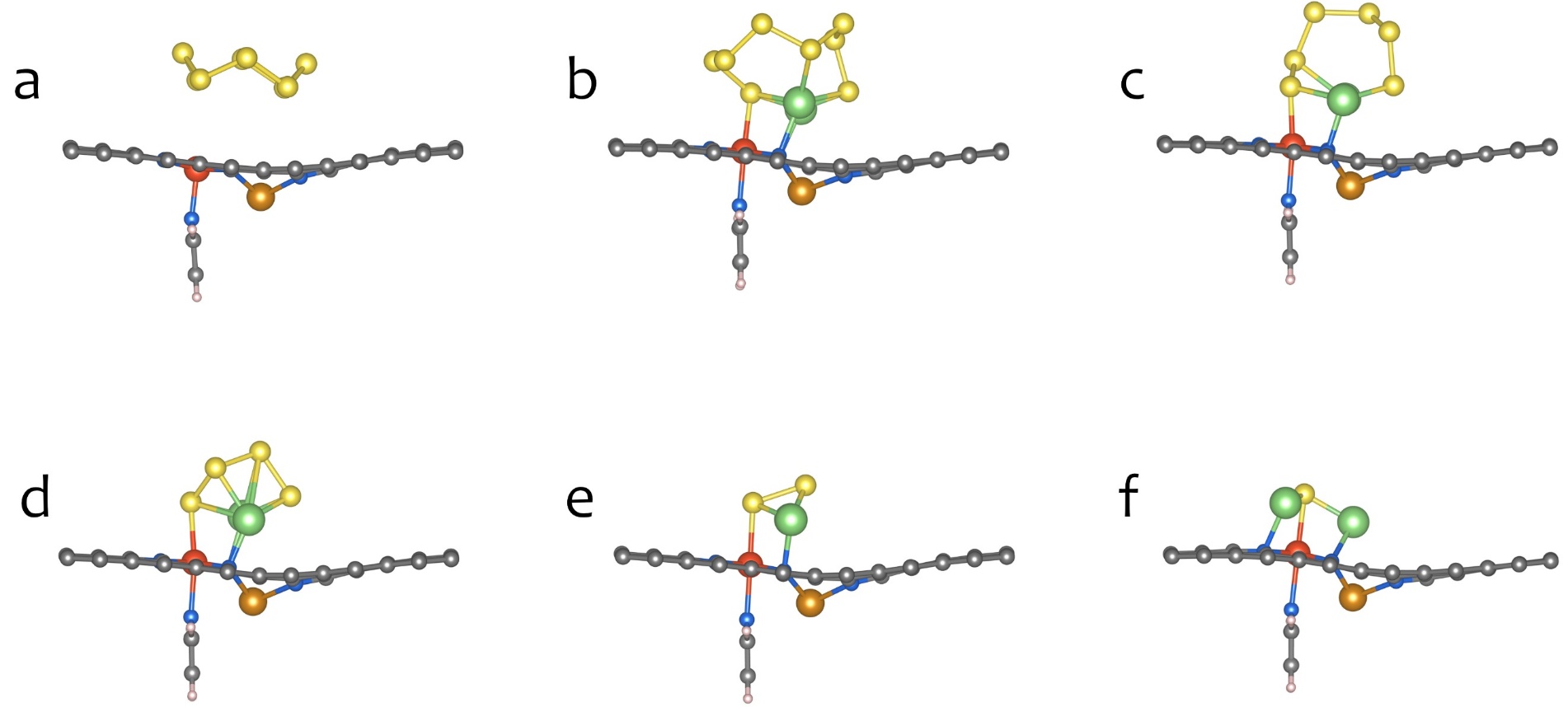


**Fig. S42** Optimized structures of (**a**) S_8_, (**b**) Li_2_S_8_, (**c**) Li_2_S_6_, (**d**) Li_2_S_4_, (e) Li_2_S_2_, (f) Li_2_S on FeTe/NC


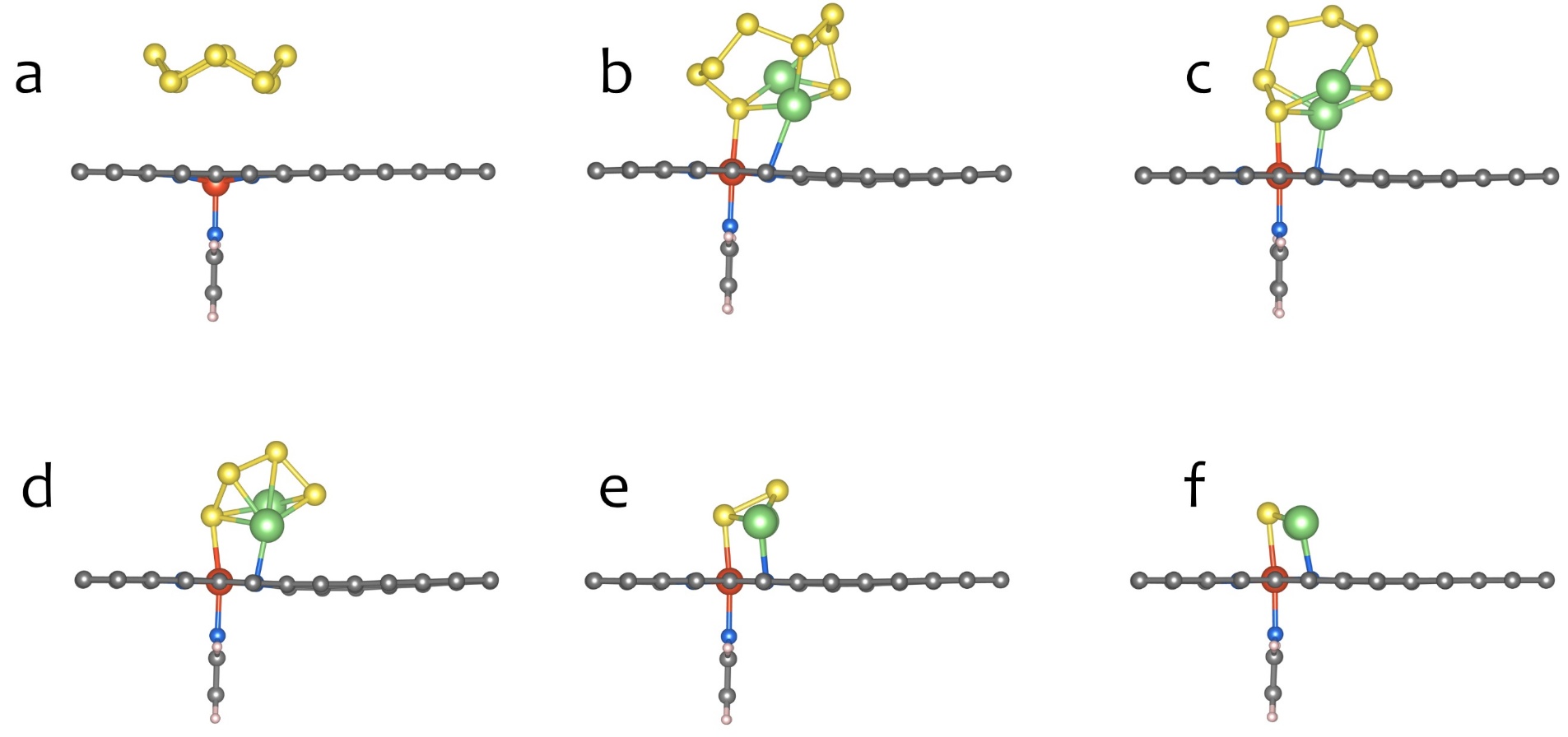


**Fig. S43** Optimized structures of (**a**) S_8_, (**b**) Li_2_S_8_, (**c**) Li_2_S_6_, (**d**) Li_2_S_4_, (**e**) Li_2_S_2_, (**f**) Li_2_S on Fe/NC


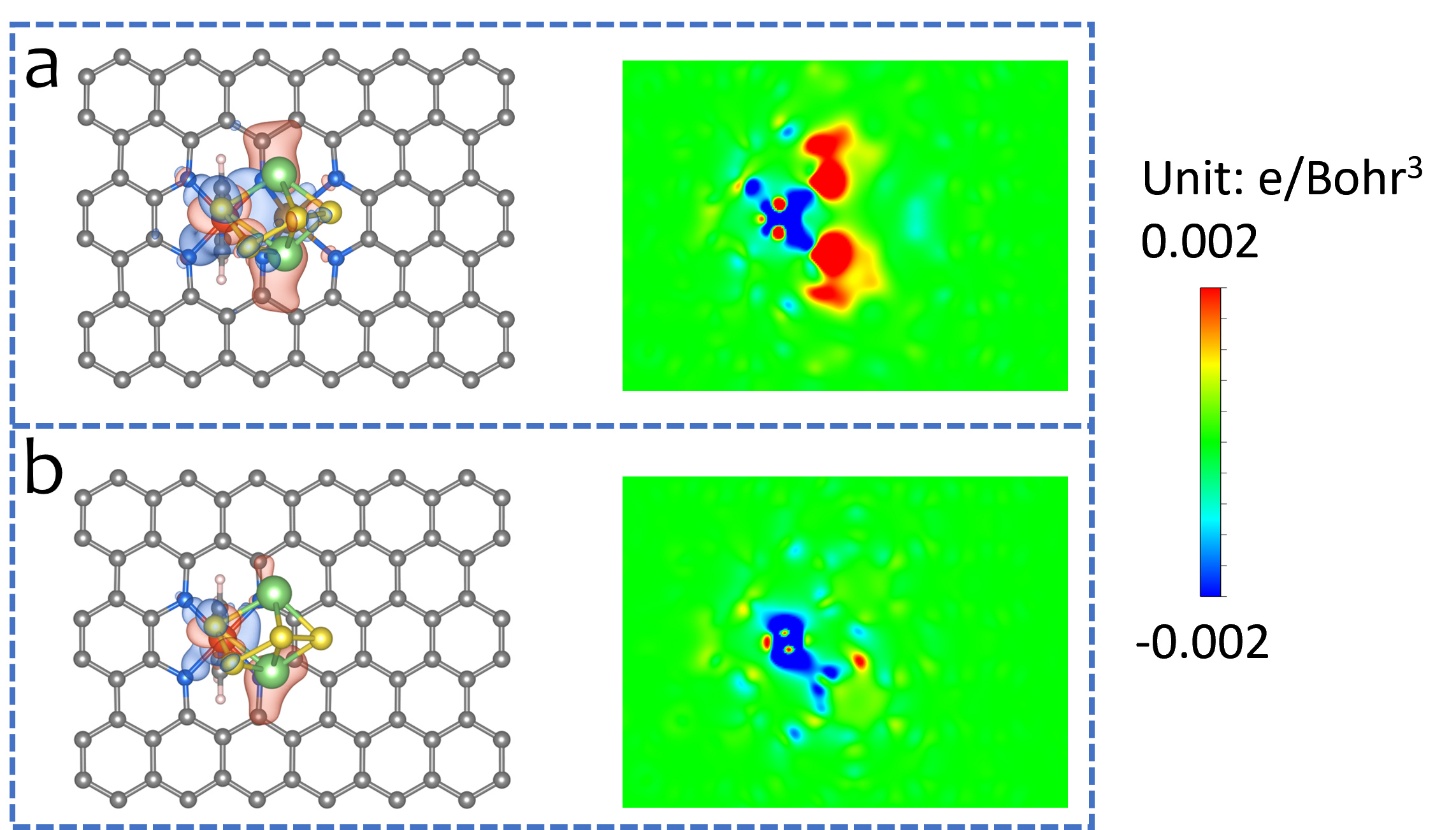


**Fig. S44** 3D charge density difference of Li_2_S_4_ adsorption on (**a**) FeTe/NC, (**b**) Fe/NC and corresponding 2D projection of charge density contour, the red (blue) distribution corresponds to charge accumulation (depletion)


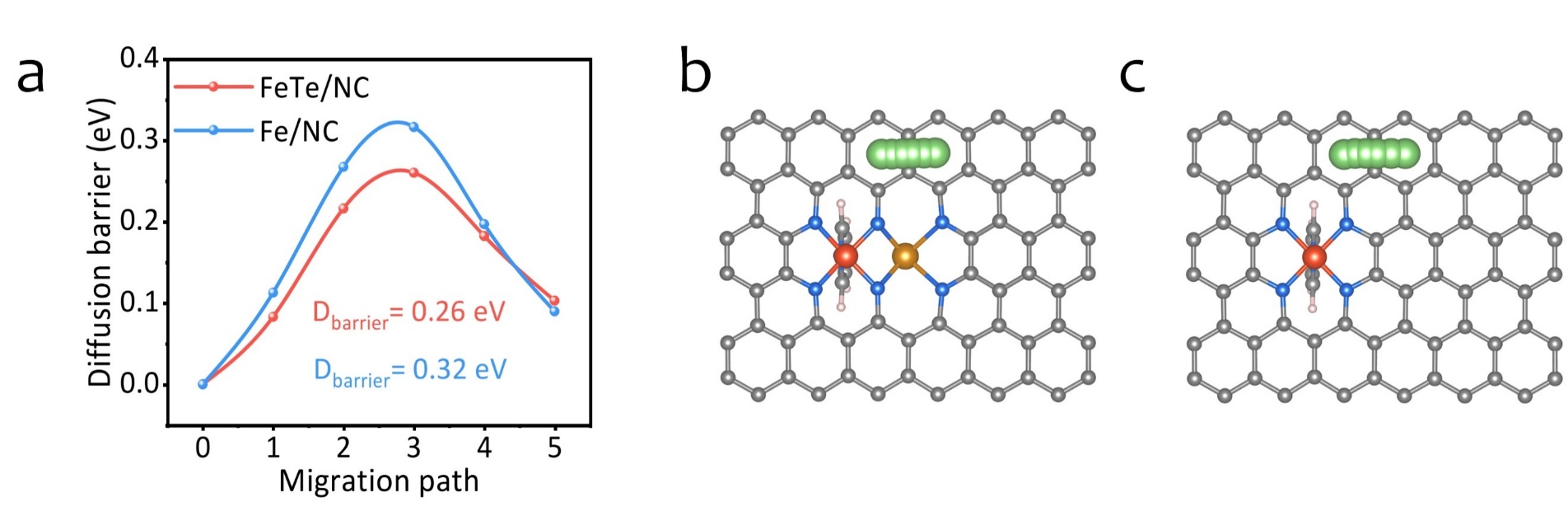


**Fig. S45** (**a**) Energy barrier of Li-ion diffusion on the surface of FeTe/NC and Fe/NC. Geometrical configurations of the minimum energy path of Li-ion diffusion on the surface of (**b**) FeTe/NC and (**c**) Fe/NC

**Table S1** ICP-OES results of different samples

| Samples | Elements | Element content in solution (mg L^-1^) | Element content in samples (wt%) | Atomic ratio |
| --- | --- | --- | --- | --- |
| FeTe/NC-0.75 | Fe | 3.1732 | 1.48% | Fe:Te = 2.2:1 |
|  | Te | 3.3650 | 1.57% |  |
| FeTe/NC | Fe  Te | 3.3025  6.2650 | 1.54%  2.92% | Fe:Te = 1.2:1 |
| FeTe/NC-3 | Fe | 3.4736 | 1.62% | Fe:Te = 1:1.5 |
|  | Te | 12.0921 | 5.64% |  |
| Fe/NC | Fe | 2.2601 | 1.04% |  |

**Table S2** EXAFS fitting parameters at the Fe K-edge and Te K-edge for FeTe/NC, Fe/NC, Te/NC, Fe foil and Te foil samples

| Samples | Path | CN | R(Å) | σ^2^×10^3^ (Å^2^) | ΔE_0_(eV) | R factor |
| --- | --- | --- | --- | --- | --- | --- |
| Fe/NC | Fe-N | 5.1±0.3 | 2.03±0.01 | 11.7±0.8 | 1.51±0.8 | 0.0092 |
| FeTe/NC | Fe-Te | 0.9±0.4 | 2.66±0.02 | 13.7±1.2 | 5.73±1.6 | 0.017 |
|  | Fe-N | 5.0±0.6 | 1.98±0.02 | 6.0±2.0 |  |  |
| Te/NC | Te-N | 2.9±0.5 | 1.91±0.02 | 5.8±1.7 | 4.99±1.6 | 0.015 |
| FeTe/NC | Te-Fe | 1.1±0.3 | 2.65±0.03 | 10.0±1.4 | 5.49±2.3 | 0.012 |
|  | Te-N | 4.0±0.4 | 2.0±0.02 | 6.5±1.5 |  |  |
| Fe-foil | Fe-Fe | 8* | 2.46±0.02 | 4.7±0.7 | 5.73±2.0 | 0.0026 |
|  | Fe-Fe | 6* | 2.85±0.01 | 5.8±1.2 |  |  |
| Te-foil | Te-Te | 2* | 2.83±0.01 | 5.5±1.3 | 7.33±1.2 | 0.014 |

**Note:** CN is the coordination number; R is the interatomic distance (the bond length between central atoms and surrounding coordination atoms); σ^2^ is the Debye−Waller factor (a measure of thermal and static disorder in absorber-scatterer distances); ΔE_0_ is edge energy shift (the difference between the zero kinetic energy value of the sample and that of the theoretical model). R factor is used to value the goodness of the fitting. * This value was fixed during EXAFS fitting, based on the known structure of Fe and Te. A reasonable range of EXAFS fitting parameters: 0.700 < *Ѕ*_0_^2^ < 1.000; *CN >* 0; *σ*^2^ > 0 Å^2^; |Δ*E*_0_| < 10 eV; *R* factor < 0.02.

**Table S3** Electrode resistances obtained from equivalent circuit fitting of FeTe/NC and Fe/NC symmetric cells.

| **Samples** | **R_s_/**Ω | **R_sei_/**Ω | **R_ct_/**Ω | **W_e_/**Ω |
| --- | --- | --- | --- | --- |
| **FeTe/NC** | 3.745 | 3.615 | 5.371 | 1.765 |
| **Fe/NC** | 3.524 | 6.583 | 5.963 | 2.148 |

**Table S4** The slope of the curve (*I*_p_/v^0.5^)

| **Samples** | **Peaki** | **Peakii** | **Peakiii** |
| --- | --- | --- | --- |
| **FeTe/NC** | 0.224 | 0.281 | 0.537 |
| **Fe/NC** | 0.174 | 0.215 | 0.342 |
| **Bare** | 0.097 | 0.107 | 0.148 |

**Table S5** Calculated lithium-ion diffusion coefficients (*D_Li_^+^*, cm^2^ s^-1^)

| **Samples** | **Peaki** | **Peakii** | **Peakiii** |
| --- | --- | --- | --- |
| **FeTe/NC** | 6.77×10^-8^ | 1.07×10^-7^ | 3.89×10^-7^ |
| **Fe/NC** | 4.09×10^-8^ | 6.24×10^-8^ | 1.58×10^-7^ |
| **Bare** | 1.27×10^-8^ | 1.55×10^-8^ | 2.96×10^-8^ |

**Table S6** Electrode resistances obtained from the equivalent circuit fitting of Li-S cells with the bare PP, Fe/NC and FeTe/NC modified separators.

| **Samples** | **R_s_/**Ω | **R_ct_/**Ω | **W_e_/**Ω |
| --- | --- | --- | --- |
| **FeTe/NC** | 1.42 | 26.81 | 5.09 |
| **Fe/NC** | 1.14 | 47.47 | 23.29 |
| **Bare** | 2.91 | 100.80 | 15.52 |

**Table S7** A comprehensive comparison of the electrochemical performance of the FeTe/NC modified separator in this work with metal atom-dispersed catalysts in the literature for advanced Li-S batteries

| Functional  materials | Coordination  structure | S mass loading  (mg cm^-2^) | Rate  (C) | Reversible Capacity  (mAh g^-1^) | Capacity  after cycling  (mAh g^-1^) | Refs. |  |
| --- | --- | --- | --- | --- | --- | --- | --- |
| **FeTe/NC** | **FeN_5_-TeN_4_** | **1.2** | **0.2**  **1**  **5** | **1149**  **974**  **735** | **954 (200th)**  **601 (1000th)**  **-** | **This work** |  |
| FeSA-CN | Fe-N_4_ | 1.4 | 0.2  0.5  4 | 1123  958  - | -  796 (200th)  605 (500th) | [S15] |  |
| Fe-PNC | Fe-N_4_ | 1.3 | 0.1  0.5 | -  - | 427 (300th)  557 (300th) | [S16] |  |
| Fe-N_5_-C | Fe-N_5_ | 1.0 | 0.2  1 | 1224  907 | 920 (100th)  662 (500th) | [S17] |  |
| 3DFeSA-CN | Fe-N_2_ | 1.2 | 0.5 | 1114 | 788 (300th) | [S18] |  |
| FeNSC | Fe-N_3_S_1_ | 1.0 | 1 | 905 | 477 (1000th) | [S19] |  |
| FeSA-PN@PNC | Fe-N_3_P_1_ | 1.5 | 0.2 | 1108 | 853(100th) | [S20] |  |
| S-SACo  @NG | Co-N_4_ | 2.0 | 0.5 | 749 | 551 (400th) | [S21] |  |
| 2D NC  @SA-Co | Co-N_4_ | 1.0 | 0.5 | - | 787 (100th) | [S22] |  |
| SACo | Co-N_3_ | 1.5-2.0 | 1 | 708 | 560 (100th) | [S23] |  |
| SA‐Co/BNC | | Co-B_2_N_2_ | 1.2 | 0.2  1 | 1257  1006 | 1106 (100th)  684 (1000th) | [S24] |
| Co-HTP/CG | | Co-N_4_O_1_ | 1.2-1.8 | 0.5  1 | 1070  1137 | 862 (200th)  840 (500th) | [S25] |
| CoSA-N_3_PS | | Co-N_3_P_1_S_1_ | - | 0.5  1  5 | 1098  -  - | 949 (200th)  -  - | [S26] |
| Ni-N_5_/  HNPC | Ni-N_5_ | 1.3-1.6 | 0.5 | 1086 | 798 (500th) | [S27] |  |
| Mn/C-  (N, O) | MnN_1_O_3_ | 1.1 | 0.5 | 1060 | 560 (600th) | [S28] |  |
| W/NG | W-O_2_N_2_ | 1.1 | 0.5  1  2 | -  -  - | 986 (200th)  691 (500th)  605 | [S29] |  |

**Table S8** Bader charges of FeTe/NC and Fe/NC

| **FeTe/NC** | | **Fe/NC** | |
| --- | --- | --- | --- |
| **Atom** | **Charge** | **Atom** | **Charge** |
| N1 | -1.17 | N1 | -1.18 |
| N2 | -1.17 | N2 | -1.17 |
| N3 | -1.16 | N3 | -1.17 |
| N4 | -1.16 | N4 | -1.17 |
| N5 | -1.23 | Fe | +1.21 |
| N6 | -1.23 | - | - |
| Fe | +1.15 | - | - |
| Te | +1.84 | - | - |

**Supplementary References**

1. B. Wang, L. Wang, D. Ding et al., Zinc-assisted cobalt ditelluride polyhedra inducing lattice strain to endow efficient adsorption-catalysis for high-energy lithium-sulfur batteries. Adv. Mater. **34**(50), 2204403 (2022). <https://doi.org/10.1002/adma.202204403>
2. K. Liu, J. Feng, J. Guo et al., (1‐10) facet‐dominated TiB_2_ nanosheets with high exposure of dual‐atom‐sites for enhanced polysulfide conversion in Li-S batteries. Adv. Funct. Mater. **34**(17), 2314657 (2023). <https://doi.org/10.1002/adfm.202314657>
3. X. B. Wang, C. R. Zhao, B. X. Liu et al., Creating edge sites within the 2D metal-organic framework boosts redox kinetics in lithium-sulfur batteries. Adv. Energy Mater. **12**(42), 2201960 (2022). <https://doi.org/10.1002/aenm.202201960>
4. W. Q. Yao, W. Z. Zheng, J. Xu et al., ZnS-SnS@NC heterostructure as robust lithiophilicity and sulfiphilicity mediator toward high-rate and long-life lithium-sulfur batteries. ACS Nano **15**(4), 7114-7130 (2021). <https://doi.org/10.1021/acsnano.1c00270>
5. P. E. Blöchl. Projector augmented-wave method. Phys. Rev. B **50**(24), 17953-17979 (1994). <https://doi.org/10.1103/PhysRevB.50.17953>
6. G. Kresse, J. Furthmuller. Efficiency of ab-initio total energy calculations for metals and semiconductors using a plane-wave basis set. Comp. Mater. Sci. **6**(1), 15-50 (1996). <https://doi.org/10.1016/0927-0256(96)00008-0>
7. G. Kresse, J. Hafner. Ab initiomolecular-dynamics simulation of the liquid-metal–amorphous-semiconductor transition in germanium. Phys. Rev. B **49**(20), 14251-14269 (1994). <https://doi.org/10.1103/PhysRevB.49.14251>
8. M. Ernzerhof, G. E. Scuseria. Assessment of the perdew-burke-ernzerhof exchange-correlation functional. J. Chem. Phys. **110**(11), 5029-5036 (1999). <https://doi.org/10.1063/1.478401>
9. G. Kresse, D. Joubert. From ultrasoft pseudopotentials to the projector augmented-wave method. Phys. Rev. B **59**(3), 1758-1775 (1999). <https://doi.org/10.1103/PhysRevB.59.1758>
10. S. Grimme, S. Ehrlich, L. Goerigk. Effect of the damping function in dispersion corrected density functional theory. J. Comput. Chem. **32**(7), 1456-1465 (2011). <https://doi.org/10.1002/jcc.21759>
11. G. Henkelman, B. P. Uberuaga, H. Jónsson. A climbing image nudged elastic band method for finding saddle points and minimum energy paths. J. Chem. Phys. **113**(22), 9901-9904 (2000). <https://doi.org/10.1063/1.1329672>
12. Z. Y. Chen, X. Z. Su, J. Ding et al., Boosting oxygen reduction reaction with Fe and Se dual-atom sites supported by nitrogen-doped porous carbon. Appl. Catal. B-Environ. Energy **308**(121206 (2022). <https://doi.org/10.1016/j.apcatb.2022.121206>
13. Y. Tian, G. Li, Y. Zhang et al., Low‐bandgap Se‐deficient antimony selenide as a multifunctional polysulfide barrier toward high‐performance lithium–sulfur batteries. Adv. Mater. **32**(4), 1904876 (2019). <https://doi.org/10.1002/adma.201904876>
14. Q. Lv, Y. Sun, B. Li et al., Metal–organic frameworks with axial cobalt–oxygen coordination modulate polysulfide redox for lithium–sulfur batteries. Adv. Energy Mater. **15**(5), 2403223 (2024). <https://doi.org/10.1002/aenm.202403223>
15. C. G. Wang, H. W. Song, C. C. Yu et al., Iron single-atom catalyst anchored on nitrogen-rich mof-derived carbon nanocage to accelerate polysulfide redox conversion for lithium sulfur batteries. J. Mater. Chem A. **8**(6), 3421-3430 (2020). <https://doi.org/10.1039/c9ta11680j>
16. Z. Z. Liu, L. Zhou, Q. Ge et al., Atomic iron catalysis of polysulfide conversion in lithium-sulfur batteries. ACS Appl. Mater. Interfaces **10**(23), 19311-19317 (2018). <https://doi.org/10.1021/acsami.8b03830>
17. Y. G. Zhang, J. B. Liu, J. Y. Wang et al., Engineering oversaturated Fe-N multifunctional catalytic sites for durable lithium-sulfur batteries. Angew. Chem. Int. Ed. **60**(51), 26622-26629 (2021). <https://doi.org/10.1002/anie.202108882>
18. Y. F. Ding, Q. S. Cheng, J. H. Wu et al., Enhanced dual-directional sulfur redox via a biotemplated single-atomic Fe-N mediator promises durable Li-S batteries. Adv. Mater. **34**(28), 2202256 (2022). <https://doi.org/10.1002/adma.202202256>
19. H. Zhao, B. Tian, C. Su, Y. Li. Single-atom iron and doped sulfur improve the catalysis of polysulfide conversion for obtaining high-performance lithium–sulfur batteries. ACS Appl. Mater. Interfaces **13**(6), 7171-7177 (2021). <https://doi.org/10.1021/acsami.0c20446>
20. T. Huang, Y. J. Sun, J. H. Wu et al., Altering local chemistry of single-atom coordination boosts bidirectional polysulfide conversion of Li-S batteries. Adv. Funct. Mater. **32**(39), 2203902 (2022). <https://doi.org/10.1002/adfm.202203902>
21. G. M. Zhou, S. Y. Wang, T. S. Wang et al., Theoretical calculation guided design of single-atom catalysts toward fast kinetic and long-life Li-S batteries. Nano Lett. **20**(2), 1252-1261 (2020). <https://doi.org/10.1021/acs.nanolett.9b04719>
22. Y. J. Li, P. Zhou, H. Li et al., A freestanding flexible single-atom cobalt-based multifunctional interlayer toward reversible and durable lithium-sulfur batteries. Small Methods **4**(3), 1900701 (2020). <https://doi.org/10.1002/smtd.201900701>
23. J. Kim, S. J. Kim, E. Jung et al., Atomic structure modification of Fe-N-C catalysts via morphology engineering of graphene for enhanced conversion kinetics of lithium-sulfur batteries. Adv. Funct. Mater. **32**(19), 2110857 (2022). <https://doi.org/10.1002/adfm.202110857>
24. Z. Wang, Y. Yan, Y. Zhang et al., Single-atomic Co-B_2_N_2_ sites anchored on carbon nanotube arrays promote lithium polysulfide conversion in lithium–sulfur batteries. Carbon Energy **5**(11), e306 (2023). <https://doi.org/10.1002/cey2.306>
25. Q. L. Lv, Y. J. Sun, B. Li et al., Metal-organic frameworks with axial cobalt-oxygen coordination modulate polysulfide redox for lithium-sulfur batteries. Adv. Energy Mater. **15**(5), 2403223 (2024). https://doi.org/10.1002/aenm.202403223
26. C. X. Dong, C. N. Ma, C. Zhou et al., Engineering d-p orbital hybridization with P, S co-coordination asymmetric configuration of single atoms toward high-rate and long-cycling lithium-sulfur battery. Adv. Mater. **36**(38), 2407070 (2024). <https://doi.org/10.1002/adma.202407070>
27. S. L. Zhang, X. Ao, J. Huang et al., Isolated single-atom Ni-N catalytic site in hollow porous carbon capsules for efficient lithium-sulfur batteries. Nano Lett. **21**(22), 9691-9698 (2021). <https://doi.org/10.1021/acs.nanolett.1c03499>
28. Y. N. Liu, Z. Y. Wei, B. Zhong et al., O-, N-coordinated single Mn atoms accelerating polysulfides transformation in lithium-sulfur batteries. Energy Storage Mater. **35**(12-18 (2021). <https://doi.org/10.1016/j.ensm.2020.11.011>
29. P. Wang, B. J. Xi, Z. C. Y. Zhang et al., Atomic tungsten on graphene with unique coordination enabling kinetically boosted lithium-sulfur batteries. Angew. Chem. Int. Ed. **60**(28), 15563-15571 (2021). <https://doi.org/10.1002/anie.202104053>
